# Supplementary material for: Molecular modularity and asymmetry of the molluscan mantle revealed by a gene expression atlas
Source: Gigascience. 2018 May 17;7(6):giy056. doi: 10.1093/gigascience/giy056 (PMC6007483; doi:10.1093/gigascience/giy056)
Supplement: Additional_file_47.docx [file giy056_additional_file_47.docx]

#### Extended Results and Discussion for:

**Molecular modularity and asymmetry of the molluscan mantle revealed by a gene expression atlas**

Ines Herlitze^1^, Benjamin Marie^2^, Frédéric Marin^3^, Daniel J. Jackson^1*^

^1^ Department of Geobiology, Georg-August University of Göttingen, Goldschmidtstrasse 3, 37077 Göttingen, Germany

^2^ UMR 7245 MNHN/CNRS Molécules de Communication et Adaptation des Micro-organismes, Département Aviv, Sorbonne Universités, Muséum National d’Histoire Naturelle, CP 39, 12 Rue Buffon, 75005 Paris, France.

^3^ UMR CNRS 6282 Biogéosciences, Université de Bourgogne - Franche-Comté, 6 Boulevard Gabriel, 21000 Dijon, France

***Author for correspondence:**

Daniel J. Jackson

Department of Geobiology

Georg-August University of Göttingen

Goldschmidtstrasse 3, 37077

Göttingen, Germany

Tel: +49 (0) 55139 14177

email: [djackso@uni-goettingen.de](mailto:djackso@uni-goettingen.de)

**Extended Results and Discussion**

The following extended results and discussion sections provide individual descriptions for the spatial expression patterns and general molecular features of each of the 34 shell-forming genes we have identified and characterised from *Lymnaea stagnalis.*

***Lstag-sfc-1 (L. stagnalis-shell-forming candidate-1)***

Expression of *Lstag*-*sfc-1* was restricted to the right side of the shell field in all larval stages (Additional file 2Ai’- Aiv’). *Lstag*-*sfc-1* was expressed in cells of the lower right quarter of the non-invaginated cells of the shell gland in larvae two to three dpfc (Additional file 2Ai, Ai’). Larvae three to six dpfc showed expression in the right-hand shell-field margin (Additional file 2Aii, Aii’, Aiii, Aiii’). The mantle margin that lines the right side of the aperture displayed *Lstag*-*sfc-1* expression in larvae seven dpfc (Additional file 2Aiv, Aiv’). The adult mantle displayed expression of *Lstag-sfc-1* in zones one and two (Additional file 2Av, Av’).

The encoded 352 aa long protein carries a signal sequence and is predicted to have six glycosylation sites (Additional file 2B, Additional file 37). The most abundant amino acids are alanine (10.5%), valine (9.7%), leucine (9.1%) and asparagine (8.0%) and the theoretical pI is 4.68 (Additional file 37). Transcripts that encode proteins similar to *Lstag*-sfc-1 are apparently unique to the Mollusca (Additional file 43).

***Lstag-sfc-2 (L. stagnalis-shell-forming candidate-2)***

*Lstag-sfc-2* was expressed in a few cells bordering the shell gland margin within larvae two to three dpfc (Additional file 3Ai, Ai’). Larvae three to six dpfc displayed asymmetric expression of *Lstag-sfc-2* on the right half of the shell field margin (Additional file 3Aii, Aii’, Aiii, Aiii’). Larvae seven dpfc were stained along the mantle margin that lines the right side of the shell aperture (Additional file 3Aiv, Aiv’). The adult mantle displayed expression in zones one and two (Additional file 3Av, Av’).

Two contigs are potential targets for the riboprobe that generated the staining pattern presented in Additional file 3. The 414 aa long protein encoded by contig 77206 shows sequence similarity with ‘early nodulin-12A’ (Additional files 37 and 40). *Lstag*-sfc-2 has a signal sequence, 12 putative glycosylation sites, three repeated motifs and a pI of 5.12 (Additional file 3B, and 37). The most abundant amino acids are alanine (13.0%), serine (9.2%) and valine (8.0%) (Additional file 37). The protein encoded by contig 77215 is 234 aa long, has no signal sequence, two potential glycosylation sites, one repeated motif and a theoretical pI of 4.63 (Additional file 37). The most abundant amino acids are serine (11.1%), alanine (9.8%), valine (9.0%) and aspartic acid (8.5%)(Additional file 37).

***Lstag-sfc-3 (L. stagnalis-shell-forming candidate-3)***

Larvae younger than five dpfc showed no expression of *Lstag-sfc-3* (Additional file 4Ai, Ai’, Aii, Aii’), however a clear signal in the right side of the mantle margin in larvae five to six dpfc was detected (Additional file 4Aiii, Aiii’). The mantle margin in larvae seven dpfc showed expression in the mantle margin that lines the right side of the aperture (Additional file 4Aiv, Aiv’). The adult mantle displayed staining in zone one and two (Additional file 4Av, Av’).

The 393 aa long *Lstag*-sfc-3 protein has a signal sequence, two repeated motifs, 15 putative glycosylation sites, a theoretical pI of 5.47 and anomalous serine (11.2%), alanine (10.4%), asparagine (10.2%) and glycine (9.2%) contents (Additional file 4B, Additional file 37).

Our mantle, cephalic tentacle, cephalic lobe and CNS transcriptomes contain *Lstag-sfc-3* contigs that could be mapped to the draft genome (Additional file 4C). The mantle zone 5 and cephalic tentacle transcriptomes also contain alternatively spliced *Lstag-sfc-3* transcripts. Strikingly, the 5’ UTR sequence of the transcript present in mantle zones 1-5 is alternately spliced resulting in a unique and longer 5’ UTR (Additional file 4C).

***Lstag-sfc-4 (L. stagnalis-shell-forming candidate-4)***

No expression of *Lstag-sfc-4* could be detected in larvae younger than five dpfc (Additional file 5Ai, Ai’, Aii, Aii’), however a signal was monitored along the right side of the shell field margin within larvae five to six dpfc (Additional file 5Aiii, Aiii’) and along the anterior parts of the mantle margin in larvae seven dpfc (Additional file 5Aiv, Aiv’). The adult mantle showed a signal at the posterior wall of the mantle groove in zone 1 (Additional file 5Av, Av’).

The 149 aa long *Lstag*-sfc-4 protein has a signal sequence, a theoretical pI of 5.38 and anomalous glycine (12.1%) and cysteine (8.1%) contents (Additional file 5B, Additional file 37). Lstag-sfc-4 is also predicted to bind chitin (Additional file 37). Transcripts encoding proteins with sequences similar to *Lstag*-sfc-4 are present in a broad range of metazoan phyla (Additional file 43).

Contigs coding for the protein presented in Additional file 5B are present in the transcriptomes of the mantle, CNS and buccal mass and could be mapped to the draft genome (Additional file 5C). These contigs vary in their 3’ and 5’ UTR lengths (Additional file 5C).

***Lstag-sfc-5 (L. stagnalis-shell-forming candidate-5)***

*Lstag-sfc-5* was expressed in approximately three quarters of the cells bordering the shell gland (from 2 o’clock to 10 o’clock) in larvae two to three dpfc (Additional file 6Ai, Ai’). In larvae three to six dpfc expression of *Lstag-sfc-5* was present in all cells lining the shell field margin (Additional file 6Aii, Aii’, Aiii, Aiii’). After seven dpfc, larvae displayed *Lstag-sfc-5* expression in the mantle margin that lines the left and right side of the aperture (Additional file 6Aiv, Aiv’). The adult mantle displayed a positive *Lstag-sfc-5* signal in zones one and two (Additional file 6Av, Av’).

The 645 aa long *Lstag*-sfc-5 protein shares sequence similarity with a number of peroxidases (Additional file 37). A conserved animal haem peroxidase domain comprises the majority of the protein, which has a signal sequence, nine predicted glycosylation sites and a theoretical pI of 7.82 (Additional file 6B, Additional file 37). The predicted molecular functions are haem binding and peroxidase activity (Additional file 37). Transcripts encoding similar proteins are present in many taxa across a broad range of metazoans (Additional file 43).

***Lstag-sfc-6 (L. stagnalis-shell-forming candidate-6)***

*Lstag-sfc-6* displayed expression in a symmetrical ring within the non-invaginated cells bordering the shell gland in larvae two to three dpfc (Additional file 7Ai, Ai’). In larvae three to six dpfc a positive signal within the elongated cells of the shell field margin was also detected (Additional file 7Aii-Aiii, Aiii’-Aiii’). Larvae seven dpfc displayed expression in the part of the mantle margin that lines the left side of the aperture (Additional file 7Aiv, Aiv’). The adult mantle showed a distinct and clear signal in zone three (Additional file 7Av, Av’).

Two transcripts are potential targets for the riboprobe that generated the expression pattern presented in Additional file 7A. They code for proteins that vary in only six amino acids (131 aa and 137 aa) (Additional file 7B and C). Both proteins are primarily composed of glycine (54.2% and 54.7% respectively) and leucine (15.3% each), each has a signal sequence, a basic pI of 9.22 and is dominated by a glycine-rich repeated motif (Additional file 7B, C, Additional file 37).

***Lstag-sfc-7 (L. stagnalis-shell-forming candidate7)***

Larvae two to three dpfc displayed *Lstag-sfc-7* staining in a symmetrical ring within the non-invaginated cells bordering the shell gland (Additional file 8AI, Ai’). The cells along the shell field margin displayed strong expression within larvae three to six dpfc (Additional file 8Aii, Aii’, Aiii, Aiii’). Larvae seven dpfc express *Lstag-sfc-7* in the part of the mantle margin on the left side of the aperture (Additional file 8Aiv, Aiv’). Similar to *Lstag-sfc-6, Lstag-sfc-7* displayed a distinct and clear signal in zone three (Additional file 8Av, Av’).

The 111 aa long *Lstag*-sfc-7 protein is rich in glycine (47.7%) and tyrosine (14.2%) and is dominated by a glycine-rich repeated motif. It has a signal sequence and a theoretical pI of 9.4 (Additional file 8B, Additional file 37).

***Lstag-sfc-8 (L. stagnalis-shell-forming candidate-8)***

*Lstag-sfc-8* displayed no expression in larvae younger than seven dpfc (Additional file 9Ai- Aiii, Ai’- Aiii’). Larvae about seven dpfc showed *Lstag-sfc-8* expression in the mantle edge along the left side of the shell aperture (Additional file 9Aiv, Aiv’). Similar to *Lstag-sfc-6* and *Lstag-sfc-7, Lstag-sfc-8* displayed a distinct and clear signal in zone three (Additional file 9Av, Av’).

The *Lstag*-sfc-8 protein is 112 aa long and rich in glycine (50.0%), tyrosine (12.5%) and leucine (10.7%). The basic protein (pI 8.83) has a signal sequence and is dominated by a glycine-rich repeated motif (Additional file 9B, Additional file 37). Contigs coding for the protein presented in Additional file Fig. 9B are present in the transcriptomes of the mantle, cephalic tentacle and larval stages and could be mapped to the draft genome (Additional file 9C). These transcripts vary in their 5’ UTR lengths. Strikingly the contig present in the mantle zone 1-5 has a longer 5’ UTR (Additional file 9C).

***Lstag-sfc-9 (L. stagnalis-shell-forming candidate-9)***

*Lstag-sfc-9* was expressed in cells bordering the shell gland (approximately 3 o’clock to 7 o’clock) in larvae two to three dpfc (Additional file 10Ai, Ai’). Larvae three to four dpfc showed *Lstag-sfc-9* expression border cells from approximately 12 o’clock to 7 o’clock as well as one cell at approximately 11 o’clock (Additional file 10Aii, Aii’). In larvae five to six dpfc *Lstag-sfc-9* was expressed in the elongated cells of the shell field margin. The signal was strong on the left side of the animal and decreased to the right side (Additional file 10Aiii, Aiii’). Larvae seven dpfc showed strong expression in the part of the mantle margin that lines the left and right side of the aperture (Additional file 10Aiv, Aiv’). Similar to *Lstag-sfc-6* to *8, Lstag-sfc-9* displayed a distinct and clear signal in zone three (Additional file 10Av, Av’).

Two transcripts are potential targets for the riboprobe that generated the expression pattern presented in Additional file 10A. The encoded proteins only vary by four amino acids (120 aa and 124 aa), are rich in asparagine (25%), glycine (24.2% and 25.8%) and tyrosine (18.3% and 19.4% respectively). Both proteins possess a signal sequence, a pI of 7.6 and are dominated by a repeated motif (Additional file 10B, C, Additional file 37).

***Lstag-sfc-10 (L. stagnalis-shell-forming candidate-10)***

In two to three dpfc old larvae, *Lstag-sfc-10* was expressed in a symmetrical ring within the cells bordering the shell gland (Additional file 11Ai, Ai’). Larvae three to six dpfc showed strong expression throughout the entire shell field margin (Additional file 11Aii, Aii’, Aiii, Aiii’). Larvae seven dpfc displayed a strong signal in the mantle margin lining the left and most of the right sides of the aperture (Additional file 11Aiv, Aiv’). Similar to *Lstag-sfc-6* to *9, Lstag-sfc-10* displayed a distinct and clear signal in zone three (Additional file 11Av, Av’).

Two transcripts are potential targets for the riboprobe that generated the expression pattern presented in Additional file 11A. The protein encoded by contig Lsta_ME_v2_idb_67296 is 330 aa long, has a signal sequence, a repetitive motif, five putative glycosylation sites and a theoretical pI of 5.76 (Additional file 11B, Additional file 37). The most abundant amino acids are glycine (19.7%), glutamine (9.1%), serine (9.1%) and proline (8.8%)(Additional file 37). Transcripts coding for similar proteins are present in other molluscs (Additional file 43). The protein encoded by contig Lsta_ME_v2_idb_67294 is 261 aa long, has no signal sequence, a theoretical pI of 8.46 and eight putative glycosylation sites; anomalous glycine (12.6%) and serine (11.1%) contents (Additional file 37). Transcripts coding for similar proteins are present in other molluscs (Additional file 43).

Contigs encoding *Lstag*-sfc-10 are present in mantle and larval transcriptomes and could be mapped to the draft genome (Additional file 11C). This gene apparently generates five different splice variants that encode four different proteins. Transcripts encoding the protein presented in Additional file 11B are present in the mantle and larval libraries. The mantle zone 1-5 transcriptome contains a contig that encodes a protein without a signal sequence (Additional file 37).

***Lstag-sfc-11 (L. stagnalis-shell-forming candidate-11)***

Larvae younger than five dpfc showed no expression of *Lstag-sfc-11* (Additional file 12Ai, Ai’, Aii, Aii’). A signal within the elongated cells of the shell field margin could be observed in larvae five to six dpfc (Additional file 12Aiii, Aiii’). Larvae seven dpfc showed expression in a broad ring along the mantle margin lining the left and right sides of the aperture (Additional file 12Aiv, iv’). A faint signal was detected in zone three in the adult mantle (Additional file 12Av, Av’). Five transcripts are potential binding sites for the riboprobe that generated the expression pattern presented in Additional file 12A.

The *Lstag*-sfc-11 protein encoded by contig Lsta_ME_v2_CLC_58 has a signal sequence and is potentially secreted (Additional file 12B). The *Lstag*-sfc-11 protein encoded by contigs Lsta_ME_v2_47522, _47520, _47525 and _47519 has no signal sequence (Additional file 37). All proteins derived from these contigs contain two repeated motifs, have eight to ten glycosylation sites and a theoretical pI between 4.52 and 5.09 and anomalous alanine (13.1-13.4%), threonine (11.9-13.4%), serine (9.2-11.4%) and leucine (8.4-9%) contents (Additional file 37).

Splice variants of *Lstag-sfc-11* are present exclusively in the adult mantle tissue and could be mapped to the draft genome and show great diversity (Additional file 12C). Overall, both mantle transcriptomes contains five different splice variants for *Lstag-sfc-11* that encode three different proteins (Additional file 12C). The encoded proteins show similar protein properties, a striking difference is the presence or absence of a signal sequence (Additional file 37). Notably, three splice variants with markedly diverging 5’ UTR generate identical proteins (Additional file 12C, Additional file 37).

***Lstag-sfc-12 (L. stagnalis-shell-forming candidate-12)***

No expression of *Lstag-sfc-12* was detected in larvae younger than five dpfc (Additional file 13Ai, Ai’, Aii, Aii’). Larvae five to six dpfc showed expression in the left side of the mantle (Additional file 13Aiii, Aiii’). Larvae older than seven dpfc displayed a signal in some parts of the mantle margin (Additional file 13Aiv, Aiv’). The adult mantle showed a faint signal in zone three (Additional file 13Av, Av’).

***Lstag-sfc-13 (L. stagnalis-shell-forming candidate-13)***

*Lstag-sfc-13* was expressed in a broad pattern throughout the shell forming tissue in larvae two to six dpfc (Additional file 14Ai-Aiii’, Ai’-Aiii’) and in a large rim across the mantle margin in larvae seven dpfc (Additional file 14Aiv, Aiv’). The adult mantle tissue exhibited a faint signal in zone three (Additional file 14Av, Av’).

The 117 aa long *Lstag*-sfc-13 protein has a signal sequence, a repeated motif, a theoretical pI of 6.88 and anomalous glycine (26.5%), tyrosine (11.1%) and phenylalanine (10.3%) contents (Additional file 14B, Additional file 37). *Lstag-sfc-13* is present only in the mantle transcriptome and could be mapped to the draft genome (Additional file 14C).

***Lstag-sfc-14 (L. stagnalis-shell-forming candidate-14)***

*Lstag-sfc-14* was expressed in the invaginated cells of the shell gland in larvae two to three dpfc (Additional file 15Ai, Ai’). A broad expression across the whole shell field was detected within larvae three to six dpfc (Additional file 15Aii, Aii’, Aiii, Aiii’). A signal was observed in the mantle edge that lines the left side of the aperture in larvae seven dpfc (Additional file 15Aiv, Aiv’). The adult mantle tissue displayed staining in zone three (Additional file 15Av, Av’).

The 305 aa long *Lstag*-sfc-14 protein has a signal sequence, a repeated motif, a theoretical pI of 9.83 and anomalous glycine (10.8%) and arginine (9.5%) contents (Additional file 15B, Additional file 37). Transcripts encoding proteins similar to Lstag-sfc-14 are apparently unique to the Mollusca (Additional file 43). Contigs encoding *Lstag-sfc-14* are present in the adult mantle tissue and larval transcriptomes and could be mapped to the draft genome (Additional file 15C). These contigs vary in their 3’ and 5’ UTR lengths, and all encode the protein presented in Additional file 15B.

***Lstag-sfc-15 (L. stagnalis-shell-forming candidate-15)***

No expression of *Lstag-sfc-15* could be detected in larvae younger than seven dpfc (Additional file 16Ai-Aiii, Ai’-Aiii’). In larvae seven dpfc expression of *Lstag-sfc-15* was observed within the mantle edge that lines the left side of the aperture (Additional file 16Aiv, Aiv’). The adult mantle showed expression throughout the whole belt (Additional file 16Av’, Av’).

The 134 aa long *Lstag*-sfc-15 protein has a signal sequence, a theoretical pI of 6.14, and anomalous leucine (10.4%), serine (9.7%) and alanine (9.0%) contents (Additional file 16B, Additional file 37). Contigs encoding *Lstag*-sfc-15 are present the mantle tissue transcriptomes as well as the cephalic tentacle, CNS and buccal mass transcriptomes (Additional file 16C). These contigs vary slightly in their 3’ and 5’ UTR lengths but all encode the protein presented in Additional file 16B.

***Lstag-sfc-16 (L. stagnalis-shell-forming candidate-16)***

Expression of *Lstag-sfc-16* could not be detected in any larval stages (Additional file 17Ai-Aiv, Ai’-Aiv’). The adult mantle showed strong expression throughout the whole belt (Additional file 17Av, Av’).

The 148 aa long *Lstag*-sfc-16 protein has a signal sequence, a theoretical pI of 4.69 and anomalous leucine (11.5%) threonine (9.5%), asparagine (8.8%) and aspartic acid (8.1%) contents (Additional file 17B, Additional file 37). The protein is predicted to be involved in calcium binding (Additional file 37). Contigs encoding proteins with similar sequences are apparently unique to the Mollusca (Additional file 43).

***Lstag-sfc-17 (L. stagnalis-shell-forming candidate-17)***

Expression of *Lstag-sfc-17* was detected on the left side of the shell forming tissue in all larval stages (Additional file 18Ai-iv, Ai’-iv’). Within larvae two to three dpfc *Lstag-sfc-17* was expressed in cells bordering the shell gland from approximately 9 – 12 o’clock (Additional file 18Ai, Ai’). Larvae three to six dpfc showed expression in the elongated border cells of the left half of the shell field (Additional file 18Aii, Aii’, Aiii, Aiii’). In larvae seven dpfc *Lstag-sfc-17* expression was present in the mantle margin lining the parietal region of the aperture (Additional file 18Aiv, Aiv’). The region of the adult mantle adjacent to the parietal region of the shell displayed *Lstag-sfc-17* signal in the mantle belt (Additional file 18Av, Av’).

The 116 aa long *Lstag*-sfc-16 protein has a signal sequence, a theoretical pI of 6.56 and anomalous proline (14.7%), valine (9.5%) and leucine (9.5%) contents (Additional file 18B, Additional file 37). *Lstag-sfc-17* is present in the adult mantle zone 1-5 and the 67 hpfc transcriptomes and could be mapped to the draft genome. Each of these transcriptomes contains contigs that encode the protein presented in Additional file 18B. The mantle zone 1-5 transcriptome contains a second splice variant, encoding a protein with a truncated C-terminus (Additional file 18C).

***Lstag-sfc-18 (L. stagnalis-shell-forming candidate-18)***

Larvae two to three dpfc showed expression of *Lstag-sfc-18* in cells of the foot, the apical plate and one cell beneath the shell gland (Additional file 19Ai, Ai’). Individuals three to four days showed expression within various parts of the larvae, including cells of the velum and the head as well as some cells beneath the shell field (Additional file 19Aii, Aii’). Larvae five to six dpfc displayed expression of *Lstag-sfc-18* in some cells of the head region and cells lining the shell field (Additional file 19Aiii, Aiii’). In larvae seven dpfc one cell at the most proximal part of the right side of the mantle margin showed a strong *Lstag-sfc-18* signal. Expression could also be monitored in some parts of the mantle edge that lines the anterior and left side of the aperture (Additional file 19Aiv, Aiv’). Adult mantle tissue showed expression along the inner low columnar epithelium and in cells of the belt (Additional file 19Av, Av’).

The 267 aa long *Lstag*-sfc-18 protein shows sequence similarities with the neural/ectodermal development factor IMP-L2-like (Additional file 37), 2 Ig domain protein zig-4 (Additional file 37) and two conserved Ig domains (Additional file 19B, Additional file 40). It has a signal sequence, a theoretical pI of 6.22 and the most abundant amino acid is serine (8.2%) (Additional file 37). Sequences similar to *Lstag*-sfc-18 are present in a broad range of metazoan phyla (Additional file 43).

Contigs encoding *Lstag*-sfc-18 are present in mantle tissue, cephalic lobe, CNS, foot, buccal mass and larval transcriptomes and could be mapped to the draft genome (Additional file 19C). Splice variants encode proteins that vary in their C-termini (Additional file 19C). The general protein properties and predicted molecular function are similar for all translated contigs (Additional file 37). Significant variation in the 3’ UTRs of these contigs can be observed (Additional file 19C). The CNS transcriptome contains the greatest splicing diversity of *Lstag-sfc-18* with three different splice variants (Additional file 19C).

***Lstag-sfc-19 (L. stagnalis-shell-forming candidate19)***

No distinct signal for *Lstag-sfc-19* could be detected in larval tissues (Additional file 20Ai-Aiv, Ai’-Aiv’). Adult mantle sections displayed a *Lstag-sfc-19* signal in zone four (Additional file 20Av, Av’).

The 195 aa long *Lstag*-sfc-19 protein has a signal sequence, an anomalous amino acid content of leucine (9.2%) and a theoretical pI of 4.98 (Additional file 20B, Additional file 37). It has a conserved C-type lectin (CTL)/C-type lectin-like (CTLD) domain and shows similarity to perlucin, ladderlectin and mannose-binding protein (Additional file 37). Transcripts encoding proteins with similar sequences are present in a broad range of metazoans (Additional file 43)

***Lstag-sfc-20 (L. stagnalis-shell-forming candidate-20)***

Expression of *Lstag-sfc-20* was detected in the invaginated cells of the shell gland (Additional file 21Ai, Ai’). A broad signal across the whole shell field was present within larvae three to six dpfc (Additional file 21Aii, Aii’, Aiii, Aiii’). In larvae seven dpfc a signal was observed within the mantle edge that lines the left side of the aperture (Additional file 21Aiv, Aiv’). The adult mantle displayed a faint signal in zone four (Additional file 21Av, Av’).

Two transcripts are potential targets for the riboprobe that generated the expression pattern presented in Additional file 21A but both of these encode very similar proteins. Both proteins have a signal sequence and the most abundant amino acid is leucine (9.7%) (Additional file 21B, C, Additional file 37). The encoded 243 aa long protein of contig Lsta_ME_v2_idb_71081 has a theoretical pI of 9.03, and the encoded 257 aa long protein of contig Lsta_ME_v2_idb_71083 has a theoretical pI of 9.23 (Additional file 37). Transcripts encoding proteins with sequences similar to *Lstag*-sfc-20 are apparently unique to the Mollusca (Additional file 43).

***Lstag-sfc-21 (L. stagnalis-shell-forming candidate-21)***

*Lstag-sfc-21* could not be detected in larvae younger than seven dpfc (Additional file 22Ai-Aiii, Ai’-Aiii’). Larvae seven dpfc displayed expression in the mantle margin that lines the left and right sides of the aperture (Additional file 22Aiv, Aiv’). The adult mantle tissue showed a signal in zones one, two and five with a conspicuous absence in zones three and four. This is a unique expression pattern that was not observed for any other candidate (Additional file 22Av, Av’).

The 442 aa long *Lstag*-sfc-21 protein has a signal sequence, a theoretical pI of 4.84, 12 putative glycosylation sites and the most abundant amino acid is threonine (8.8%) (Additional file 37). *Lstag*-sfc-21 contains a putative catalytic domain of chitin deacetylase-like proteins (Additional file 22B, Additional file 40). Transcripts encoding proteins with similar sequences are present in a broad range of metazoan phyla (Additional file 43).

Contigs encoding *Lstag*-sfc-21 are present in the transcriptomes of the mantle, cephalic tentacle, CNS and buccal mass and could be mapped to the draft genome (Additional file 22C), and all of these contigs encode the protein presented in Additional file 22B. Both mantle transcriptomes contain a diversity of splice variants, 6 in total (Additional file 22C). Five of the encoded proteins share similarity to the putative catalytic domain of chitin deacetylase-like proteins and are predicted to have a catalytic function (Additional file 37). One splice variant does not contain the putative catalytic domain and is not predicted to have a catalytic function (Additional file 37). Two candidates have a putative catalytic activity but are not predicted to be involved in chitin binding, as all other *Lstag*-sfc-21 variants (Additional file 37). Some of the encoded proteins do not carry a signal sequence (Additional file 37). The genomic origin of the 5’ and 3’ UTR varies across some contigs (Additional file 22C).

***Lstag-sfc-22 (L. stagnalis-shell-forming candidate-22)***

Expression of *Lstag-sfc-22* could not be detected in any larval stage (Additional file 23Ai-Aiv, Ai’-Aiv”). The adult mantle displayed *Lstag-sfc-22* expression within zone five (Additional file 23Av, Av’).

The 727 aa long *Lstag*-sfc-22 protein shares similarity with PIF and an asparagine-rich protein (despite *Lstag-*sfc-22 only containing 4.1% Asn) (Additional file 37), has a signal sequence, a theoretical pI of 5.08, 76 putative glycosylation sites, and an anomalous threonine content (13.1%)(Additional file 37). The *Lstag*-sfc-22 protein is predicted to be involved in chitin binding (Additional file 37). Transcripts encoding proteins with similar sequences are apparently unique to the Mollusca (Additional file 43).

Contigs encoding *Lstag*-sfc-22 are present in the mantle, cephalic tentacle, CNS and buccal mass transcriptomes and could be mapped to the draft genome (Additional file 23C). All transcriptomes contain a contig which represents the protein presented in Additional file 22B. The mantle zone 5 transcriptome contains a second truncated splice variant (Additional file 23C). This contig is missing several exons relative to the aforementioned, but the encoded protein does still carry the threonine rich motif and is predicted to bind chitin (Additional file 37).

***Lstag-sfc-23 (L. stagnalis-shell-forming candidate-23)***

Larvae two to three dpfc express *Lstag-sfc-23* strongly in the invaginated cells of the shell gland (Additional file 24Ai, Ai’). Larvae three to six dpfc showed a signal across the entire shell field (Additional file 24Aii, Aii’, Aiii, Aiii’). *Lstag-sfc-23* was expressed in mantle margin that lines the left side of the aperture in larvae seven dpfc (Additional file 24Aiv, Aiv’). The adult mantle displayed expression within zones three to five. This is a unique expression pattern in larvae and the adult mantle that was not observed for any other candidate (Additional file 24Av, Av’).

Two transcripts are potential targets for the riboprobe that generated the expression pattern presented in Additional file 24A. The protein encoded by contig Lsta_ME_v2_Tri_102017 is 1,536 aa long, has a signal sequence, a theoretical pI of 4.46 and anomalous aspartic acid (15.6%) and leucine (10.2%) contents (Additional file 37). The protein sequence contains two poly-D regions and two repeated motifs (Additional file 24B, Additional file 37) and shows similarity with otoancorin (Additional file 37). The protein has similarities with gene products present in a broad range of metazoan phyla (Additional file 43). The protein encoded by contig Lsta_ME_v2_Tri_102018 is 1,324 aa long, has a signal sequence, a theoretical pI of 4.62 and anomalous aspartic acid (13.7%) and leucine (11.0%) contents (Additional file 37). The protein contains a repeated motif and two poly-D regions (Additional file 24C, Additional file 37) and shows similarities to otoancorin and stereocilin (Additional file 37). The protein has similarities with gene products present in a broad range of metazoan phyla (Additional file 43).

***Lstag-sfc-24 (L. stagnalis-shell-forming candidate-24)***

Expression of *Lstag-sfc-24* was detected in invaginated cells of the shell gland, solely on the left side (Additional file 25Ai, Ai’). A broad expression across the whole shell field was detected in larvae three to six dpfc (Additional file 25Aii, Aii’, Aiii, Aiii’). After seven dpfc a signal could be detected in an area of the mantle margin that lines the left side of the aperture (Additional file 25Aiv, Aiv’). In adult mantle tissue, expression was observed in zone five (Additional file 25Av, Av’).

Two transcripts are potential targets for the riboprobe that generated the expression pattern presented in Additional file 25A. The protein encoded by contig Lsta_ME_v2_idb_51055 is 374 aa long, has a signal sequence and a repeated motif (Additional file 25B, Additional file 37). It has a theoretical pI of 4.74, 40 putative glycosylation sites and anomalous aspartic acid (20.6%), serine (12.6%) and asparagine (10.7%) contents (Additional file 37). The protein encoded by contig Lsta_ME_v2_idb_51053 is 430 aa long, has a repeated motif, a theoretical pI of 4.88, 42 putative glycosylation sites and anomalous aspartic acid (18.4%), serine (12.6%) and asparagine (10.7%) contents (Additional file 37.). Transcripts similar *Lsta*g-sfc-24 are apparently unique to molluscs (Additional file 43).

Contigs encoding *Lstag-sfc-24* are present in the mantle, CNS, and larval transcriptomes and could be mapped to the draft genome (Additional file 25C). Each transcriptome contains contigs encoding the protein presented in Additional file 25B. Notably, the mantle edge contains a great variety of splice variants; the encoded proteins vary in their C- and/or N- termini (Additional file 25C). All encoded proteins have an Asp-rich repeated motif, are predicted to carry a large number of glycosylation sites (between 39 and 44), and two of the encoded proteins have a signal sequence (Additional file 37).

***Lstag-sfc-25 (L. stagnalis-shell-forming candidate-25)***

*Lstag-sfc-25* could not be detected in larvae younger than seven dpfc (Additional file 26Ai-Aiii, Ai’-Aiii’). In larvae seven dpfc a signal was detected in the part of the mantle margin that lines the left and right side of the aperture (Additional file 26Aiv, Aiv’). The adult mantle displayed expression in zones four and five (Additional file 26Av, Av’).

The 242 aa long *Lstag*-sfc-25 protein has a signal sequence, a theoretical pI of 9.55, eight putative glycosylation sites, and anomalous alanine (12%), proline (8.3%) and serine (8.3%) contents (Additional file 26B, Additional file 37). Transcripts encoding proteins with similar sequences are apparently unique to the Mollusca (Additional file 43).

***Lstag-sfc-26 (L. stagnalis-shell-forming candidate-26)***

*Lstag-sfc-26* was expressed in the invaginated cells of the shell gland in larvae two to three dpfc (Additional file 27Ai, Ai’). A broad expression across the whole shell field was detected within larvae three to four dpfc (Additional file 27Aii, Aii’). *Lstag-sfc-26* was expressed within the elongated cells of the shell field margin in larvae five to six dpfc (Additional file 27Aiii, Aiii’) and within the mantle edge that lines the left side of the aperture in larvae seven dpfc (Additional file 27Aiv, Aiv’). The adult mantle showed a faint signal in zone five (Additional file 27Av, Av’).

The 329 aa long *Lstag*-sfc-26 protein has a signal sequence, eight putative glycosylation sites, a repeat motif, a theoretical pI of 8,82 and anomalous asparagine (9.7%), glycine (9.7%) and glutamine (8.8%) contents (Additional file 27B, Additional file 37). The protein shows similarity with a predicted uncharacterized *Biomphalaria glabrata* protein (Additional file 36 and 42). Transcripts encoding proteins with similar sequences are apparently unique to the Mollusca (Additional file 43).

***Lstag-sfc-27 (L. stagnalis-shell-forming candidate-27)***

*Lstag-sfc-27* was expressed in the invaginated cells of the shell gland in larvae two to three dpfc (Additional file 28Ai, Ai’). A broad expression across the whole shell field was detected in larvae three to six dpfc (Additional file 28Aii, Aii’, Aiii, Aiii’). A signal was observed in a broad rim in the mantle edge that lines the left side of the aperture in larvae seven dpfc (Additional file 28Aiv, Aiv’). The adult mantle displayed expression in zone five (Additional file 28Av, Av’).

Two transcripts are potential targets for the riboprobe that generated the expression pattern presented in Additional file 28A. Both proteins show sequence similarity with a predicted extensin-like protein and a predicted formin-like protein 2 (Additional file 42), have a signal sequence and a repeated motif (Additional file 28B, C) .The 224 aa long protein encoded by contig Lsta_ME_v2_idb_25973 has a predicted pI of 8.89, one putative glycosylation site and anomalous proline (18.8%), glycine (10.7%) and leucine (10.3%) contents (Additional file 37). The 182 aa long protein encoded by contig Lsta_ME_v2_idb_25972 has a theoretical pI of 7.01 and anomalous proline (19.2%) and glycine (11.5%) contents (Additional file 37). Transcripts encoding proteins with similar sequences to *Lstag*-sfc-27 are apparently unique to the Mollusca (Additional file 43).

***Lstag-sfc-28 (L. stagnalis-shell-forming candidate-28)***

*Lstag-sfc-28* could not be detected in larvae two to three dpfc (Additional file 29Ai, Ai’). In larvae three to six dpfc a faint broad signal across the entire shell field was observed (Additional file 29Aii, Aii’, Aiii, Aiii’). A signal in a broad area across the mantle edge, especially in the area that spans the most anterior parts of the margin, was detected in larvae seven dpfc (Additional file 29Aiv, Aiv’). In adult mantle tissue expression was observed in zone five (Additional file 29Av, Av’).

Three transcripts are potential binding sites for the riboprobe that generated the expression pattern presented in Additional file 29A. The 651 aa long protein encoded by contig Lsta_ME_v2_idb_25543 has a signal sequence, a striking 78 putative glycosylation sites, seven different repeated motifs, a theoretical pI of 8.77 (Additional file 29B, Additional file 37) and anomalous glutamine (20.4%), proline (14.7%) and alanine (11.5%) contents (Additional file 37). The 455 aa long protein encoded by contig Lsta_ME_v2_Tri_26754 has 48 putative glycosylation sites, a theoretical pI of 5.39 and anomalous glutamine (17.8%) and proline (12.2%) contents (Additional file 37). The 189 aa long protein encoded by contigs Lsta_ME_v2_idb_25544, Lsta_ME_v2_Tri_26753 and Lsta_ME_v2_CLC_8753 has 3 putative glycosylation sites, a theoretical pI of 5.37 and anomalous proline (12.2%), alanine (11.6%), leucine (11.1%), threonine (9.5%) and serine (9.0%) contents (Additional file 37).

***Lstag-sfc-29 (L. stagnalis-shell-forming candidate-29)***

Expression of *Lstag-sfc-29* could not be detected in larvae younger than seven dpfc (Additional file 30Ai-Aiii, Ai’-Aiii’). Larvae seven dpfc showed *Lstag-sfc-29* expression in a broad rim along the mantle edge lining the left side of the aperture (Additional file 30Aiv, Aiv’). In adult mantle tissue a strong signal was detected in zones four and five (Additional file 30Av, Av’).

The 381 aa long *Lstag*-sfc-29 protein shares similarity with galaxin (Additional file 37) and a predicted alpha protein kinase-like protein (Additional file 42). It has a signal sequence, a theoretical pI of 8.41, 12 putative glycosylation sites, a repeated motif and anomalous proline (18.6%) glutamine (10.8%) and glycine (8.1%) contents (Additional file 30B, Additional file 37). Transcripts encoding proteins with similar sequences are present in a broad range of metazoan taxa (Additional file 43).

***Lstag-sfc-30 (L. stagnalis-shell-forming candidate-30)***

No expression of *Lstag-sfc-30* was detected in larvae two to three dpfc (Additional file 31Ai, Ai’). A broad expression across the whole shell field was monitored in larvae three to six dpfc (Additional file 31Aii-Aiii, Aii’-Aiii’). Larvae seven dpfc showed a broad signal along the mantle edge (Additional file 31Aiv, Aiv’). The adult mantle showed a faint signal in zone five (Additional file 31Av, Av’). The incomplete protein has two putative glycosylation sites (Additional file 37).

***Lstag-sfc-31 (L. stagnalis-shell-forming candidate-31)***

*Lstag-sfc-31* could not be detected in larvae younger than seven dpfc (Additional file 32Ai-III, Ai’- Aiii’). A broad expression along the area of the mantle margin that lines the left side of the aperture could be observed in larvae seven dpfc (Additional file 32Aiv, Aiv’). The adult mantle showed a signal in all five shell-secreting zones (Additional file 32Av, Av’).

The 479 aa long *Lstag*-sfc-31 protein shares sequence similarity with sushi, von Willebrand factor type A, EGF and pentraxin domain-containing proteins and the complement receptor type 1 (Additional file 37). It has a sushi domain (Additional file 36 and 41), a signal sequence, six putative glycosylation sites, two C-terminal repeated motifs, a theoretical pI of 5.52 and anomalous glycine (14.0%), alanine (12.1%), leucine (9.6%) and proline (8.8%) contents (Additional file 31B, Additional file 37). Transcripts encoding proteins with similar sequences are present in a broad range of metazoan taxa (Additional file 43).

***Lstag-sfc-32 (L. stagnalis-shell-forming candidate-32)***

Cells of the velum, the apical plate, between the right and left half of the footlobe and surrounding the shell forming tissue displayed expression of *Lstag-sfc-32* within larvae two to four dpfc (Additional file 33Ai, Ai’, Aii, Aii’). Larvae five to six dpfc displayed signal along the head, the foot and surrounding the shell field (Additional file 33Aiii, Aiii’). The mantle margin along the left and right sides of the aperture, as well cells of the head and along the foot displayed staining in larvae seven dpfc (Additional file 33Aiv, Aiv’). Adult mantle tissue showed expression along the low columnar inner and outer epithelium as well as the mantle belt and low columnar outer epithelium (zones one to five)(Additional file 33Av, v’).

The 578 aa long *Lstag*-sfc-32 protein shows similarity to a retrograde protein and various filament proteins and is predicted to have structural molecule activity (Additional files 37, 41 and 42). An intermediate filament domain makes up the majority of the protein (Additional file 33, Additional file 40). It has a theoretical pI of 5.5 and anomalous leucine (11.2%), glutamic acid (10.7%), serine (8.3%) and alanine (8.5%) contents (Additional file 37). Transcripts encoding proteins with similar sequences are present in a broad range of metazoan phyla (Additional file 43).

*Lstag-sfc-32* contigs are present in mantle tissue, cephalic tentacle, cephalic lobe, CNS, buccal mass, foot and larval tissue transcriptomes and these could be mapped to the draft genome (Additional file 33C). The mantle, cephalic tentacle, cephalic lobe and foot transcriptomes all contain more than one splice variant related to *Lstag-sfc-32*. The splice variants presented in Additional file 33C encode three different proteins that all have similar sequence properties and are predicted to have a structural activity (Additional file 37). A significant variation between alternately spliced transcripts can be observed in the 3’ UTR (Additional file 33C).

***Lstag-sfc-33 (L. stagnalis-shell-forming candidate-33)***

Larvae younger than five dpfc showed no expression of *Lstag-sfc-33* (Additional file 34Ai, Ai’, Aii, Aii’). In larvae older than five to six dpfc a spotted pattern could be monitored within the foot, the head and along the mantle edge (Additional file 34Aiii, Aiii’, Aiv, Aiv”). Within adult mantle tissue, a spotted pattern along the most proximal part of the mantle along the inner epithelium was detected (Additional file 34Av, Av’).

The 466 aa long *Lstag*-sfc-33 protein shows similarity to various collagen alpha chain proteins, the transmembrane matrix receptor MUP-4 (Additional file 37) and a predicted cartilage matrix protein-like (Additional files 40 and 41). The von Willebrand factor A domain containing protein has a signal sequence, 11 putative glycosylation sites, a repeated motif, a theoretical pI of 7,52 and anomalous valine (8.4%) and alanine (8.2%) contents (Additional file 34B, Additional file 37). Transcripts encoding proteins with similar sequences are present in a broad range of metazoan phyla (Additional file 43).

***Lstag-sfc-34 (L. stagnalis-shell-forming candidate-34)***

Larvae two to three dpfc exhibited *Lstag-sfc-34* positive cells above the stomodeum (Additional file 35Ai, Ai’). Larvae three to four dpfc showed expression in cells above the stomodeum, and single cells on the left and right side of the velum, the foot lobe and a cell underneath the shell field (Additional file 35Aii, Aii’). Larvae five to six dpfc and seven dpfc showed a spotted pattern in the head, the foot and along the mantle margin (Additional file 35Aiii, Aiii’, Aiv, Aiv’). No expression could be detected within the adult mantle tissue (Additional file 35Av, Av’).

The 119 aa long *Lstag*-sfc-34 protein has a signal sequence, two putative glycosylation sites, a theoretical pI of 4.28 and anomalous glutamine (12.6%) and glycine (8.4%) contents (Additional file 35B, Additional file 37).

**Supplementary discussion**

Here we provide additional discussion for a variety of aspects arising from our dataset. These points are grouped into biologically themed topics.

**Chitin-binding shell-forming candidates**

Many proteins associated with chitin synthesis [1-3], degradation [3] and binding [4-7] have been identified in molluscan shell matrices. In nacre, chitin is known to be an integral part of the matrix scaffold [8] [9-14]. Recently, a number of chitinases potentially involved in chitin-remodelling have been identified in the mantle tissue of *L. stagnalis* [3]. We report here three shell-forming candidates (*Lstag-*sfc-4, -21 and -22) that are predicted to bind chitin (Additional file 37). *Lstag-*sfc-21 is characterised by a putative catalytic domain of chitin deacetylase-like proteins (Additional file 37) that might be able to modify chitin to chitosan [15, 16]. *Lstag-sfc-21* has a very distinct spatial expression pattern in the adult mantle, and is the only gene we characterised to display expression in zones one, two and five but not in zones three and four (Additional file 22). Another putative chitin-binding candidate is the Pif-like *Lstag-*sfc-22 (Additional file 23). Pif is a protein that was first identified in the oyster *Pinctada fucata* and was reported to induce aragonite crystal formation *in vitro* and to influence the formation of nacre *in vivo* [4]. However the function of the Pif-like *Lstag-*sfc-22 remains to be investigated [17].

**Aspartic acid-rich shell-forming candidates**

Aspartic acid-rich proteins are known to be a major component of the soluble organic matrix in a broad range of molluscan taxa [18]. *In vitro*, they can induce and inhibit crystal growth [19]. It is thought that these acidic macromolecules adsorb to the chitin framework, and provide an organic template for epitaxial crystal growth [19, 20]. We detected two shell-forming candidates (*Lstag-sfc-23* and *-24*) that code for aspartic acid-rich proteins and are expressed in the mantle of *L. stagnalis*. Strikingly, both of these shell-forming candidates are expressed in a broad pattern throughout the shell field during development, and also in zone 5 in the adult mantle (Additional files 24 and 25 respectively). What the role of these candidates is remains unknown, however they are likely to have functions distinct from candidates that are expressed in cells that border the shell gland/field in larvae or in adult mantle zones 1-4.

**Glycosylated shell-forming candidates**

Glycosylation is a common feature of molluscan shell matrix proteins. These prosthetic groups are generally carboxylated or sulphated [21] allowing them to enhance biomineral nucleation [22]. In cartilage, proteins linked with sulphated polysaccharides are involved in the osmotic equilibrium of the tissue through cation concentration [23]. Similar effects have been postulated for the nucleation of CaCO_3_, where calcium ions might be concentrated by the sulphate groups and directed to the fixed carboxylate positions of aspartic acid-rich proteins [21, 23, 24]. Due to the variety of possible sugar residue modifications, there exists an enormous molecular diversity of glycosylated proteins that are often branched and able to form various covalent linkages [25, 26]. Several shell-forming candidates that are expressed in zone five in the adult *L. stagnalis* mantle are predicted to be extensively glycosylated. For example, the secreted variant of *Lstag-*sfc-28 and the PIF-like *Lstag-*sfc-22 each possess more than 70 potential glycosylation sites (Additional file 37), while the putative chitin binding *Lstag-*sfc-21, the aspartic acid-rich *Lstag-*sfc-24, the galaxin-like *Lstag-*sfc-29 and the von Willebrand factor type A containing *Lstag*-sfc-33 are also predicted to have >10 polysaccharide attachment sites (Additional file 37). All of these candidates are expressed to various degrees in zone 5 of the adult mantle, and many of them also display broad expression throughout the shell field. Interestingly our preliminary glycosylation analysis of proteins extracted from the AIM and ASM fractions of *Lymanea*'s shell suggest that both the variety and abundances of monosaccharides are not particularly high (Table 1). While we cannot provide functional data for any of these putatively glycosylated shell-forming candidates, their shared biochemical features and spatial expression patterns hint at coordinated functions and/or sites of activity within the calcified shell. This data will also inform the design of experiments (and their interpretation) once gene specific functional assays become available for *L. stagnalis*.

**Basic shell-forming candidates**

Zone five of the adult mantle also contains many transcripts coding for shell-forming proteins with predicted high pI values. *Lstag-sfc-25*, *-26*, *-27*, *-28* and *-29* all encode proteins with theoretical pI values above 8.4 (Additional file 37). *Lstag*-sfc-27, -28 and -29 share proline and glutamine-rich repeated motifs, with glycine and alanine also being abundant (Additional file 37). Glycine, alanine and proline-rich domains have been suggested to contribute to the structural organic framework upon which molluscan shell formation proceeds [27], and proline-rich repeats have been reported in other molluscan shell proteins [28-30]. Proline introduces kinks and bends to the protein backbone, and sequences that contain high frequencies of proline, glutamine and alanine often adopt a polyproline helix that can promote interactions with other surfaces [31]. *Lstag*-sfc-27 (19% proline) shares sequence similarity with a predicted extensin-like protein (Additional file 42). These cell-wall proteins are rich in hydroxyprolines and are able to form cross-linked networks [32]. Extracellular matrices are often stabilized by cross-links to help maintain their structure under extracellular conditions [26].

**Shell-forming candidates expressed in the transitional zone between the belt and the low columnar epithelium**

Shell-forming candidates *Lstag-sfc-19* and *-20* are expressed in zone 4 that lies between the belt proper (zones 2 and 3) and the low columnar outer epithelium of zone 5 (Additional files 20 and 21 respectively). *Lstag*-sfc-19 contains a C-type lectin like domain (Additional file 36). These domains are known to be present in proteins in a variety of calcifying systems. In molluscs, the C-type lectin domain present in perlucin was shown to bind lactose and mannose and enhances the precipitation of CaCO_3_ *in vitro* [33, 34] [35], however its specific function *in vivo* is not known. It is possible that the rich diversity of glycosylated proteins present within the organic matrix of the shell (see above) would contain a ligand for this kind of calcium dependent lectin.

**Shell-forming candidates expressed in all zones of the mantle**

The sushi domain-containing candidate *Lstag*-sfc-31 and the intermediate filament protein *Lstag*-sfc-32 were detected throughout the mantle belt and the low columnar outer epithelium (Additional files 32 and 33 respectively). The sushi domain has been reported from a number of molluscan shell proteomes [36-39], is present in complement and adhesion proteins, and is known to be involved in protein-protein and protein-ligand interactions [40-42], however their role in shell formation remains elusive.

Filament proteins such as *Lstag*-sfc-32 have been detected in the shell proteomes of *H. aspersa*, *C.* *nemoralis* and *L. gigantea* [38, 43] [37]. Given their well-defined intra-cellular functions and lack of a canonical signal sequence, the presence of intracellular filament proteins in the molluscan shell could perhaps be attributed to the non-specific occlusion of mantle cells and cellular debris into a rapidly growing biomineral. Here we detected specific spatial expression of *Lstag*-sfc-32 in larval and adult shell-forming cells (Additional file 33). Filament proteins exhibit extreme extensibility and fracture resistance properties [44] which led Mann and Jackson [38, 44]) to propose a model in which intermediate filament rich cells are integrated into the growing shell in order to enhance its fracture resistance.

**Figure legends**

**Additional file 2. Spatial expression and molecular features of *Lstag-sfc-1*.** (A) *In situ* hybridisation against *Lstag-sfc-1* during development and in the fully differentiated adult mantle. The blue staining indicates the expression in the periphery of the shell forming tissue in larvae (i-iv, i’-iv’) and in zone one and two of the adult mantle (v, v’). Each column represents one developmental stage and shows two perspectives. The developmental stage is indicated in the header. The perspective is indicated in the lower right corner. v’ shows a magnification of the boxed section in v. Days post first cleavage (dpfc), lateral (lat), dorsal (dors), ventral (ventr). (B) Protein sequence and schematic representation of the translated Mantle_Edge_v2_Tri_252293. The signal sequence (red) and the peptides identified with MS/MS (boxed sections) are indicated.

**Additional file 3. Spatial expression and molecular features of *Lstag-sfc-2*.** (A) *In situ* hybridisation against *Lstag-sfc-2* during development and in the fully differentiated adult mantle. The blue staining indicates the expression in the periphery of the shell forming tissue in larvae (ii-iv, ii’-iv’) and zone one and two of the adult mantle (v, v’). Each column represents one developmental stage and shows two perspectives. The developmental stage is indicated in the header. The perspective is indicated in the lower right corner. v’ shows a magnification of the boxed section in v. Days post first cleavage (dpfc), lateral (lat.), dorsal (dors.), ventral (ventr.). (B) Protein sequence and schematic representation of the translated Mantle_Edge_v2_idb_77206. The signal sequence (red), the repeated motif (blue) and the peptides identified with MS/MS (boxed sections) are indicated.

**Additional file 4. Spatial expression and molecular features of *Lstag-sfc-3*.** (A) *In situ* hybridisation against *Lstag-sfc-3* during development and in the fully differentiated adult mantle. The blue staining indicates the expression in the periphery of the shell forming tissue in larvae (iii, iii’, iv, iv’) and zone one and two of the adult mantle (v, v’). Each column represents one developmental stage and shows two perspectives. The developmental stage is indicated in the header. The perspective is indicated in the lower right corner. v’ shows a magnification of the boxed section in v. Days post first cleavage (dpfc), lateral (lat.), dorsal (dors.), ventral (ventr.). (B) Protein sequence and schematic representation of the translated Edge_v2_CLC_107. The signal sequence (red), the repeated motifs (blue), and the peptide identified with MS/MS (boxed section) are indicated. (C) Schematic representation of the gene architecture and splice variation of *Lstag-sfc-3.* Transcriptomic contigs similar to Mantle_Edge_v2_CLC_107 were obtained from various *L. stagnalis* transcriptomes and aligned to the corresponding genomic contig gLs.1.0.scaf01097. The genomic contig is indicated by the horizontal black line, the aligned transcripts are indicated by the boxed sections, white boxes represent UTR region, blue boxes represent coding region. Scale bar represents 1000 bp.

**Additional file 5. Spatial expression and molecular features of *Lstag-sfc*-*4*.** (A) *In situ* hybridisation against *Lstag-sfc-4* during development and in the fully differentiated adult mantle.. The blue staining indicates the expression in the periphery of the shell forming tissue in larvae (iii, iv, iii’, iv’) and zone one at the posterior wall of the mantle groove (v, v’). Each column represents one developmental stage and shows two perspectives. The developmental stage is indicated in the header. The perspective is indicated in the lower right corner. v’ shows a magnification of the boxed section in v. Days post first cleavage (dpfc), lateral (lat.), dorsal (dors.), ventral (ventr.). (B) Protein sequence and schematic representation of the translated Mantle_Edge_v2_Tri_255684 and Mantle_Edge_v2_idb_73304. The signal sequence (red) and the peptide identified with MS/MS (boxed section) are indicated. (C) Schematic representation of the gene architecture and splice variation of the *Lstag-sfc-4*. Transcriptomic contigs similar to Mantle_Edge_v2_Tri_255684 and Mantle_Edge_v2_idb_73304. were obtained from various *L. stagnalis* transcriptomes and aligned to the corresponding genomic gLs.1.0.scaf01511. The genomic contig is indicated by the horizontal black line, the aligned transcripts are indicated by the boxed sections, white boxes represent UTR region, blue boxes represent coding region. Scale bar represents 1000 bp.

**Additional file 6. Spatial expression and molecular features of *Lstag-sfc-5*.** (A) *In situ* hybridisation against *Lstag-sfc-5* during development and in the fully differentiated adult mantle. The blue staining indicates the expression in the periphery of the shell forming tissue in larvae (i-iv, i’-iv’) and zone one and two of the adult mantle (v, v’). Each column represents one developmental stage and shows two perspectives. The developmental stage is indicated in the header. The perspective is indicated in the lower right corner. v’ shows a magnification of the boxed section in v. Days post first cleavage (dpfc), lateral (lat.), dorsal (dors.), ventral (ventr.). (B) Protein sequence and schematic representation of the translated Mantle_Edge_v2_CLC_241. The signal sequence (red), the conserved animal haem peroxidase domain (underlined black and highlighted grey) and the peptide identified with MS/MS (boxed section) are indicated.

**Additional file 7. Spatial expression and molecular features of *Lstag-sfc-6*.** (A) *In situ* hybridisation against *Lstag-sfc-6* during development and in the fully differentiated adult mantle. The blue staining indicates the expression in the periphery of the shell forming tissue in larvae (i-iv, i’-iv’) and zone three of the adult mantle (v, v’). Each column represents one developmental stage and shows two perspectives. The developmental stage is indicated in the header. The perspective is indicated in the lower right corner. v’ shows a magnification of the boxed section in v. Days post first cleavage (dpfc), lateral (lat.), dorsal (dors.), ventral (ventr.). (B) Protein sequence and schematic representation of the translated Mantle_Edge_v2_Tri_232353. The signal sequence (red), the repeated motif (blue) and the peptide identified with MS/MS (boxed section) are indicated. (C) Protein sequence and schematic representation of the translated Mantle_Edge_v2_idb_111343. The signal sequence (red), the repeated motif (blue) and the peptide identified with MS/MS (boxed section) are indicated.

**Additional file 8. Spatial expression and molecular features of *Lstag-sfc*-7.** (A) *In situ* hybridisation against *Lstag-sfc-7* during development and in the fully differentiated adult mantle. The blue staining indicates the expression in the periphery of the shell forming tissue in larvae (i-iv, i’-iv’) and zone three of the adult mantle (v, v’). Each column represents one developmental stage and shows two perspectives. The developmental stage is indicated in the header. The perspective is indicated in the lower right corner. v’ shows a magnification of the boxed section in v. Days post first cleavage (dpfc), lateral (lat.), dorsal (dors.), ventral (ventr.). (B) Protein sequence and schematic representation of the translated Mantle_Edge_v2_Tri_209370 and Mantle_Edge_v2_idb_133123. The signal sequence (red), the repeated motif (blue) are indicated.

**Additional file 9. Spatial expression and molecular features of *Lstag-sfc-8*.** (A) *In situ* hybridisation against *Lstag-sfc-8* during development and in the fully differentiated adult mantle. The blue staining indicates the expression in the periphery of the shell forming tissue in larvae (iv, iv’) and zone three of the adult mantle (v, v’). Each column represents one developmental stage and shows two perspectives. The developmental stage is indicated in the header. The perspective is indicated in the lower right corner. v’ shows a magnification of the boxed section in v. Days post first cleavage (dpfc), lateral (lat.), dorsal (dors.), ventral (ventr.). (B) Protein sequence and schematic representation of the translated Mantle_Edge_v2_Tri_126421. The signal sequence (red) and the repeated motif (blue) are indicated. (D) Schematic representation of the gene architecture and splice variation of *Lstag-sfc-8*. Transcriptomic contigs similar to Mantle_Edge_v2_Tri_126421 were obtained from various *L. stagnalis* transcriptomes and aligned to the corresponding genomic contig gLs.1.0.scaf03406. The genomic contig is indicated by the horizontal black line, the aligned transcripts are indicated by the boxed sections, white boxes represent UTR region, blue boxes represent coding region. Scale bar represents 1000 bp.

**Additional file 10. Spatial expression and molecular features of *Lstag-sfc-9*.** (A) *In situ* hybridisation against *Lstag-sfc-9* during development and in the fully differentiated adult mantle. The blue staining indicates the expression in the periphery of the shell forming tissue in larvae (i-iv, i’-iv’) and zone three of the adult mantle (v, v’). Each column represents one developmental stage and shows two perspectives. The developmental stage is indicated in the header. The perspective is indicated in the lower right corner. v’ shows a magnification of the boxed section in v. Days post first cleavage (dpfc), lateral (lat.), dorsal (dors.), ventral (ventr.). (B) Protein sequence and schematic representation of the translated Mantle_Edge_v2_idb_86937. The signal sequence (red) and the repeated motif (blue) are indicated. (C) Protein sequence and schematic representation of the translated Mantle_Edge_v2_idb_86933. The signal sequence (red) and the repeated motif (blue) are indicated.

**Additional file 11. Spatial expression and molecular features of *Lstag-sfc-10*.** (A) *In situ* hybridisation against *Lstag-sfc-10* during development and in the fully differentiated adult mantle. The blue staining indicates the expression in the periphery of the shell forming tissue in larvae (i-iv, i’-iv’) and zone three of the adult mantle (v, v’). Each column represents one developmental stage and shows two perspectives. The developmental stage is indicated in the header. The perspective is indicated in the lower right corner. v’ shows a magnification of the boxed section in v. Days post first cleavage (dpfc), lateral (lat.), dorsal (dors.), ventral (ventr.). (B) Protein sequence and schematic representation of the translated Mantle_Edge_v2_idb_67296. The signal sequence (red), the repeated motif (blue) and the peptides identified with MS/MS (boxed section) are indicated. (C) Schematic representation of the gene architecture and splice variation of the *Lstag-sfc-10*. Transcriptomic contigs similar to Mantle_Edge_v2_idb_67296 were obtained from various *L. stagnalis* transcriptomes and aligned to the corresponding genomic contig gLs.1.0.scaf01041. The genomic contig is indicated by the horizontal black line, the aligned transcripts are indicated by the boxed sections, white boxes represent UTR region, blue boxes represent coding region. Scale bar represents 1000 bp.

**Additional file 12. Spatial expression and molecular features of *Lstag-sfc-11*.** (A) *In situ* hybridisation against *Lstag-sfc-11* during development and in the fully differentiated adult mantle. The blue staining indicates the expression in the periphery of the shell forming tissue in larvae (iii, iv, iii’, iv’) and zone three of the adult mantle (v, v’). Each column represents one developmental stage and shows two perspectives. The developmental stage is indicated in the header. The perspective is indicated in the lower right corner. v’ shows a magnification of the boxed section in v. Days post first cleavage (dpfc), lateral (lat.), dorsal (dors.), ventral (ventr.). (B) Protein sequence and schematic representation of the translated Mantle_Edge_v2_CLC_58. The signal sequence (red), the repeated motifs (blue), C-terminal cysteine residues (highlighted grey) and the peptide identified with MS/MS (boxed section) are indicated. (C) Schematic representation of the gene architecture and splice variation of *Lstag-sfc-11*. Transcriptomic contigs similar to Mantle_Edge_v2_CLC_58 were obtained from various *L. stagnalis* transcriptomes and aligned to the corresponding genomic contig gLs.1.0.scaf01302. The genomic contig is indicated by the horizontal black line, the aligned transcripts are indicated by the boxed sections, white boxes represent UTR region, blue boxes represent coding region. Scale bar represents 1000 bp.

**Additional file 13. Spatial expression and molecular features of *Lstag-sfc-12*.** (A) *In situ* hybridisation against *Lstag-sfc-12* during development and in the fully differentiated adult mantle. The blue staining indicates the expression in the periphery of the shell forming tissue in larvae (iii, iv, iii’, iv’) and zone three of the adult mantle (v, v’). Each column represents one developmental stage and shows two perspectives. The developmental stage is indicated in the header. The perspective is indicated in the lower right corner. v’ shows a magnification of the boxed section in v. Days post first cleavage (dpfc), lateral (lat.), dorsal (dors.), ventral (ventr.). (B) Incomplete protein sequence of the translated Mantle_Edge_v2_idb_103149. The peptide identified with MS/MS (boxed section) is indicated.

**Additional file 14. Spatial expression and molecular features of *Lstag-sfc-13*.** (A) *In situ* hybridisation against *Lstag-sfc-13* during development and in the fully differentiated adult mantle. The blue staining indicates the expression across the shell forming tissue in larvae (i-iv, i’-iv’) and zone three of the adult mantle (v, v’). Each column represents one developmental stage and shows two perspectives. The developmental stage is indicated in the header. The perspective is indicated in the lower right corner. v’ shows a magnification of the boxed section in v. Days post first cleavage (dpfc), lateral (lat.), dorsal (dors.), ventral (ventr.). (B) Protein sequence and schematic representation of the translated Mantle_Proximal_v2_idb_67016. The signal sequence (red), the repeated motif (blue) and the peptide identified with MS/MS (boxed section) are indicated. (C) Schematic representation of the gene architecture and splice variation of *Lstag-sfc-13*. Transcriptomic contigs similar to Mantle_Proximal_v2_idb_67016 were obtained from various *L. stagnalis* transcriptomes and aligned to the corresponding genomic contig gLs.1.0.scaf00172. The genomic contig is indicated by the horizontal black line, the aligned transcripts are indicated by the boxed sections, white boxes represent UTR region, blue boxes represent coding region. Scale bar represents 1000 bp.

**Additional file 15. Spatial expression and molecular features of *Lstag-sfc-14*.** (A) *In situ* hybridisation against *Lstag-sfc-14* during development and in the fully differentiated adult mantle. The blue staining indicates the expression across the shell forming tissue in larvae (i-iv, i’-iv’) and zone three of the adult mantle (v, v’). Each column represents one developmental stage and shows two perspectives. The developmental stage is indicated in the header. The perspective is indicated in the lower right corner. v’ shows a magnification of the boxed section in v. Days post first cleavage (dpfc), lateral (lat.), dorsal (dors.), ventral (ventr.). (B) Protein sequence and schematic representation of the translated Mantle_Edge_v2_Tri_28903. The signal sequence (red), the repeated motif (blue) and the peptide identified with MS/MS (boxed section) are indicated. (C) Schematic representation of the gene architecture and splice variation of *Lstag-sfc-14*. Transcriptomic contigs similar to Mantle_Edge_v2_Tri_28903 were obtained from various *L. stagnalis* transcriptomes and aligned to the corresponding genomic contig gLs.1.0.scaf02426. The genomic contig is indicated by the horizontal black line, the aligned transcripts are indicated by the boxed sections, white boxes represent UTR region, blue boxes represent coding region. Scale bar represents 1000 bp.

**Additional file 16. Spatial expression and molecular features of *Lstag-sfc-15*.** (A) *In situ* hybridisation against *Lstag-sfc-15* during development and in the fully differentiated adult mantle. The blue staining indicates the expression in the periphery of the shell forming tissue in larvae (iv, iv’) and the adult mantle belt (v, v’). Each column represents one developmental stage and shows two perspectives. The developmental stage is indicated in the header. The perspective is indicated in the lower right corner. v’ shows a magnification of the boxed section in v. Days post first cleavage (dpfc), lateral (lat.), dorsal (dors.), ventral (ventr.). (B) Protein sequence and schematic representation of the translated Mantle_Edge_v2_CLC_8979. The signal sequence (red) and the peptide identified with MS/MS (boxed section) are highlighted. (ç) Schematic representation of the gene architecture and splice variation of *Lstag-sfc-15*. Transcriptomic contigs similar to Mantle_Edge_v2_CLC_8979 were obtained from various *L. stagnalis* transcriptomes and aligned to the corresponding genomic contig gLs.1.0.scaf01427. The genomic contig is indicated by the horizontal black line, the aligned transcripts are indicated by the boxed sections, white boxes represent UTR region, blue boxes represent coding region. Scale bar represents 1000 bp.

**Additional file 17. Spatial expression and molecular features of *Lstag-sfc-16*.** (A) *In situ* hybridisation against *Lstag-sfc-16* during development and in the fully differentiated adult mantle. The blue staining indicates the expression in the adult mantle belt (v, v’). Each column represents one developmental stage and shows two perspectives. The developmental stage is indicated in the header. The perspective is indicated in the lower right corner. v’ shows a magnification of the boxed section in v. Days post first cleavage (dpfc), lateral (lat.), dorsal (dors.), ventral (ventr.). (B) Protein sequence and schematic representation of the translated CNS_v2_Tri_113901. The signal sequence (red) and the peptide identified with MS/MS (boxed section) are indicated.

**Additional file 18. Spatial expression and molecular features of *Lstag-sfc-17*.** (A) *In situ* hybridisation against *Lstag-sfc-17* during development and in the fully differentiated adult mantle. The blue staining indicates the expression in the periphery of the shell forming tissue in larvae (i-iv, i’-iv’) and zone four of the adult mantle (v, v’). (v, v’). Each column represents one developmental stage and shows two perspectives. The developmental stage is indicated in the header. The perspective is indicated in the lower right corner. v’ shows a magnification of the boxed section in v. Days post first cleavage (dpfc), lateral (lat.), dorsal (dors.), ventral (ventr.). (B) Protein sequence and schematic representation of the translated Mantle_Edge_v2_idb_90504. The signal sequence (red) and the peptide identified with MS/MS (boxed section) are highlighted. (C) Schematic representation of the gene architecture and splice variation of *Lstag-sfc-17*. Transcriptomic contigs similar to _Mantle_Edge_v2_idb_90504 were obtained from various *L. stagnalis* transcriptomes and aligned to the corresponding genomic contig gLs.1.0.scaf01608. The genomic contig is indicated by the horizontal black line, the aligned transcripts are indicated by the boxed sections, white boxes represent UTR region, blue boxes represent coding region. Scale bar represents 1000 bp.

**Additional file 19. Spatial expression and molecular features of *Lstag-sfc-18*.** (A) *In situ* hybridisation against *Lstag-sfc-18* during development and in the fully differentiated adult mantle. The blue staining indicates the expression in larvae (i-iv, i’-iv’) and zones two, three and four of the adult mantle (v, v’). Each column represents one developmental stage and shows two perspectives. The developmental stage is indicated in the header. The perspective is indicated in the lower right corner. v’ shows a magnification of the boxed section in v. Days post first cleavage (dpfc), lateral (lat.), dorsal (dors.), ventral (ventr.). (B) Protein sequence and schematic representation of the translated Mantle_Edge_v2_idb_32853. The signal sequence (red), the two conserved immunoglobulin domains (underlined black and highlighted grey) and the peptides identified with MS/MS (boxed sections) are indicated. (C) Schematic representation of the gene architecture and splice variation of the *Lstag-sfc-18*. Transcriptomic contigs similar to Mantle_Edge_v2_idb_32853 were obtained from various *L. stagnalis* transcriptomes and aligned to the corresponding genomic contig gLs.1.0.scaf01043. The genomic contig is indicated by the horizontal black line, the aligned transcripts are indicated by the boxed sections, white boxes represent UTR region, blue boxes represent coding region. Scale bar represents 1000 bp.

**Additional file 20. Spatial expression and molecular features of *Lstag-sfc-19*.** (A) *In situ* hybridisation against *Lstag-sfc-19* during development and in the fully differentiated adult mantle. We interpret the staining in juvenile animal to be non-specific background. The blue staining indicates the expression in zone four of the adult mantle (v, v’). Each column represents one developmental stage and shows two perspectives. The developmental stage is indicated in the header. The perspective is indicated in the lower right corner. v’ shows a magnification of the boxed section in v. Days post first cleavage (dpfc), lateral (lat.), dorsal (dors.), ventral (ventr.). (B) Protein sequence and schematic representation of the translated Mantle_Edge_v2_idb_42787. The signal sequence (red), the conserved C-type lectin/C-type lectin-like domain (underlined black and highlighted grey) and the peptide identified with MS/MS (boxed section) are indicated.

**Additional file 21. Spatial expression and molecular features of *Lstag-sfc-20*.** (A) *In situ* hybridisation against *Lstag-sfc-20* during development and in the fully differentiated adult mantle. The blue staining indicates the expression across the shell forming tissue in larvae (i-iv, i’-iv’) and zone four of the adult mantle (v, v’). Each column represents one developmental stage and shows two perspectives. The developmental stage is indicated in the header. The perspective is indicated in the lower right corner. v’ shows a magnification of the boxed section in v. Days post first cleavage (dpfc), lateral (lat.), dorsal (dors.), ventral (ventr.). (B) Protein sequence and schematic representation of the translated Mantle_Edge_v2_idb_71081. The signal sequence (red) and the peptides identified with MS/MS (boxed sections) are highlighted. (C) Protein sequence and schematic representation of the translated Mantle_Edge_v2_idb_71083. The signal sequence (red) and the peptides identified with MS/MS (boxed sections) are indicated.

**Additional file 22. Spatial expression and molecular features of *Lstag-sfc-21*.** (A) *In situ* hybridisation against *Lstag-sfc-21* during development and in the fully differentiated adult mantle. The blue staining indicates the expression in the larval mantle edge (iv, iv’) and zone one, two and five of the adult mantle (v, v’). Each column represents one developmental stage and shows two perspectives. The developmental stage is indicated in the header. The perspective is indicated in the lower right corner. v’ shows a magnification of the boxed section in v. Days post first cleavage (dpfc), lateral (lat.), dorsal (dors.), ventral (ventr.). (B) Protein sequence and schematic representation of the translated Mantle_Edge_v2_idb_97925. The signal sequence (red), the putative catalytic domain of chitin deacetylase-like proteins (underlined black and highlighted grey) and the peptide identified with MS/MS (boxed section) are indicated. (C) Schematic representation of the gene architecture and splice variation of *Lstag-sfc-21*. Transcriptomic contigs similar to Mantle_Edge_v2_idb_97925 were obtained from various *L. stagnalis* transcriptomes and aligned to the corresponding genomic contig gLs.1.0.scaf00440. The genomic contig is indicated by the horizontal black line, the aligned transcripts are indicated by the boxed sections, white boxes represent UTR region, blue boxes represent coding region. Scale bar represents 1000 bp.

**Additional file 23. Spatial expression and molecular features of *Lstag-sfc-22*.** (A) *In situ* hybridisation against *Lstag-sfc-22* during development and in the fully differentiated adult mantle. The blue staining indicates the expression in zone five of the adult mantle (v, v’). Each column represents one developmental stage and shows two perspectives. The developmental stage is indicated in the header. The perspective is indicated in the lower right corner. v’ shows a magnification of the boxed section in v. Days post first cleavage (dpfc), lateral (lat.), dorsal (dors.), ventral (ventr.). (B) Protein sequence and schematic representation of the translated Mantle_Edge_v2_Tri_283967. The signal sequence (red), the repeated motif (blue) and the peptides identified with MS/MS (boxed sections) are indicated. (C) Schematic representation of the gene architecture and splice variation of *Lstag-sfc-22*. Transcriptomic contigs similar to Mantle_Edge_v2_Tri_283967 were obtained from various *L. stagnalis* transcriptomes and aligned to the corresponding genomic contig gLs.1.0.scaf01583. The genomic contig is indicated by the horizontal black line, the aligned transcripts are indicated by the boxed sections, white boxes represent UTR region, blue boxes represent coding region. Scale bar represents 1000 bp.

**Additional file 24. Spatial expression and molecular features of *Lstag-sfc-23*.** (A) *In situ* hybridisation against *Lstag-sfc-23* during development and in the fully differentiated adult mantle. The blue staining indicates the expression across the shell forming tissue in larvae (i-iv, i’-iv’) and zones three, four and five of the adult mantle (v, v’). Each column represents one developmental stage and shows two perspectives. The developmental stage is indicated in the header. The perspective is indicated in the lower right corner. v’ shows a magnification of the boxed section in v. Days post first cleavage (dpfc), lateral (lat.), dorsal (dors.), ventral (ventr.). (B) Protein sequence and schematic representation of the translated Mantle_Edge_v2_Tri_102017. The signal sequence (red), the repeated motifs (blue) and the peptides identified with MS/MS (boxed sections) are indicated. (C) Protein sequence and schematic representation of the translated Mantle_Edge_v2_Tri_102018. The signal sequence (red), the repeated motifs (blue) and the peptides identified with MS/MS (boxed sections) are indicated.

**Additional file 25. Spatial expression and molecular features of *Lstag-sfc-24*.** (A) *In situ* hybridisation against *Lstag-sfc-24* during development and in the fully differentiated adult mantle. The blue staining indicates the expression across the shell forming tissue in larvae (i-iv, i’-iv’) and zone five of the adult mantle (v, v’). Each column represents one developmental stage and shows two perspectives. The developmental stage is indicated in the header. The perspective is indicated in the lower right corner. v’ shows a magnification of the boxed section in v. Days post first cleavage (dpfc), lateral (lat.), dorsal (dors.), ventral (ventr.). (B) Protein sequence and schematic representation of the translated Mantle_Edge_v2_idb_51055. The signal sequence (red), the repeated motif (blue) and the peptides identified with MS/MS (boxed sections) are indicated. (C) Schematic representation of the gene architecture and splice variation of *Lstag-sfc-24*. Transcriptomic contigs similar to Mantle_Edge_v2_idb_51055 were obtained from various *L. stagnalis* transcriptomes and aligned to the corresponding genomic contig gLs.1.0.scaf00135. The genomic contig is indicated by the horizontal black line, the aligned transcripts are indicated by the boxed sections, white boxes represent UTR region, blue boxes represent coding region. Scale bar represents 1000 bp.

**Additional file 26. Spatial expression and molecular features of *Lstag-sfc-*25.** (A) *In situ* hybridisation against *Lstag-sfc-*25 during development and in the fully differentiated adult mantle. The blue staining indicates the expression in the larval mantle edge (iv, iv’) and zone four and five of the adult mantle (v, v’). Each column represents one developmental stage and shows two perspectives. The developmental stage is indicated in the header. The perspective is indicated in the lower right corner. v’ shows a magnification of the boxed section in v. Days post first cleavage (dpfc), lateral (lat.), dorsal (dors.), ventral (ventr.). (B) Protein sequence and schematic representation of the translated Mantle_Edge_v2_idb_44802. The signal sequence (red) and the peptide identified with MS/MS (boxed section) is highlighted.

**Additional file 27. Spatial expression and molecular features of *Lstag-sfc-26*.** (A) *In situ* hybridisation against *Lstag-sfc-26* during development and in the fully differentiated adult mantle. The blue staining indicates the expression across the shell forming tissue in larvae (i-iv, i’-iv’) and zone five of the adult mantle (v, v’). Each column represents one developmental stage and shows two perspectives. The developmental stage is indicated in the header. The perspective is indicated in the lower right corner. v’ shows a magnification of the boxed section in v. Days post first cleavage (dpfc), lateral (lat.), dorsal (dors.), ventral (ventr.). (B) Protein sequence and schematic representation of the translated Mantle_Edge_v2_Tri_114584. The signal sequence (red), the repeated motif (blue) and the peptide identified with MS/MS (boxed section) are indicated.

**Additional file 28. Spatial expression and molecular features of *Lstag-sfc-*27.** (A) *In situ* hybridisation against *Lstag-sfc-*27 during development and in the fully differentiated adult mantle. The blue staining indicates the expression across the shell forming tissue in larvae (i-iv, i’-iv’) and zone five of the adult mantle (v, v’). Each column represents one developmental stage and shows two perspectives. The developmental stage is indicated in the header. The perspective is indicated in the lower right corner. v’ shows a magnification of the boxed section in v. Days post first cleavage (dpfc), lateral (lat.), dorsal (dors.), ventral (ventr.). (B) Protein sequence and schematic representation of the translated Mantle_Edge_v2_idb_25973. The signal sequence (red), the repeated motif (blue) and the peptides identified with MS/MS (boxed sections) are indicated. (C) Protein sequence and schematic representation of the translated Mantle_Edge_v2_idb_25972. The signal sequence (red), the repeated motif (blue) and the peptides identified with MS/MS (boxed sections) are indicated.

**Additional file 29. Spatial expression and molecular features of *Lstag-sfc-28*.** (A) *In situ* hybridisation against *Lstag-sfc-28* during development and in the fully differentiated adult mantle. The blue staining indicates the expression across the shell forming tissue in larvae (ii-iv, ii’-iv’) and zone five of the adult mantle (v, v’). Each column represents one developmental stage and shows two perspectives. The developmental stage is indicated in the header. The perspective is indicated in the lower right corner. v’ shows a magnification of the boxed section in v. Days post first cleavage (dpfc), lateral (lat.), dorsal (dors.), ventral (ventr.). (B) Protein sequence and schematic representation of the translated Mantle_Edge_v2_idb_25543. The signal sequence (red), the repeated motifs (blue) and the peptides identified with MS/MS (boxed sections) are indicated.

**Additional file 30. Spatial expression and molecular features of *Lstag-sfc-29*.** (A) *In situ* hybridisation against *Lstag-sfc-29* during development and in the fully differentiated adult mantle. blue staining indicates the expression in the larval mantle edge (iv, iv’) and zone four and five of the adult mantle (v, v’). Each column represents one developmental stage and shows two perspectives. The developmental stage is indicated in the header. The perspective is indicated in the lower right corner. v’ shows a magnification of the boxed section in v. Days post first cleavage (dpfc), lateral (lat.), dorsal (dors.), ventral (ventr.). (B) Protein sequence and schematic representation of the translated Mantle_Edge_v2_idb_75685. The signal sequence (red), the repeated motif (blue) and the peptide identified with MS/MS (boxed section) are indicated.

**Additional file 31. Spatial expression and molecular features of *Lstag-sfc-30*.** (A) *In situ* hybridisation against *Lstag-sfc-30* during development and in the fully differentiated adult mantle. The blue staining indicates the expression across the shell forming tissue in larvae (ii-iv, ii’-iv’) and zone five of the adult mantle (v, v’). Each column represents one developmental stage and shows two perspectives. The developmental stage is indicated in the header. The perspective is indicated in the lower right corner. v’ shows a magnification of the boxed section in v. Days post first cleavage (dpfc), lateral (lat.), dorsal (dors.), ventral (ventr.). (B) Incomplete protein sequence of the translated Mantle_Proximal_v2_Tri_90027. The peptides identified with MS/MS (boxed sections) are indicated.

**Additional file 32. Spatial expression and molecular features of *Lstag-sfc-31*.** (A) *In situ* hybridisation against *Lstag-sfc-31* during development and in the fully differentiated adult mantle. The blue staining indicates the expression in the larval mantle edge (iv, iv’) and zone one to five of the adult mantle (v, v’). Each column represents one developmental stage and shows two perspectives. The developmental stage is indicated in the header. The perspective is indicated in the lower right corner. v’ shows a magnification of the boxed section in v. Days post first cleavage (dpfc), lateral (lat.), dorsal (dors.), ventral (ventr.). (B) Protein sequence and schematic representation of the translated Mantle_Edge_v2_Tri_9904. The signal sequence (red), the repeated motifs (blue), the conserved sushi domain (underlined black and highlighted grey) and the peptides identified with MS/MS (boxed sections) are indicated.

**Additional file 33. Spatial expression and molecular features of *Lstag-sfc-32*.** (A) *In situ* hybridisation against *Lstag-sfc-32* during development and in the fully differentiated adult mantle. The blue staining indicates the expression in larvae (i-iv, i’-iv’) and along the inner and outer epithelium of the adult mantle (v, v’). Each column represents one developmental stage and shows two perspectives. The developmental stage is indicated in the header. The perspective is indicated in the lower right corner. v’ shows a magnification of the boxed section in v. Days post first cleavage (dpfc), lateral (lat.), dorsal (dors.), ventral (ventr.). (B) Protein sequence and schematic representation of the translated Mantle_Edge_v2_Tri_206671. The conserved intermediate filament protein domain (underlined black and highlighted grey) and the peptides identified with MS/MS (boxed sections) are indicated. (C) Schematic representation of the gene architecture and splice variation of *Lstag-sfc-32*. Transcriptomic contigs similar to Mantle_Edge_v2_Tri_206671 were obtained from various *L. stagnalis* transcriptomes and aligned to the corresponding genomic contig gLs.1.0.scaf00652. The genomic contig is indicated by the horizontal black line, the aligned transcripts are indicated by the boxed sections, white boxes represent UTR region, blue boxes represent coding region. Scale bar represents 1000 bp.

**Additional file 34. Spatial expression and molecular features of *Lstag-sfc-33*.** (A) *In situ* hybridisation against *Lstag-sfc-33* during development and in the fully differentiated adult mantle. The blue staining indicates the expression in larvae (iii, iv, iii’, iv’) and the adult mantle (v, v’). Each column represents one developmental stage and shows two perspectives. The developmental stage is indicated in the header. The perspective is indicated in the lower right corner. v’ shows a magnification of the boxed section in v. Days post first cleavage (dpfc), lateral (lat.), anterior (ant.), ventral (ventr.). (B) Protein sequence and schematic representation of the translated Mantle_Edge_v2_idb_53476. The signal sequence (red), the repeated motif (blue) and the conserved von Willebrand factor type A domain (underlined black and highlighted grey) and the peptides identified with MS/MS (boxed sections) are indicated.

**Additional file 35. Spatial expression and molecular features of *Lstag-sfc-34*.** (A) *In situ* hybridisation against *Lstag-sfc-34* during development and in the fully differentiated adult mantle. The blue staining indicates the expression in larvae (i-iv, i’-iv’). Each column represents one developmental stage and shows two perspectives. The developmental stage is indicated in the header. The perspective is indicated in the lower right corner. v’ shows a magnification of the boxed section in v. Days post first cleavage (dpfc), lateral (lat.), anterior (ant.), ventral (ventr.). (B) Protein sequence and schematic representation of the translated Mantle_Edge_v2_idb_111997. The signal sequence (red) and the peptide identified with MS/MS (boxed section) are highlighted.

**Additional file 36. Extended schematic summary of spatial gene expression profiles and prominent molecular features of 34 *L. stagnalis* shell-forming candidates.** Highlighted are right (dark grey) and left (light grey) asymmetrically expressed shell-forming gene candidates in the trochophore larva, as well as genes expressed broadly across the shell field (light blue). Cells in this region of the trochophore are likely to give rise to cells in zone 5 of the adult mantle, and we have maintained that colour scheme to suggest this. Cells bordering the larval shell gland and shell field (black ring in the trochophore) are likely to give rise to one or more zones 1-4 in the adult mantle. Many genes could also be categorised as either being expressed in the left or right sides, or continuously throughout the free edge of the mantle that produces the outer lip of the shell in juveniles. The number of repetitive low-complexity domains (RLCDs) present in each shell-forming candidate are summarised numerically here. Details of these repeated domains can be seen in Additional file 38. The names of enzymes and other molecular features indicated in zones 1-5 on the schematic of the adult mantle are summarised from [45]. Sequence similarity and conserved domains in the final column of the table are summarised from a number of BLAST searches against SwissProt (SP), the non-redundant (NR) NCBI database and the Conserved Domain (CD) database. See Additional files 40-42 for the results of all BLAST and domain searches.

**References**

1. Weiss IM, Schönitzer V, Eichner N, Sumper M. The chitin synthase involved in marine bivalve mollusk shell formation contains a myosin domain. FEBS Letters. 2006;580:1846-1852.

2. Suzuki M, Sakuda S, Nagasawa H. Identification of chitin in the prismatic layer of the shell and a chitin synthase gene from the Japanese pearl oyster, *Pinctada fucata*. Bioscience, Biotechnology, and Biochemistry. 2007;71:1735-1744.

3. Yonezawa M, Sakuda S, Yoshimura E, Suzuki M. Molecular cloning and functional analysis of chitinases in the fresh water snail, *Lymnaea stagnalis*. Journal of Structural Biology. 2016;196:107-118.

4. Suzuki M, Saruwatari K, Kogure T, Yamamoto Y, Nishimura T, Kato T, Nagasawa H. An acidic matrix protein, Pif, is a key macromolecule for nacre formation. Science. 2009;325:1388-1390.

5. Jackson DJ, McDougall C, Woodcroft B, Moase P, Rose RA, Kube M, Reinhardt R, Rokhsar DS, Montagnani C, Joubert C. Parallel evolution of nacre building gene sets in molluscs. Molecular Biology and Evolution. 2010;27:591-608.

6. Joubert C, Piquemal D, Marie B, Manchon L, Pierrat F, Zanella-Cléon I, Cochennec-Laureau N, Gueguen Y, Montagnani C. Transcriptome and proteome analysis of *Pinctada margaritifera* calcifying mantle and shell: focus on biomineralization. BMC Genomics. 2010;11:613.

7. Montagnani C, Marie B, Marin F, Belliard C, Riquet F, Tayalé A, Zanella-Cléon I, Fleury E, Gueguen Y, Piquemal D. *Pmarg*-Pearlin is a matrix protein involved in nacre framework formation in the pearl oyster *Pinctada margaritifera*. ChemBioChem. 2011;12:2033-2043.

8. Weiner S, Traub W. X-ay diffraction study of the insoluble organic matrix of mollusk shells. FEBS Letters. 1980;111:311-316.

9. Osuna-Mascaró AJ, Cruz-Bustos T, Marin F, Checa AG. Ultrastructure of the Interlamellar Membranes of the Nacre of the Bivalve *Pteria hirundo*, Determined by Immunolabelling. PLOS ONE. 2015;10:e0122934.

10. Nudelman F. Nacre biomineralisation: A review on the mechanisms of crystal nucleation. Seminars in Cell & Developmental Biology. 2015;46:2-10.

11. Machado J, Reis ML, Coimbra J, Sá C. Studies on chitin and calcification in the inner layers of the shell of *Anodonta cygnea*. Journal of Comparative Physiology B. 1991;161:413-418.

12. Zentz F, Bédouet L, Almeida MJ, Milet C, Lopez E, Giraud M. Characterization and quantification of chitosan extracted from nacre of the abalone *Haliotis tuberculata* and the oyster *Pinctada maxima*. Marine Biotechnology. 2001;3:36-44.

13. Furuhashi T, Beran A, Blazso M, Czegeny Z, Schwarzinger C, Steiner G. Pyrolysis GC/MS and IR spectroscopy in chitin analysis of molluscan shells. Bioscience, Biotechnology, and Biochemistry. 2009;73:93-103.

14. Weiss IM, Renner C, Strigl MG, Fritz M. A simple and reliable method for the determination and localization of chitin in abalone nacre. Chemistry of Materials. 2002;14:3252-3259.

15. Caufrier F, Martinou A, Dupont C, Bouriotis V. Carbohydrate esterase family 4 enzymes: substrate specificity. Carbohydrate research. 2003;338:687-692.

16. Arakane Y, Dixit R, Begum K, Park Y, Specht CA, Merzendorfer H, Kramer KJ, Muthukrishnan S, Beeman RW. Analysis of functions of the chitin deacetylase gene family in *Tribolium castaneum*. Insect Biochemistry and Molecular Biology. 2009;39:355-365.

17. Suzuki M, Iwashima A, Kimura M, Kogure T, Nagasawa H. The molecular evolution of the Pif family proteins in various species of mollusks. Marine Biotechnology. 2013;15:145-158.

18. Weiner S. Aspartic acid-rich proteins: major components of the soluble organic matrix of mollusk shells. Calcified Tissue International. 1979;29:163-167.

19. Addadi L, Weiner S. Interactions between acidic proteins and crystals: stereochemical requirements in biomineralization. Proceedings of the National Academy of Sciences. 1985;82:4110-4114.

20. Weiner S, Traub W, Parker SB. Macromolecules in mollusc shells and their functions in biomineralization [and Discussion]. Philosophical Transactions of the Royal Society B: Biological Sciences. 1984;304:425-434.

21. Addadi L, Moradian J, Shay E, Maroudas NG, Weiner S. A chemical model for the cooperation of sulfates and carboxylates in calcite crystal nucleation: relevance to biomineralization. Proceedings of the national academy of sciences. 1987;84:2732-2736.

22. Nudelman F, Gotliv BA, Addadi L, Weiner S. Mollusk shell formation: mapping the distribution of organic matrix components underlying a single aragonitic tablet in nacre. Journal of Structural Biology. 2006;153:176-187.

23. Perl-Treves D, Addadi L. Molecular recognition in pathological crystallizations: Gout. Interactions between Albumin and Sodium Urate Mono-hydrate Crystals. Molecular Crystals and Liquid Crystals. 1990;187:1-16.

24. Addadi L, Weiner S. Control and design principles in biological mineralization. Angewandte Chemie International Edition in English. 1992;31:153-169.

25. Arias JL, Fernández MS. Polysaccharides and proteoglycans in calcium carbonate-based biomineralization. Chemical Reviews. 2008;108:4475-4482.

26. Alberts B, Johnson A, Lewis J, Raff M, Roberts K, Walter P: Molecular Biology of the Cell. 5th revised edition. New York: Garland Science; 2007.

27. Marin F, Bundeleva I, Takeuchi T, Immel F, Medakovic D. Organic matrices in metazoan calcium carbonate skeletons: Composition, functions, evolution. Journal of Structural Biology. 2016;196:98-106.

28. Shen X, Belcher AM, Hansma PK, Stucky GD, Morse DE. Molecular cloning and characterization of lustrin A, a matrix protein from shell and pearl nacre of *Haliotis rufescens*. Journal of Biological Chemistry. 1997;272:32472-32481.

29. Marin F, Corstjens P, Westbroek P, de Gaulejac B, de Vrind-De Jong E. Mucins and molluscan calcification. J Biol Chem. 2000;275:20667-20675.

30. Jackson DJ, Wörheide G, Degnan BM. Dynamic expression of ancient and novel molluscan shell genes during ecological transitions. BMC Evolutionary Biology. 2007;7:160.

31. Evans JS. „Tuning in“ to mollusk shell nacre-and prismatic-associated protein terminal sequences. Implications for biomineralization and the construction of high performance inorganic- organic Composites. Chemical Reviews. 2008;108:4455-4462.

32. Lamport DTA, Kieliszewski MJ, Chen Y, Cannon MC. Role of the extensin superfamily in primary cell wall architecture. Plant physiology. 2011;156:11-19.

33. Mann K, Weiss IM, André S, Gabius H-J, Fritz M. The amino-acid sequence of the abalone (*Haliotis laevigata*) nacre protein perlucin. European Journal of Biochemistry. 2000;267:5257-5264.

34. Weiss IM, Kaufmann S, Mann K, Fritz M. Purification and characterization of perlucin and perlustrin, two new proteins from the shell of the mollusc *Haliotis laevigata*. Biochemical and Biophysical Research Communications. 2000;267:17-21.

35. Wang N, Lee Y-H, Lee J. Recombinant perlucin nucleates the growth of calcium carbonate crystals: molecular cloning and characterization of perlucin from disk abalone, *Haliotis discus discus*. Comparative Biochemistry and Physiology Part B: Biochemistry and Molecular Biology. 2008;149:354-361.

36. Marie B, Joubert C, Tayaléa A, Zanella-Cléon I, Belliard C, Piquemal D, Cochennec-Laureau N, Marin F, Gueguen Y, Montagnani C. Different secretory repertoires control the biomineralization processes of prism and nacre deposition of the pearl oyster shell. Proceedings of the National Academy of Sciences. 2012;109:20986-20991.

37. Mann K, Edsinger E. The *Lottia gigantea* shell matrix proteome: re-analysis including MaxQuant iBAQ quantitation and phosphoproteome analysis. Proteome Science. 2014;12:28.

38. Mann K, Jackson DJ. Characterization of the pigmented shell-forming proteome of the common grove snail *Cepaea nemoralis*. BMC Genomics. 2014;15:249.

39. Liu C, Li S, Kong J, Liu Y, Wang T, Xie L, Zhang R. In-depth proteomic analysis of shell matrix proteins of *Pinctada fucata*. Scientific Reports. 2015;5:17269.

40. O’Keeffe AH, Green JL, Grainger M, Holder AA. A novel Sushi domain-containing protein of *Plasmodium falciparum*. Molecular and Biochemical Parasitology. 2005;140:61-68.

41. Tu Z, Cohen M, Bu H, Lin F. Tissue distribution and functional analysis of Sushi domain-containing protein 4. The American Journal of Pathology. 2010;176:2378-2384.

42. Arivalagan J, Marie B, Sleight VA, Clark MS, Berland S, Marie A. Shell matrix proteins of the clam, *Mya truncata*: Roles beyond shell formation through proteomic study. Marine Genomics. 2016;27:69-74.

43. Pavat C, Zanella-Cléon I, Becchi M, Medakovic D, Luquet G, Guichard N, Alcaraz G, Dommergues J-L, Serpentini A, Lebel J-M, Marin F. The shell matrix of the pulmonate land snail *Helix aspersa maxima*. Comparative Biochemistry and Physiology Part B: Biochemistry and Molecular Biology. 2012;161:303-314.

44. Herrmann H, Bär H, Kreplak L, Strelkov SV, Aebi U. Intermediate filaments: from cell architecture to nanomechanics. Nature Reviews Molecular Cell Biology. 2007;8:562-573.

45. Timmermans LPM. Studies on shell formation in molluscs. Netherlands Journal of Zoology. 1969;19:413-523.
