# Supplementary material for: Molecular modularity and asymmetry of the molluscan mantle revealed by a gene expression atlas
Source: Gigascience. 2018 May 17;7(6):giy056. doi: 10.1093/gigascience/giy056 (PMC6007483; doi:10.1093/gigascience/giy056)
Supplement: GIGA-D-17-00319_Revision-2.pdf [file giy056_giga-d-17-00319_revision-2.pdf]

GigaScience

Molecular modularity and asymmetry of the molluscan mantle revealed by a gene expression atlas

--Manuscript Draft--

**Manuscript Number:**	GIGA-D-17-00319R2
**Full Title:**	Molecular modularity and asymmetry of the molluscan mantle revealed by a gene expression atlas
**Article Type:**	Research
**Funding Information:**	Deutsche Forschungsgemeinschaft (JA 2108/2-1) — Dr. Daniel John Jackson<br>Volkswagen Foundation (DE) (92075) — Dr. Daniel John Jackson
**Abstract:**	Conchiferan molluscs construct a biocalcified shell that likely supported much of their evolutionary success. However beyond broad proteomic and transcriptomic surveys of molluscan shells and the shell-forming mantle tissue, little is known of the spatial and ontogenetic regulation of shell fabrication. In addition, most efforts have been focused on species that deposit nacre, which is at odds with the majority of conchiferan species that fabricate shells using a crossed lamellar microstructure, sensu lato. By combining proteomic and transcriptomic sequencing with in situ hybridisation we have identified a suite of gene products associated with the production of the crossed-lamellar shell in Lymnaea stagnalis. With this spatial expression data we are able to generate novel hypotheses of how the adult mantle tissue coordinates the deposition of the calcified shell. These hypotheses include functional roles for unusual and otherwise difficult to study proteins such as those containing repetitive low complexity domains (RLCDs). The spatial expression readouts of the shell-forming genes we studied also reveal cryptic patterns of asymmetry and modularity in the shell-forming cells of larvae and adult mantle tissue. This molecular modularity of the shell-forming mantle tissue hints at an intimate association between structure, function and evolvability and may provide an elegant explanation for the evolutionary success of the second largest phylum among the Metazoa.
**Corresponding Author:**	Daniel John Jackson, PhD<br>Georg-August-Universitat Gottingen<br>Göttingen, Niedersachsen GERMANY
**Corresponding Author Secondary Information:**	
**Corresponding Author's Institution:**	Georg-August-Universitat Gottingen
**Corresponding Author's Secondary Institution:**	
**First Author:**	Ines Herlitze, PhD
**First Author Secondary Information:**	
**Order of Authors:**	Ines Herlitze, PhD<br>Benjamin Marie, PhD<br>Frédéric Marin, PhD<br>Daniel John Jackson, PhD
**Order of Authors Secondary Information:**	
**Response to Reviewers:**	N/A (proxy upload by HZ)
**Additional Information:**	
**Question**	**Response**
Are you submitting this manuscript to a special series or article collection?	No

Powered by Editorial Manager® and ProduXion Manager® from Aries Systems Corporation

**Experimental design and statistics**<br><br>Full details of the experimental design and statistical methods used should be given in the Methods section, as detailed in our Minimum Standards Reporting Checklist. Information essential to interpreting the data presented should be made available in the figure legends.<br><br>Have you included all the information requested in your manuscript?	Yes
**Resources**<br><br>A description of all resources used, including antibodies, cell lines, animals and software tools, with enough information to allow them to be uniquely identified, should be included in the Methods section. Authors are strongly encouraged to cite Research Resource Identifiers (RRIDs) for antibodies, model organisms and tools, where possible.<br><br>Have you included the information requested as detailed in our Minimum Standards Reporting Checklist?	Yes
**Availability of data and materials**<br><br>All datasets and code on which the conclusions of the paper rely must be either included in your submission or deposited in publicly available repositories (where available and ethically appropriate), referencing such data using a unique identifier in the references and in the “Availability of Data and Materials” section of your manuscript.<br><br>Have you have met the above requirement as detailed in our Minimum Standards Reporting Checklist?	Yes

Powered by Editorial Manager® and ProduXion Manager® from Aries Systems Corporation

Manuscript Click here to download Manuscript Herlitze et al revised3_5_18.docx

Molecular modularity and asymmetry of the molluscan mantle revealed by a gene expression atlas

Ines Herlitze[1], Benjamin Marie[2], Frédéric Marin[3], Daniel J. Jackson[1*]

[1] Department of Geobiology, Georg-August University of Göttingen, Goldschmidtstrasse 3, 37077 Göttingen, Germany

[2] UMR 7245 MNHN/CNRS Molécules de Communication et Adaptation des Micro-organismes, Département Aviv, Sorbonne Universités, Muséum National d'Histoire Naturelle, CP 39, 12 Rue Buffon, 75005 Paris, France.

[3] UMR CNRS 6282 Biogéosciences, Université de Bourgogne - Franche-Comté, 6 Boulevard Gabriel, 21000 Dijon, France

***Author for correspondence:**

Daniel J. Jackson

Department of Geobiology

Georg-August University of Göttingen

Goldschmidtstrasse 3, 37077

Göttingen, Germany

Tel: +49 (0) 55139 14177

email: djackso@uni-goettingen.de

Abstract

Background: Conchiferan molluscs construct a biocalcified shell that likely supported much of their evolutionary success. However beyond broad proteomic and transcriptomic surveys of molluscan shells and the shell-forming mantle tissue, little is known of the spatial and ontogenetic regulation of shell fabrication. In addition, most efforts have been focused on species that deposit nacre, which is at odds with the majority of conchiferan species that fabricate shells using a crossed lamellar microstructure, *sensu lato*.

Results: By combining proteomic and transcriptomic sequencing with *in situ* hybridisation we have identified a suite of gene products associated with the production of the crossed-lamellar shell in *Lymnaea stagnalis*. With this spatial expression data we are able to generate novel hypotheses of how the adult mantle tissue coordinates the deposition of the calcified shell. These hypotheses include functional roles for unusual and otherwise difficult to study proteins such as those containing repetitive low complexity domains (RLCDs). The spatial expression readouts of shell-forming genes also reveal cryptic patterns of asymmetry and modularity in the shell-forming cells of larvae and adult mantle tissue

Conclusions: . This molecular modularity of the shell-forming mantle tissue hints at an intimate association between structure, function and evolvability and may provide an elegant explanation for the evolutionary success of the second largest phylum among the Metazoa.

Introduction

Due to its evolutionary significance, impressive materials properties and aesthetic beauty the molluscan shell has long received attention from a wide variety of scientific disciplines

[1–6]. Although molluscan shells are constructed from a complex mixture of $CaCO_3$, carbohydrates [7] [8] and lipids [9], proteins have received the most attention arguably for two main reasons: they can provide deep insight into the evolutionary history of this composite structure; and the techniques for the high throughput study of these molecules are well established and are technically straight forward. Much progress has been made in identifying the components of the shell forming proteome from a variety of gastropod and (primarily) bivalve species (for example [10–17]) largely due to advances in nucleic acid sequencing technologies which, when coupled with high-throughput proteomic surveys of the biomineralised proteome, allow for the rapid generation of extensive lists of shell-associated proteins. However without further validation, genes identified in this way should only be considered as candidate biomineralising molecules. This problem is often compounded by the fact that these proteins often share little to no sequence similarity with proteins from conventional model organisms, making any inference about their function very difficult. This bottleneck represents one of the current major challenges for scientists interested in understanding the mechanisms and evolution of molluscan biomineral formation. While knock-down of individual shell-forming genes via RNAi has been reported in some species of bivalves [10, 18, 19], these assays are rarely validated by protein immuno-detection, and levels of penetrance or statistical quantitation of knock-down phenotypes are rarely reported.

Another approach to gain insight into the function of shell-forming genes is to characterise their spatial expression patterns *in vivo*. We previously adopted this approach in the tropical abalone *Haliotis asinina* with a Sanger EST dataset and characterised the spatial expression patterns of over 20 putative shell-forming genes in juvenile snails [16]. While a

spatial expression pattern in the mantle is not direct evidence of a functional role in calcification, we were able to assign putative functions to genes involved in shell pigmentation [16] and ecological and mineralogical transitions [20]. Here we have combined an NGS transcriptome analysis of adult mantle tissue with a proteomic survey of the adult shell of the freshwater pulmonate gastropod *Lymnaea stagnalis* in order to both compare the resulting data with other similar datasets, and to generate the first ever *in situ* validated ontogenetic transcriptome-scale dataset for a species that forms the most common molluscan shell microstructure, crossed lamellar [21–23]. The high order structure of crossed-lamella which allows it to efficiently deflect and arrest cracks [24–27], coupled with its extremely low organic content (typically <0.5%), has been suggested to be one reason it has enjoyed so much evolutionary success (reviewed in [28]). Recent proteomic studies have been reported for molluscs that build crossed lamellae shells (*Helix aspersa maxima* [29] and *Cepaea nemoralis* [14]), however those studies did not conduct any spatial expression analyses for the shell-forming proteins they identified. In addition to characterising the spatial expression patterns of more than 30 shell-forming candidates in the adult mantle tissue of *L. stagnalis* we have also investigated their spatial expression patterns during development.

Our analyses hint at the potential pleiotropic nature of some of these shell-forming genes, and highlight the dynamic and asymmetric natures of their spatial regulation. A striking result of our analyses in the adult mantle tissue is the degree of spatial modularity displayed by distinct sets of genes. This general observation may contribute to an explanation of why the molluscan shell is apparently so evolvable. With the availability of a draft *L. stagnalis* genome and transcriptome data from a variety of adult tissues we have

also investigated the genetic architectures of our biomineralisation candidates and explored to what extent alternative splicing plays a role in shell formation in *L. stagnalis*. These genes can also be compared with similar datasets from distantly related molluscs that build shells with alternative polymorphs of calcium carbonate (calcite vs. aragonite) and textures (prismatic vs. nacreous vs. crossed lamellae). Such comparisons can generate testable hypotheses regarding which components of the shell-forming toolkit contribute to these differences and which components are required for more fundamental aspects of shell formation.

Methods

Cultivation of adult *L. stagnalis*

Lymnaea stagnalis (Mollusca; Gastropoda; Heterobranchia; Euthyneura; Panpulmonata; Hygrophila; Lymnaeoidea; Lymnaeidae; Lymnaea) does not fall under the German animal protection act §8 and is listed as 'least concern' under the IUCN's list of threatened species. This work was therefore exempt from the University of Göttingen Ethics Committee. Adult specimens of *L. stagnalis* derived from animals originally collected from the Northeimer Seenplatte, Germany (51° 43' 26.5368', 9° 57' 24.75') and from a pond on the north campus of the University of Göttingen, Germany (51° 33' 23.727', 9° 57' 25.617') were kept in a "Stand-alone V30 unit" (Aqua Schwarz) in demineralized water supplemented with ReMineral+ (Dennerle, #7036) to a conductivity of 200 - 220 µS, 23 °C and a pH of 7.5 to 7.9 and a 16:8 light regimen. Five to ten individuals were kept in three or five litre boxes under a constant and low flow rate. Snails were fed *ad libitum* with lettuce and a variety of other vegetables. Under this regime adult snails lay egg masses year round.

Organic matrix extraction from calcified shells

Twelve shells of adult *L. stagnalis* (larger than 3-4 cm in length) were selected for extraction. Prior to further treatment, the columella was delicately cut and removed from each shell. Superficial organic contaminants were removed by incubating pooled shell fragments in 10%, v/v sodium hypochlorite (NaOCl) for 24 h. Fragments were then thoroughly rinsed with water and subsequently ground into a fine powder that was sieved (> 200 µM). This biomineral powder was incubated in 5% v/v NaOCl for 5 h and rinsed twice with MilliQ water. Powdered samples were decalcified overnight at 4 °C in cold 5%, v/v acetic acid which was slowly added by an automated burette (Titronic Universal, Mainz, Germany) at a flow rate of 100 µL every 5 s. The solution (final pH ~4.2) was centrifuged at 3,900 g for 30 min. The resulting acid insoluble matrix (AIM) pellet was rinsed six times with MilliQ water, freeze-dried and weighed. The supernatant containing acetic acid-soluble matrix (ASM) was filtered (Millipore, 5 µM) and concentrated in an Amicon ultra-filtration stirred cell (model 8400, 400 mL) on a Millipore membrane (10 kDa cut-off). The final solution (> 5 mL) was extensively dialysed against 1 L of MilliQ water (six water changes) before being freeze-dried and weighed.

Sample preparation for proteomic analysis

In-solution digestion of unfractionated ASM (0.1 mg) and AIM (1 mg) material was performed as follows. Samples were reduced with 50 µL of 10 mM dithiothreitol in 50 mM NH_4HCO_3 for 30 min at 50 °C. Alkylation was performed with 50 µL of 100 mM iodoacetamide in 50 mM NH_4HCO_3 for 30 min at room temperature in the dark. The solution was then treated with 1 µg of trypsin (proteomic grade; Promega) in 10 µL of

50 mM NH_4HCO_3 overnight at 37 °C. Samples were then dried in a vacuum concentrator and re-suspended in 30 µL of 0.1% trifluoroacetic acid and 2% CH_3CN.

Peptide fractionation and data acquisition

Mass spectrometry (MS) was performed using a Q-Star XL nanospray quadrupole/time-of-flight tandem mass spectrometer, nanospray- Qq-TOF-MS/MS (Applied Biosystems, Villebon-sur-Yvette, France), coupled to an online nanoLC system (Ultimate Famos Switchos from Dionex, Amsterdam, The Netherlands). One microliter of each sample was loaded onto a trap column (PepMap100 C18; 5 µm; 100 Å; 300 µM x 5 mm; Dionex), washed for 3 min at 25 µL/min with 0.05% trifluoroacetic acid/2% acetonitrile, then eluted onto a C18 reverse phase column (PepMap100 C18; 3 µm; 100 Å; 75 µM x 150 mm; Dionex). Peptides were separated at a flow rate of 0.300 µL/min with a linear gradient of 5– 80% acetonitrile in 0.1% formic acid over 120 min. MS data were acquired automatically using ANALYST QS 1.1 software (Applied Biosystems). Following a MS survey scan over *m/z* 400–1600 range, MS/MS spectra were sequentially and dynamically acquired for the three most intense ions over *m/z* 65–2000 range. The collision energy was set by the software according to the charge and mass of the precursor ion. MS and MS/MS data were recalibrated using internal reference ions from a trypsin autolysis peptide at *m/z* 842.51 $[M + H]^+$ and *m/z* 421.76 $[M + 2H]^{2+}$.

Mass spectrometry data analysis

Protein identification was performed using the MASCOT search engine (version 2.1; Matrix Science, London, UK) against translations in all 6 frames of our mantle transcriptomes which possessed BUSCO completeness scores of >98% (see below). LC-MS/MS data were

searched using carbamido-methylation as a fixed modification, and methionine oxidation as a variable modification. The peptide mass and fragment ion tolerances were set to 0.5 Da. The peptide hits (protein score >50; FDR <0.05; 1 missed cleavage allowed) were manually confirmed by the observation of the raw LC-MS/MS spectra with ANALYST QS software (Version 1.1). Quality criteria were the peptide MS value, the assignment of major peaks to uninterrupted y- and b-ion series of at least three to four consecutive amino acids and the match with the *de novo* interpretations proposed by the software. All mass spectrometry data has been deposited with the ProteomeXchange Consortium via PRIDE [30] with the dataset identifiers PXD008547 and 10.6019/PXD008547. Shell-forming candidates *Lstag-sfc*-7, *Lstag-sfc*-8 and *Lstag-sfc*-9 were bioinformatically selected for analysis based on the presence of a signal peptide and their glycine-rich sequences (i.e. they were not detected using the proteomic methods described above).

Bioinformatic analysis of protein sequences

Using the peptides identified from the proteomic survey described above, partial, or in most cases full length, coding sequences were isolated by standard or RACE PCR as described in [31]. In some cases Illumina transcriptome data (see below) was used to clarify the putative complete mRNA. Open reading frames were translated with the ExPASy translate tool [32]. Protein sequences were searched for signal sequences with SignalP 4.1 [33]. The theoretical pI, amino acid composition and number of amino acids were determined using ExPasy ProtParam tool [34]. Tandem repeats were identified with the T-REKS tool [35]. Sequence similarities searches were performed the BLAST algorithm [36] with tBLASTx against nr and dbEST, and BLASTx against SwissProt. Domain searches were performed with CD search [37]. Molecular function was predicted with InterPro Scan [38]. GalNAc O-

glycosylation sites were predicted using the NetOGlyc 4.0 Server [39]. Scaled schematics of protein sequences were generated using Gene Structure Draw [40]. Intron-exon boundaries were mapped to a draft genome of *L. stagnalis* (Dryad accession http://doi.org/10.5061/dryad.r4342) originally reported in [41] using Splign [42]. Similar transcripts were retrieved from the assembled transcriptomes of mantle zones 1-5 combined, mantle zone 5 alone, cephalic tentacle, cephalic lobe, CNS, foot, buccal mass and larval stages 42 hours post first cleavage (hpfc), 52 hpfc and 67 hpfc using BLASTn searches (see below for NGS details). All transcripts with complete open reading frames (ORF) were considered. Only candidates yielding an mRNA coverage of >98% and an overall identity of >98% are documented. Scaled schematics of the gene architecture were generated using Gene Structure Draw [40]. Protein patterns were searched for using a modified local installation of PatMatch [43].

NGS sequencing

Total RNA was extracted from the mantle edge and the proximal mantle tissue of a single adult *L. stagnalis* using TriReagent following the manufacturers instructions. The resulting RNA was processed by the sequencing center at the IKMB at the University of Kiel (Germany). Paired end, stranded TrueSeq RNA libraries were constructed and sequenced for 101 bases from both ends using the Illumina HiSeq2000 platform. More than 99 million and 100 million reads were generated from each of these libraries respectively. These Illumina reads were processed using our pipeline as previously described [44]. Briefly, raw reads were adapter trimmed and quality filtered using Trimmomatic V0.32. Filtered reads were then assembled with Trinity V2.0.3, CLC Genomics Workbench *de novo* assembler (V8.5), and IDBA-tran. The resulting assemblies were then merged and filtered for

redundancy using our pipeline [44]. Mantle transcriptome assemblies and cDNA and protein translations of the 34 shell-forming genes are available in the GigaScience Database, GigaDB [45]. In addition, transcriptomes from 5 adult tissues (cephalic tentacle, cephalic lobe, CNS, foot and buccal mass) and 3 larval stages (42 hours post first cleavage (hpfc), 52 hpfc and 67 hpfc) were sequenced and assembled as described above. These transcriptomes were used to assess the tissue specific alternative splicing characteristics of all shell-forming genes. All raw NGS data has been deposited in the SRA with BioSample accession numbers SAMN08117214, SAMN08117215, SAMN08709370, SAMN08709371, SAMN08709372, SAMN08709373, SAMN08709374, SAMN08709375, SAMN08709376 and SAMN08709377.

In situ hybridisation on whole mounts and sections

Larvae were prepared for whole mount *in situ* hybridisation as described in [46]. Sections (10 μM) were taken from *L. stagnalis* (shell length 10-50 mm), which had been fixed in formaldehyde for 1 hour and embedded in paraffin. Riboprobes were prepared as described in [16] and were used at concentrations of 100 ng – 500 ng/mL. Whole mounts and tissue sections were processed for hybridisation, the colour reaction developed and photo-documented as described in [46].

Comparisons of molluscan shell forming proteomes

BLASTp based comparisons of the *L. stagnalis* shell proteome were performed against a variety of calcifying proteomes reported in a wide phylogenetic range of metazoans as described in [14]. These included: 42 proteins from the oyster *Pinctada maxima* reported in [47]; 78 proteins from the oyster *Pinctada margaritifera* reported in [47]; 94 proteins from

the abalone *Haliotis asinina* reported in [17] and [16]; 80 proteins from the abalone *H. laevigata* reported in [48]. 63 protein from the limpet *Lottia gigantea* reported in [49]; 53 proteins from the oyster *Crassostrea gigas* reported in [50]; 71 proteins from the mussel *Mya truncata* reported in [51]; 59 proteins from the grove snail *Cepaea nemoralis* reported in [14]; 44 proteins from the oyster *Pinctada fucata* reported in [52]; 53 proteins from the mussel *Mytilus coruscus* reported in [53]; 66 proteins from the brachiopod *Magellania venosa* reported in [54]; 139 proteins from the sea urchin *Strongylocentrotus purpuratus* reported in [55]; 37 proteins from the coral *Acropora millepora* reported in [56].

Analysis of the saccharide moieties of the shell matrix

The monosaccharide content of AIM and ASM was obtained by suspension and homogenization (vortex and ultrasound) of lyophilates in 2 M trifluoroacetic acid (TFA) and subsequent hydrolysis at 105 °C for 4 h under a nitrogen atmosphere. This treatment allows for the release of most monosaccharides from complex mixtures, except sialic acids which are destroyed, and the acetylated forms of glucosamine and galactosamine, which are converted to their respective non-acetylated forms. Samples were then centrifuged for 5 min, at 15,000g and evaporated to dryness (using a SpeedVac) before being dissolved in 100 µL of 20 mM NaOH and homogenized. After a short centrifugation (2 min), 80 µL of supernatant was injected into the chromatograph system: the neutral, amino and acidic sugar contents of hydrolysates were determined by HPAE-PAD (High Pressure Anion Exchange - Pulsed Amperometric Detection) on a CarboPac PA 100 column (Dionex Corp., Sunnyvale, CA, USA). As blank controls, non-hydrolysed AIMs were analysed, in order to detect potential free monosaccharides that may lead to an over-representation of some

sugar residues.

Results

Proteomic analysis of the biomineralised matrix of *L. stagnalis* shells

More than 1,230 peptides were analyzed by HPLC-mass spectrometry and subsequently used for protein identification using Mascot against our translated mantle transcriptomes. Of these 1,230 peptides 329 of them returned significant matches. From these 329 matches a total of 40 shell-forming candidate transcripts were identified (see Additional file 1). Of these 40 gene products, 31 (78%) could be cloned and exhibit *in situ* hybridisation signals compatible with a role in shell formation (either in larval stages and/or in the adult mantle tissue). Seven of these 40 candidates (18%) could be cloned from *L. stagnalis* cDNA, but did not produce a positive or consistent *in situ* signal in any tissue. Three of the 40 candidate genes (8%) could not be amplified by gene specific PCR or RACE PCR. In addition to the 31 proteomically identified candidates that generated positive *in situ* signals, three candidates that were identified via *in silico* methods (based purely on the presence of a signal sequence and their glycine-rich protein sequences) also generated *in situ* signals compatible with a role in shell formation and are reported here.

A brief morphological description of *L. stagnalis* shell ontogeny and the adult mantle

We previously described the ontogeny of the shell gland and shell field in *L. stagnalis* [57]. In order to aid the interpretation of our *in situ* patterns the following is a summary of the main developmental stages that we focused on. The first visible sign of differentiation of the shell-forming tissue in *L. stagnalis* is a thickening of the dorsal ectoderm that begins at

approximately 29 hours post first cleavage (hpfc) [57, 58]. These cells subsequently invaginate and by 2 days post first cleavage (dpfc) a clearly visible 'shell gland' is present [57, 58]. By 3 dpfc, the shell gland has formed a sealed lumen, and displays the first signs of outward signs of asymmetry [57]. The marginal cells that border the shell gland remain uninvaginated and form a ring-like structure, the rosette [2]. During this time the first extracellular organic material is secreted and is clearly visible by SEM (Fig. 1; [57]). By 3 dpfc, the shell gland has evaginated to form the shell field. The former rosette cells remain highly elongated while the central cells take on a low columnar appearance. Over the next several days the shell field continues to expand until it has overgrown the visceral mass and will eventually become the adult mantle tissue [2, 57, 58].

The adult mantle covers the inner surface of the shell and is responsible for shell growth and repair. The free edge of the mantle is responsible for the growth of the outer lip of the shell. Timmermans conducted an extensive histochemical characterisation of the mantle tissue of *L. stagnalis* and she was able to categorise the free edge of the adult mantle into six distinct zones based on their morphology, enzymatic activities and biochemical signatures [59]. We largely follow this categorisation of the adult mantle tissue. Parallel to the mantle edge runs the mantle groove (also known as the pallial groove) defined as zone 1 (Fig. 2). Several high-resolution microscopy and histological studies on a variety of molluscs have demonstrated that it is from within the pallial groove that the periostracum is formed and secreted [59–64]. We detected a sub-regionalisation of the pallial groove (zone 1) into proximal and distal zones. Immediately adjacent to the pallial groove is a broad region of high columnar cells referred to by Timmermans [59] as the 'belt' that can be subdivided into three distinct zones (zones 2-4). Zone 2 is immediately adjacent to the posterior wall of

the pallial groove and comprises the anterior (or distal) portion of the belt (Fig. 2). Zone 3 consists of the posterior portion of the belt, while zone 4 represents the transitional zone between the high columnar cells of the belt proper and the more posterior low columnar cells of the outer epithelium which comprise zone 5 (Fig. 2) [59].

Spatial expression patterns and molecular features of shell forming candidate genes

We performed *in situ* hybridisation for 34 distinct shell-forming genes on 4 distinct developmental stages and on adult mantle tissue. The detailed results of these analyses are presented in Additional files 2 – 35 with an extensive summary presented in Additional file 36 (the raw image files that constitute these figures are available in the associated GigaDB repository [45]. In Figure 1 (for larvae) and Figure 2 (for adult mantle tissue) we present a selection of these results that highlight some prominent features of these expression patterns. In trochophore and veliger larval stages (2-6 dpfc) all genes could be categorised as either being expressed in: cells that symmetrically or asymmetrically border the shell gland or shell field (15/34); cells that lay within the shell gland or shell field (9/34); a pattern that did not fit into our classification scheme (1/34); or were not expressed in any detectable way (9/34). In later stages (~7 dpfc) all genes were either expressed: uniformly along the outer edge of the mantle (10/34); asymmetrically in the outer edge of the mantle (18/34); throughout the entire mantle tissue (2/34); a pattern that did not fit into our classification scheme (1/34); or were not expressed in any detectable way (3/34). Finally in adult mantle tissue genes were either expressed: in one or more of the 5 zones described by Timmermans [59] (32/34); a pattern that did not fit into our classification scheme (1/34); or were not expressed in any detectable way (1/34). We have schematically summarised all of these results in Fig. 3.

Alternative splicing increases the diversity of shell-forming proteins

Via alternative splicing of mRNAs, transcripts with a variety of functions can be generated from a single genomic locus [65]. With a draft genome for *L. stagnalis* available we were able to perform some preliminary investigations into alternative splicing of our shell-forming candidates. While some candidate genes displayed the same or very similar exon-splicing patterns in all surveyed tissues (for example Additional files 5, 9, 14, 15 and 16), most candidates are apparently alternatively spliced depending on the tissue they are expressed in (Additional files 4, 11, 12, 18, 19, 22, 23, 25 and 33). Striking examples include *Lstag-sfc-21* and *Lstag-sfc-24* which are expressed in many tissues, but display significant alternative splicing in the adult mantle (Additional files 22 and 25). All splice variants of candidate *Lstag-sfc-24* encode proteins with the same aspartic acid-rich motif (Additional files 25, 37 and 38). Aspartic acid-rich proteins have been suggested to act as an organic template for epitaxial crystal growth [66, 67]. It is tempting to speculate that the three additional domains only present in adult mantle *Lstag-sfc-24* contigs confer a specific shell-forming function to this protein. The putative chitin-interacting candidate *Lstag-sfc-21* presented in Additional file 22 carries a signal sequence and is predicted to possess a catalytic activity. Intriguingly a number of splice variants of this gene within the adult mantle are predicted to lack a signal sequence, the chitin-binding or catalytic ability (Additional file 37).

A number of shell-forming gene candidates produce alternatively spliced transcripts that encode proteins with differences regarding the presence/absence of a signal sequence (Additional files 11, 12, 22, 25 and 37). Some shell-forming genes also produce alternatively

spliced transcripts that encode proteins with similar coding features, but radically different 5' or 3' UTRs (Additional files 12, 19, 33 and 37). While UTRs do not contain protein-coding information, they can be critical for localisation of the mRNA [68, 69] and post-transcriptional gene regulation by molecules such as miRNAs [70]. Indeed several miRNAs have now been associated with the targeting and regulation of biomineralising proteins [71, 72].

Comparisons of molluscan shell forming proteomes

We conducted a broad comparison of our *L. stagnalis* shell forming genes against a wide phylogenetic range of twelve other biomineralising proteomes comprising in total 879 proteins (sequences used in this analysis are provided in Additional file 39). Of all *L. stagnalis* shell proteins, 27 shared significant sequence similarity with one or more proteins derived from one of these twelve proteomes (Fig. 4). The highest degree of overall similarity was found with the shell forming proteome of the common groove snail *C. nemoralis* (Fig. 4), the closest phylogenetic relative to *L. stagnalis* of all species in this comparison. The next highest level of similarity shared with the *L. stagnalis* shell forming proteome (15.9% of the *L. gigantea* shell forming proteome) was markedly lower. *L. stagnalis* shell-forming proteins that shared significant similarity with biomineralizing proteins from other species and that also returned a significant match against a SwissProt entry included *Lstag*-sfc-32 (with similarity to *C. nemoralis* contig 572), which appears to be an intermediate filament protein. *Lstag*-sfc-22 (a gene expressed exclusively in zone 5, Additional file 23) shared relatively weak similarity with *C. nemoralis* contig 821, and shares significant sequence similarity with PIF, an aragonite binding protein reported to be involved in nacre formation in the oyster *P. fucata* [19]. Interestingly, of the 12 candidates

expressed in the matrix-secreting zone 5, nine showed similarity with other shell proteins (Figs. 4, and Additional files 23-33). Eight of them were shared with *C. nemoralis* (Fig. 4). In contrast, none of the asymmetrically expressed or glycine-rich candidates were found in any of the other biomineralizing proteomes (low complexity filtering was inactivated in these comparison; Fig. 4).

Glycosylation patterns of the shell matrix

The monosaccharides profiles of ASMs and AIMs extracted from adult *L. stagnalis* shells were peculiar, with less than half of the dozen standard monosaccharides represented. For ASM, these include galactosamine, glucosamine, galactose, glucuronic acid and glucose, while AIM lacked glucuronic acid (Table 1). We also found marked differences in the glycosylation rates of acid-soluble and acid-insoluble matrices. In general the absolute degree of glycosylation of the ASM was 1-3 orders of magnitude greater that of the AIM. The largest difference was in the amount of galactosamine (10.6 ng/µL vs. 0.03 ng/µL). While glucosamine was more abundant absolutely in the ASM (9.41 ng/µL vs. 0.13 ng/µL), the proportional difference was not so extreme (34.8% vs. 54.2%).

Discussion

Molecular modularity of the adult molluscan mantle

Two of the most striking features of the phylum Mollusca are its size (in terms of number of species) and its diversity. Widely accepted to be second only to the Arthropoda in terms of number of living species [73, 74], molluscs arguably display the greatest diversity of body forms of all metazoan phyla, and have successfully colonized all kinds of environments.

While there currently exists no consensus as to why molluscs have enjoyed such deep evolutionary success (one interesting suggestion includes a plastic nervous system, [75]) we believe the mantle tissue (an apomorphy of the phylum) and its ability to prolifically evolve new shell phenotypes must contribute to an explanation of this success. A logical extension of this question would therefore be, "what is it about the molluscan mantle tissue that makes it so evolutionarily plastic?" For arthropods, segmentation and body plan modularisation (and the underlying gene regulatory networks that control appendage identity within each segment) are widely thought to have played leading roles in supporting the diversification of insects, spiders and crustaceans [76]. The importance of establishing segmentation at a very early developmental age in prominent phyla such as annelids, chordates and arthropods has caused much effort to be spent on identifying the causal molecular mechanisms that may have common evolutionary histories [77–79]. As recently reviewed by Esteve-Altava [80], the presence of morphological modules can help us to understand the evolvability of body form, but the identification of such modules has so far been biased towards mammals, arthropods and plants. Following Esteve-Altava's and Eble's [81] definition of a morphological module (a group of body parts that are more integrated among themselves than they are to other parts outside the group) we propose that the molluscan mantle is a prime example of such a morphological module. This modular nature of the molluscan mantle is not unique to *Lymnaea* [82–84]. Although the precise functions of these zones (and of the individual gene products that define them) await the development of targeted genome editing methods, it is clear that they must act in a coordinated way to deposit the shell. We predict that there are related modules of gene regulatory networks (GRNs) that act to specify each zone of the molluscan mantle, and that it is the modular nature of these GRNs and the resulting morphological modularity of the

mantle tissue that supported the diversification of the phylum Mollusca. Characterisation of the spatial expression patterns of shell-forming proteomes from a selection of other molluscan lineages will contribute to a more refined understanding of molluscan shell evolution.

Ontogenetic expression of shell-forming candidates

A prominent outcome of our survey of the adult shell proteome is that many of the genes that encode these proteins are not only regulated spatially, but also temporally. Many shell-forming candidates are expressed in the invaginated larval shell gland of the trochophore (Additional files 14, 15, 21, 24, 25, 27 and 28) or in cells that border it (Additional files 2, 3, 6, 7, 8, 10, 11 and 18). Only two candidates were solely expressed in the adult mantle tissue and not in any larval stage (Additional files 17 and 23). Timmermans [59] concluded that the spatial patterning of larval shell-forming cells persists throughout development and foreshadows the zonation observable in the adult mantle. We also observed this phenomenon at the molecular level. All candidate genes that were expressed in the margin of the shell gland or the shell field were expressed in the belt (zones 2, 3 and 4) of the adult mantle (Summarised in Fig. 3, Additional files 2-8, 10-12 and 18). Most candidates expressed in the invaginated cells of the shell gland or throughout the developing shell field were subsequently expressed in the low columnar outer epithelium of zone 5 in adult mantle tissue (Summarised in Fig. 3, Additional files 24, 25, 27, 28, 29, 31). However three genes conspicuously deviate from this pattern. *Lstag-sfc-13, -14* and *-20* display a broad expression pattern in the invaginated cells of the shell gland and throughout the entire shell field in larvae, but were not detected in the low columnar outer epithelium (zone 5) of the adult mantle tissue (Additional files 14, 15 and 21). However we should point out that for

all candidate shell-forming genes we did not consider the potential effect of a diurnal rhythm on gene expression. All samples for *in situ* hybridisation were taken during daylight hours, and so genes with activity during the night would be missed.

Asymmetric expression of shell-forming genes

The expression of *Lstag-sfc-1, Lstag-sfc-2* and *Lstag-sfc-3* in zones 1 and 2 of the adult mantle suggests they may be involved in the formation of the periostracum (Additional files 2, 3 and 4), however it is their larval expression patterns that are more striking. *Lstag-sfc-1, -2* and *-3* display a right-sided asymmetric expression pattern in cells bordering the shell gland and shell field. In contrast *Lstag-sfc-17* is expressed on the left side (Additional file 18). Following the expression of these genes ontogenetically into older larvae that begin to display the coiled phenotype of the adult, it is apparent that right-sided cells in the trochophore are likely to be those that give rise to the right + anterior region of the adult mantle that will produce the outer lip of the shell, while left-sided cells will give rise to posterior mantle tissue responsible for forming the left + parietal region of the shell (Fig. 1). We therefore suggest that *Lstag-sfc-1, -2* and *-3* are in some way associated with producing thinner, more rapidly produced shell at the outer shell lip than in the thicker parietal region while *Lstag-sfc-17* may inhibit the rapid deposition of shell. Exactly how this is achieved awaits more specific gene function assays.

In addition to the trochophore left/right asymmetry corresponding to the left + parietal/right + outer lip regions of the shell, there is a second axis of symmetry that becomes apparent in 7-day old juvenile snails. Many shell-forming candidates are initially symmetrically expressed in or surrounding the shell gland of 2-3 dpfc trochophores, but

then become asymmetrically expressed in the mantle of older animals. For example *Lstag-sfc-6, -7, -8, -12, -14, -15, -17, -18, -20, -23, -24, -26, -27, -29 and -31* are expressed in the left side of the free mantle edge in 7 dpfc juveniles (summarised in Fig. 3). In contrast, relatively few shell-forming gene candidates (*Lstag-sfc*-5, *-9*, *-10*, *-21* and *-25)* are expressed evenly along the free edge of the mantle in 7 dpfc juveniles (summarised in Fig. 3).

The spatial expression of a peroxidase in the adult mantle allows a model of shell formation to be developed

In agreement with Timmermans histochemical study of peroxidase activity [59], the expression of *Lstag-sfc-5*, a shell-forming candidate with an "Animal heme-dependent peroxidase" domain (Pfam PF03098**;** Figs. 6B) is localised to zones 1 and 2. Peroxidases may be involved in periostracum formation by cross-linking fibrous proteins rich in reactive quinones to form water insoluble, protease-resistant polymers [85–87]. This process, also referred to as tanning or sclerotisation, can also be catalysed by tyrosinase (also known as catechol oxidase, catecholase, polyphenoloxidase, phenoloxidase and phenolase), and within the molluscan biomineralisation literature sclerotisation by tyrosinase appears to be the more commonly assumed mechanism, rather than by peroxidase. Nonetheless, Timmermans demonstrated that heat inactivation clears the periostracal groove and belt of both peroxidase activity and the ability to form melanin (a typical assay used to test for tyrosinase activity), while specific tyrosinase inhibitors ($NaHSO_3$ and KCN) did not affect its ability to produce melanin [59]. The spatial expression pattern of *Lstag-sfc-5*, coupled with the observations that newly secreted periostracum itself also displays peroxidase activity [57], and Timmermans experiments [59], strongly suggests that the peroxidase we have identified here plays a key role in cross-linking the

periostracum in *L. stagnalis* rather than a tyrosinase, as also supposed for other gastropods such as *Lottia* [15].

Glycine-rich shell-forming candidates are likely to be substrates for the peroxidase

An important aspect of scleroprotein formation is its spatial coordination. The cross-linking reaction often generates cytotoxic intermediates, and the end products cannot be easily degraded or resorbed [88]. Furthermore the uncontrolled formation of extensive scleroprotein polymers prior to secretion would clearly be detrimental to the cell. One common strategy to avoid these events is to compartmentalize the scleroprotein precursor (that is unable to spontaneously polymerize) away from the cross-linking enzyme. Following secretion, the precursors are activated and enzymatically cross-linked [88]. Such a scenario would suggest that the substrate upon which the peroxidase acts are not located within the same cells.

Three candidates expressed in zone three (*Lstag-sfc-6, -7* and *-8*) encode secreted, basic proteins that are dominated by repetitive low complexity domains (RLCDs) and anomalous amino acid contents (high glycine, tyrosine, asparagine and leucine contents; Additional files, 37 and 38). All of these glycine-rich proteins carry tyrosine residues flanked by glycine. This arrangement has been shown to be favourable for the formation of cross-links between tyrosine residues by peroxidase [89]. Waite, in his review of natural quinone-tanned glues, highlighted the typical DOPA-containing consensus precursor peptide sequences from a number of marine invertebrates. Allowing for a single mismatch, these substrate peptides (VGG**Y**G**Y**GK, GGGFGG**Y**GK and GGG**Y**GG**Y**GK, crosslinking tyrosine residues in bold) can be found within *Lstag*-sfc-6*, Lstag*-sfc-7 and *Lstag*-sfc-8. Interestingly,

these glycine-rich candidates are expressed exclusively in zone three (Additional files 7-9) immediately adjacent to zone 2, the region in which the peroxidase *Lstag*-sfc-5 is expressed (Additional file 6). Theoretically, once these proteins are secreted, the secreted peroxidase would be in very close proximity to the glycine-rich proteins and could act on the favoured tyrosine residues to form di-tyrosine cross-links extracellularly.

A role for immunity and signalling in shell formation

Lstag-sfc-18 contains two Ig superfamily domains (Additional file 19; [90]) and displays sequence similarity with the IMP-L2 like proteins (Additional files 40 and 41), an insulin-like growth factor binding protein (IGF-BP) that carries two immunoglobulin-like domains and is able to bind insulin-like growth factors (IGF) [91]. Several studies by Dogterom and colleagues demonstrated the influence of a growth hormone secreted by the cerebral ganglia specifically on shell formation in *L. stagnalis* [92–95]. The authors conclude that this growth hormone acts on cells in the belt region to control shell extension and periostracum formation, but not on shell thickening. Interestingly Perlustrin, a protein associated with nacre in abalone shells, contains an IGF-BP domain and was also shown to bind IGFs and insulin [96]. An intriguing idea for the presence of IGF-BP in the abalone shell is that it would allow the shell to signal to the underlying mantle epithelium. According to this hypothesis, IGFs present in the extrapallial fluid are bound by IGF-BP during calcification and incorporated into the shell. Should the shell dissolve or be locally damaged, these IGFs would be released and subsequently stimulate the underlying mantle epithelium to re-calcify. One line of evidence that strongly supports this hypothesis is provided by the osteogenic activity of mollusc shells [97]. This hypothesis implies that although the shell is acellular, it is able to actively communicate and provide real-time feedback to the mantle

epithelium [98]. This interesting hypothesis awaits the development of gene specific knock-down or knock-out assays.

RLCDs are an abundant feature of *L. stagnalis* shell proteins

Proteins containing repetitive low-complexity domains (RLCDs) are a prominent feature of molluscan shell-forming proteomes [15, 99, 100], and *L. stagnalis* is no exception. More than half of the *L. stagnalis* shell-forming candidates we identified possess RLCDs. Proteins containing these domains were present in the belt and the low columnar outer epithelium of the adult mantle and in a wide variety of patterns of the larval stages we investigated. The motif complexity, motif length and number of motif repeats can vary greatly, from stretches consisting of a single amino acid (for example Additional file 24), to motifs that exceed ten amino acids (for example Additional files 3, 4, 29 and 34). In some cases, almost the whole protein is composed of RLCDs (Additional files 7-10). Repeated motifs are a common feature of structural proteins such as collagens, keratins, silk and cell wall proteins, as well as structural modules in functional proteins such as receptors, histones, ion channels and transcription factors [101, 102]. RLCDs are often part of intrinsically unstructured regions that lack a fixed or ordered three-dimensional structure [101]. In some cases, these regions define the functionality of the protein. As a general rule, unstructured proteins interact readily with other proteins [103], and the highly repetitive, modular and biased amino acid compositions can confer strength and elasticity [104]. It will be extremely informative to selectively remove RLCDs from shell-forming proteins and to study the resulting shell phenotypes once genome modification tools become broadly available to molluscs.

Broad sequence similarity comparisons of metazoan biomineralizing proteomes

The crossed-lamellar microstructure is fabricated by phylogenetically diverse molluscan taxa and is by far the most commonly employed shell design of the Conchifera [21, 22, 28]. While much attention has been dedicated to the characterisation of nacre-forming bivalve shell proteomes, technical advances in nucleic acid sequencing and proteome-scale surveys has seen a rapid growth in the number and diversity of molluscan shell-forming proteomes, and allows broad comparisons of these datasets to be performed. These comparisons can provide insight into the degree of evolutionary conservation that exists across shell-forming proteomes [50]. In general, molluscan shell-forming proteomes are markedly different, with some deeply conserved elements such as alkaline phosphatases, peroxidases and carbonic anhydrases [28, 57, 105, 106]. The significant diversity of molluscan shell ultrastructures, crystal textures, colours, and materials properties therefore cannot be explained by the use of the same genes in different ways. Rather, each lineage has uniquely evolved a large fraction of its shell-forming proteome [14–16, 100, 107]. To expand on this comparative theme we collected a total of 879 biomineralising proteins validated by proteomics from a total of ten molluscs, one brachiopod, one sea urchin and one coral, and performed sequence similarity comparisons against our *L. stagnalis* dataset. Two of the ten molluscs, *Cepaea nemoralis* and *Mya truncata* [51, 108], construct shells that contain crossed lamellar texture. Interestingly our comparative analyses show that *L. stagnalis* and *M. truncata* have only three proteins that share relatively low degrees of sequence similarity, while *L. stagnalis* and *C. nemoralis* share seventeen proteins (some of these with very high degrees of sequence similarity), the highest extent of similarity between all species surveyed (Fig. 4). Both *L. stagnalis* and *C. nemoralis* inhabit non-marine environments, and the similarities in their shell proteomes may either be a manifestation of this, and/or a reflection of their

crossed lamellar shells. The similarity of their shell protein content may also reflect the relatively recent divergence time (Meso-Cenozoic) of these two clades (Stylommatophoran, *i.e. C. nemoralis* vs. hygrophilid, *i.e., L. stagnalis*) within the monophyletic order of pulmonate gastropods, in comparison to the other species. One of the most striking observations we made in these comparisons was that almost all *L. stagnalis* shell-forming candidates expressed in zone 5 share sequence similarity with *C. nemoralis*. Conversely, *L. stagnalis* shell-forming candidates expressed asymmetrically on the right side in larvae were not present in any other biomineralising proteomes.

Some *L. stagnalis* shell-forming proteins contain domains found in a number of the biomineralizing proteins present in the dataset we assembled, or are known to play a role in processes other than biomineralization such as the Sushi domain, the von Willebrand factor A domain, the immunoglobulin domain and the filament protein domain [14, 109]. The Pif-like protein is prevalent in both bivalve and gastropod nacreous shell proteomes, and is known to bind aragonite crystals and to regulate nacre formation [110]. However limpets, which construct crossed lamellar structures, also contain Pif in their shells [15, 110, 111]. Our results further demonstrate that Pif is not limited to nacreous matrices, and that it is likely to be a deeply conserved element of the molluscan biomineralizing proteome.

Glycosylation patterns

Our preliminary analysis of the sugar moieties associated with shell-forming proteins revealed an interesting dichotomy between the ASM and AIM; the population of ASM proteins appear to be far more glycosylated than AIM proteins (Table 1). Whether this

difference is generated by a heavily glycosylated subset of the ASM, or if it reflects a general trend of most ASM proteins being glycosylated remains unknown. We also cannot determine whether there are any spatial biases within the adult mantle tissue with regards to the location of glycosylated proteins. The high percentage of glucosamine identified in AIM and ASM suggests that chitin, or its deacetylated derivative chitosan, is present in both extracts but this hypothesis requires further testing. Despite their likely importance to the functional mechanisms of shell formation, post-translational modifications of molluscan shell-forming proteins remain relatively understudied, and we predict that research efforts in these directions would yield interesting functional insights into the mechanisms of shell fabrication.

Conclusion

By characterising the spatial expression patterns of 34 genes associated with shell-formation we have revealed patterns of asymmetry that presumably contribute to the coiled phenotype of *Lymnaea's* shell. Our broad survey of these genes in the adult mantle tissue also highlight the morphological modularity of this phylum specific organ, and allude to an explanation as to why the Mollusca have evolved so many successful shell morphologies. While gene co-option, domain shuffling and gene family expansion are mechanisms that have clearly contributed to the great diversity of molluscan shell-forming proteins, our analyses also suggest that alternative splicing acts to significantly expand the shell-forming molecular repertoire. Comparing the results of spatial gene expression surveys focused on shell-formation from a broad range of molluscan taxa will shed further light on the evolutionary story of this fascinating structure.

Acknowledgements

We are grateful to Wolfgang Dröse for assistance and advice with *in situ* sectioning, Isabelle Zanella-Cleon from IBCP (Lyon) for mass spectrometry analysis and Jennifer Hohagen and Dorothea Hause-Reitner who generated SEM images. Illumina sequencing was performed by Markus B. Schilhabel and his team at the Institute of Clinical Molecular Biology, Christian-Albrechts-University Kiel. This work was funded by DFG (JA 2108/2-1 and JA 2108/6-1) and VolkswagenStiftung (92075) grants to DJJ.

Author Contributions

IH carried out the molecular work, bioinformatic analyses, co-wrote and drafted the manuscript. FM and BM performed the proteomic analyses and drafted the manuscript. DJJ conceived and supervised the study, contributed to the molecular work, contributed to the bioinformatic analyses, performed the histological sections, co-wrote and drafted the manuscript. All authors read and approved the final manuscript.

Competing financial interests

The authors declare no competing financial interests.

Availability of data and materials

All raw NGS data has been deposited with the SRA with BioSample accession numbers SAMN08117214, SAMN08117215, SAMN08709370, SAMN08709371, SAMN08709372, SAMN08709373, SAMN08709374, SAMN08709375, SAMN08709376 and SAMN08709377. Individual image files for the *in situ* hybridisation gene expression patterns and the sense strand cDNA sequences used to generate the *in situ* hybridisation riboprobes can be

accessed from the associated GigaDB repository [45]. The mantle transcriptome assemblies are also available via GigaDB [45] (file names: C2844_CLC_idba_Trinity_for_annotation.fasta and C2845_CLC_idba_Trinity_for_annotation.fasta). All mass spectrometry data has been deposited with the ProteomeXchange Consortium with the dataset identifiers PXD008547 and 10.6019/PXD008547. Other supporting data is available from additional files, also including an extended description of the *in situ* hybridization results (see additional file 47).

Table 1: Glycosylation analysis of acid-soluble and acid-insoluble matrices extracted from adult *L. stagnalis* shells.

	ASM		AIM	
**Monosaccharide**	ng/µg	%	ng/µg	%
Fucose	ND	-	TR	-
Rhamnose	ND	-	TR	-
Galactosamine	10.60	39.2	0.03	12.5
Arabinose	ND	-	ND	-
Glucosamine	9.41	34.8	0.13	54.2
Galactose	4.34	16.0	0.04	16.7
Glucose	0.32	1.2	0.04	16.7
Mannose	ND	-	TR	-
Xylose	ND	-	ND	-
Galacturonic acid	ND	-	ND	-
Glucuronic acid	2.39	8.8	ND	-
**Total**	**27.06**	**100.0**	**0.24**	**100.0**

ND = not detected, TR = trace.

Figure legends

Fig. 1. Overview of four developmental stages and representative shell-forming gene expression patterns in *L. stagnalis.* The first two rows provide a set of reference SEM images and adult shells (top-right-most panel) against which the images of the *in situ* results can be oriented. All *in situ* panels are from a dorsal view except the right-most column which is from a ventral view. Here we present the expression patterns of a selection of 5 different shell-forming genes. These include genes with expression patterns in shell-forming cells that display evidence of symmetry (*sfc-5*), right asymmetry (*sfc-1*), left-asymmetry (*sfc-17*), expression entirely throughout the shell field and dorsal mantle epithelium (*sfc-20*) and expression in additional non-shell-forming cells. This last expression pattern provides evidence of genes involved in shell-formation that have pleiotropic functions. The scale bars in the first row are 100 μm. Indicated in the SEM images are the positions of the foot lobe (fl), foot (f), mantle margin (mm), calcified shell (s), stomodeum (st) and insoluble organic material (iom) of the shell.

Fig. 2. Overview of the adult *L. stagnalis* shell-forming mantle tissue and representative shell-forming gene expression patterns that reveal its molecular modularity. A. A semi-thin sagittal section of an adult *L. stagnalis* stained with Giemsa. The foot (f), mantle (m), digestive gland (dg) and radula (r) are indicated. The mantle tissue is a thin sheet of epithelium that covers the dorsal surface of the adult animal and is responsible for fabricating the shell. **B.** A magnified view of the red-boxed region in A reveals some of the cellular morphology of the adult mantle tissue. **C.** A schematic representation of the

mantle tissue divided into 6 zones as described by Timmermans [59]. The spatial distribution of enzymatic activities and biochemicals indicated in this schematic are adapted from [59]. We detect a sub-regionalisation of the pallial groove (zone 1) into proximal (light green) and distal (dark green) zones. **D.** The spatial expression patterns of 8 representative shell-forming genes in the adult mantle tissue. The asterisk indicates that *sfc*-6 was identified using *in silico* methods rather than proteomic methods.

Figure 3. Summary of the spatial gene expression profiles and conserved features of 34 *L. stagnalis* shell-forming candidates. Schematically represented in a trochophore larva are genes with an asymmetric expression profile (dark grey), as well as genes expressed broadly across the shell field (light blue). Cells in this region of the trochophore are likely to give rise to cells in zone 5 of the adult mantle, and we have maintained that colour scheme to suggest this. Although we schematically present a trochophore larva here (2-3 dpfc) the summarised expression patterns also include veliger stages (3-6 dpfc). Cells bordering the larval shell gland and shell field (black ring in the trochophore) are likely to give rise to one or more zones 1-4 in the adult mantle. In juveniles (~7 dpfc) many genes were either expressed in the left, right, or continuously throughout the free edge of the mantle that produces the outer lip of the shell. Question marks indicate expression patterns that could not be categorised according to our scheme. An "x" indicates no expression was detected. A "?" indicates that the expression pattern could not be categorised according to our scheme. The names of enzymes and other molecular features indicated in zones 1-5 on the schematic of the adult mantle are summarised from [59] and [3]. Sequence similarity and conserved domains in the final column of the table are summarised from a number of BLAST searches against SwissProt (SP), the non-redundant (NR) NCBI database and the

Conserved Domain (CD) database. See Additional files 40-42 for the results of all BLAST and domain searches. The linneages of the top BLAST hits are listed in Additional file 43. A version of this figure that includes a more complete summary of the molecular features of each gene is provided in Additional file 36. The asterisks indicate that *sfc-6, -7* and *-8* were identified using *in silico* methods rather than proteomic methods (as was the case for all other gene products presented here).

Figure. 4. BLASTp comparisons of the *L. stagnalis* shell proteome against 879 biocalcifying proteins derived from 6 bivalves, 4 gastropods, 1 brachiopod, 1 sea urchin and 1 coral. Individual lines spanning the ideogram connect proteins that share significant similarity (e values < $10e^{-6}$). Transparent red lines connect proteins with the lowest quartile of similarity (with a threshold of $10e^{-6}$) and green lines with the highest quartile of similarity. The percentage of each shell proteome that shared similarity with the *L. stagnalis* proteome is indicated. The table provides further information for those candidates that share sequence similarity with another species. Abbreviations: conserved domain database (CD); Genbank non-redundant protein database (NR); SwissProt database (SP).

Summary of supplementary material.

Additional file 1: Summarised results of MASCOT searches.
Additional files 2-35: Whole mount *in situ* hybridisation results and molecular features of 34 shell-forming gene candidates.
Additional file 36: A more comprehensive summary of the results presented in Figure 3.
Additional file 37: Detailed table of the molecular features of all shell-forming protein candidates.
Additional file 38: Results of repetitive motif searches using Xstream.
Additional file 39: A FASTA formatted file of the 879 protein sequences used to construct Figure 4.
Additional file 40: Detailed results of tBLASTx similarity searches for all shell-forming candidate genes against nr database.
Additional file 41: Detailed results of protein family and protein domain similarity searches for all shell-forming candidate genes against CDD database.
Additional file 42: Detailed results of BLAST similarity searches for all shell-forming candidate genes against SwissProt database.
Additional file 43: Lineages for all shell-forming candidates that returned positive BLAST results.
Additional file 44: Nucleotide sequences of 34 families of shell forming candidate genes.
Additional file 45: Translated sequences of 34 families of shell forming candidate genes.
Additional file 46: mRNA regions targeted by riboprobes.
Additional file 47: Extended Results and Discussion.

References

1. Mao L-B, Gao H-L, Yao H-B, Liu L, Cölfen H, Liu G, Chen S-M, Li S-K, Yan Y-X, Liu Y-Y. Synthetic nacre by predesigned matrix-directed mineralization. Science. 2016;354:107-110.
2. Kniprath E. Ontogeny of the molluscan shell field: a review. Zool Script. 1981;10:61-79.
3. Jackson DJ, Degnan BM. The importance of Evo-Devo to an integrated understanding of molluscan biomineralisation. J Struct Biol. 2016;196:67-74.
4. Okabe T, Yoshimura J. Optimal designs of mollusk shells from bivalves to snails. Sci Rep. 2017;7:42445.
5. Aguilera F, McDougall C, Degnan BM. Co-option and de novo gene evolution underlie molluscan shell diversity. Mol Biol Evol. 2017;34:779-792.
6. Liang J, Xie J, Gao J, Xu C-Q, Yan Y, Jia G-C, Xiang L, Xie L-P, Zhang R-Q. Identification and Characterization of the Lysine-Rich Matrix Protein Family in *Pinctada fucata*: Indicative of Roles in Shell Formation. Mar Biotech. 2016;18:645-658.
7. Marxen JC, Hammer M, Gehrke T, Becker W. Carbohydrates of the organic shell matrix and the shell-forming tissue of the snail *Biomphalaria glabrata* (Say). Biol Bull. 1998;194:231-240.
8. Arias JL, Fernández MS. Polysaccharides and proteoglycans in calcium carbonate-based biomineralization. Chem Rev. 2008;108:4475-4482.
9. Farre B, Dauphin Y. Lipids from the nacreous and prismatic layers of two Pteriomorpha Mollusc shells. Comp Biochem Physiol B. 2009;152:103-109.
10. Zhao M, He M, Huang X, Wang Q, Shi Y. Functional characterization and molecular mechanism exploration of three granulin epithelin precursor splice variants in biomineralization of the pearl oyster *Pinctada fucata*. Mol Genet Genom. 2016;291:399-409.
11. Wang J, Gao J, Xie J, Zheng X, Yan Y, Li S, Xie L, Zhang R. Cloning and mineralization-related functions of the calponin gene in *Chlamys farreri*. Comp Biochem Physiol B. 2016;201:53-58.
12. Yarra T, Gharbi K, Blaxter M, Peck LS, Clark MS. Characterization of the mantle transcriptome in bivalves: *Pecten maximus*, *Mytilus edulis* and *Crassostrea gigas*. Marine Genomics. 2016;27:9-15.
13. Gao P, Liao Z, Wang X-x, Bao L-f, Fan M-h, Li X-m, Wu C-w, Xia S-w. Layer-by-layer proteomic analysis of *Mytilus galloprovincialis* shell. PloS ONE. 2015;10:e0133913.
14. Mann K, Jackson DJ. Characterization of the pigmented shell-forming proteome of the common grove snail *Cepaea nemoralis*. BMC Genom. 2014;15:249.
15. Marie B, Jackson DJ, Ramos-Silva P, Zanella-Cleon I, Guichard N, Marin F. The shell-forming proteome of *Lottia gigantea* reveals both deep conservations and lineage-specific novelties. FEBS J. 2013;280:214-232.
16. Jackson DJ, McDougall C, Green K, Simpson F, Wörheide G, Degnan BM. A rapidly evolving secretome builds and patterns a sea shell. BMC Biol. 2006;4:40.
17. Marie B, Marie A, Jackson DJ, Dubost L, Degnan BM, Milet C, Marin F. Proteomic analysis of the organic matrix of the abalone *Haliotis asinina* calcified shell. Prot Sci. 2010;8:54.
18. Zhao M, He M, Huang X, Wang Q. A homeodomain transcription factor gene, *PfMSX*,

activates expression of *Pif* gene in the pearl oyster *Pinctada fucata*. PloS ONE. 2014;9:e103830.
19. Suzuki M, Saruwatari K, Kogure T, Yamamoto Y, Nishimura T, Kato T, Nagasawa H. An acidic matrix protein, Pif, is a key macromolecule for nacre formation. Science. 2009;325:1388-1390.
20. Jackson DJ, Wörheide G, Degnan BM. Dynamic expression of ancient and novel molluscan shell genes during ecological transitions. BMC Evol Biol. 2007;7:160.
21. Dauphin Y, Denis A. Structure and composition of the aragonitic crossed lamellar layers in six species of Bivalvia and Gastropoda. Comparative Biochemistry and Physiology Part A: Molecular & Integrative Physiology. 2000;126:367-377.
22. de Paula SM, Silveira M. Studies on molluscan shells: contributions from microscopic and analytical methods. Micron. 2009;40:669-690.
23. Almagro I, Drzymała P, Berent K, Sainz-Díaz CI, Willinger M-G, Bonarski J, Checa AG. New crystallographic relationships in biogenic aragonite: the crossed-lamellar microstructures of mollusks. Crystal Growth & Design. 2016
24. Kuhn-Spearing LT, Kessler H, Chateau E, Ballarini R, Heuer AH, Spearing SM. Fracture mechanisms of the *Strombus gigas* conch shell: implications for the design of brittle laminates. Journal of Materials Science. 1996;31:6583-6594.
25. Kamat S, Su X, Ballarini R, Heuer AH. Structural basis for the fracture toughness of the shell of the conch *Strombus gigas*. Nature. 2000;405:1036-1040.
26. Pokroy B, Zolotoyabko E. Microstructure of natural plywood-like ceramics: a study by high-resolution electron microscopy and energy-variable X-ray diffraction. Journal of Materials Chemistry. 2003;13:682-688.
27. Rodriguez-Navarro AB, Checa A, Willinger M-G, Bolmaro R, Bonarski J. Crystallographic relationships in the crossed lamellar microstructure of the shell of the gastropod *Conus marmoreus*. Acta Biomaterialia. 2012;8:830-835.
28. Marin F, Luquet G, Marie B, Medakovic D. Molluscan shell proteins: primary structure, origin, and evolution. Curr Top Dev Biol. 2008;80:209-276.
29. Pavat C, Zanella-Cléon I, Becchi M, Medakovic D, Luquet G, Guichard N, Alcaraz G, Dommergues J-L, Serpentini A, Lebel J-M, Marin F. The shell matrix of the pulmonate land snail *Helix aspersa maxima*. Comp Biochem Physiol B. 2012;161:303-314.
30. Vizcaíno JA, Csordas A, Del-Toro N, Dianes JA, Griss J, Lavidas I, Mayer G, Perez-Riverol Y, Reisinger F, Ternent T. 2016 update of the PRIDE database and its related tools. Nucleic Acids REesearch. 2016;44:D447-D456.
31. Jackson DJ, Ellemor N, Degnan BM. Correlating gene expression with larval competence, and the effect of age and parentage on metamorphosis in the tropical abalone *Haliotis asinina*. Mar Biol. 2005;147:681-697.
32. Gasteiger E, Gattiker A, Hoogland C, Ivanyi I, Appel RD, Bairoch A. ExPASy: the proteomics server for in-depth protein knowledge and analysis. Nuc Acids Res. 2003;31:3784-3788.
33. Petersen TN, Brunak S, von Heijne G, Nielsen H. SignalP 4.0: discriminating signal peptides from transmembrane regions. Nat Meth. 2011;8:785-786.
34. Gasteiger E, Hoogland C, Gattiker A, Wilkins MR, Appel RD, Bairoch A: Protein identification and analysis tools on the ExPASy server. Springer; 2005.
35. Jorda J, Kajava AV. T-REKS: identification of Tandem REpeats in sequences with a K-meanS based algorithm. Bioinformatics. 2009;25:2632-2638.
36. Altschul SF, Gish W, Miller W, Myers EW, Lipman DJ. Basic local alignment search tool.

J Mol Biol. 1990;215:403-410.
37. Marchler-Bauer A, Derbyshire MK, Gonzales NR, Lu S, Chitsaz F, Geer LY, Geer RC, He J, Gwadz M, Hurwitz DI. CDD: NCBI's conserved domain database. Nuc Acids Res. 2014gku1221.
38. Mitchell A, Chang H-Y, Daugherty L, Fraser M, Hunter S, Lopez R, McAnulla C, McMenamin C, Nuka G, Pesseat S. The InterPro protein families database: the classification resource after 15 years. Nuc Acids Res. 2014gku1243.
39. Steentoft C, Vakhrushev SY, Joshi HJ, Kong Y, Vester-Christensen MB, Katrine T, Schjoldager BG, Lavrsen K, Dabelsteen S, Pedersen NB. Precision mapping of the human *O*-GalNAc glycoproteome through SimpleCell technology. The EMBO journal. 2013;32:1478-1488.
40. Gene Structure Draw. [http://www.compgen.uni-muenster.de/tools/strdraw/index.hbi?] [http://www.compgen.uni-muenster.de/tools/strdraw/index.hbi?]
41. Davison A, McDowell GS, Holden JM, Johnson HF, Koutsovoulos GD, Liu MM, Hulpiau P, Van Roy F, Wade CM, Banerjee R. Formin is associated with left-right asymmetry in the pond snail and the frog. Curr Biol. 2016;26:654-660.
42. Kapustin Y, Tőzsér J, Souvorov A, Tatusova T, Lipman D. Splign: algorithms for computing spliced alignments with identification of paralogs. Biology Direct. 2008;3:20.
43. Yan T, Yoo D, Berardini TZ, Mueller LA, Weems DC, Weng S, Cherry JM, Rhee SY. PatMatch: a program for finding patterns in peptide and nucleotide sequences. Nuc Acids Res. 2005;33:W262-W266.
44. Cerveau N, Jackson DJ. Combining independent de novo assemblies optimizes the coding transcriptome for nonconventional model eukaryotic organisms. BMC Bioinformatics. 2016;17:525.
45. Herlitze I, Marie B, Marin F, Jackson DJ. Supporting data for "Molecular modularity and asymmetry of the molluscan mantle revealed by a gene expression atlas". GigaScience Database 2018. http://dx.doi.org/10.5524/100436.
46. Jackson DJ, Herlitze I, Hohagen J. A whole mount *in situ* hybridization method for the gastropod mollusc *Lymnaea stagnalis*. JoVE. 2016e53968.
47. Marie B, Joubert C, Tayaléa A, Zanella-Cléon I, Belliard C, Piquemal D, Cochennec-Laureau N, Marin F, Gueguen Y, Montagnani C. Different secretory repertoires control the biomineralization processes of prism and nacre deposition of the pearl oyster shell. Proceedings of the National Academy of Sciences. 2012;109:20986-20991.
48. Karlheinz Mann, Nicolas Cerveau, Meike Gummich, Fritz M, Jackson DJ. In-depth proteomic analyses of *Haliotis laevigata* (greenlip abalone) nacre and prismatic organic shell matrix. Submitted.
49. Mann K, Edsinger E. The *Lottia gigantea* shell matrix proteome: re-analysis including MaxQuant iBAQ quantitation and phosphoproteome analysis. Prot Sci. 2014;12:28.
50. Feng D, Li Q, Yu H, Kong L, Du S. Identification of conserved proteins from diverse shell matrix proteome in *Crassostrea gigas*: characterization of genetic bases regulating shell formation. Sci Rep. 2017;7:45754.
51. Arivalagan J, Marie B, Sleight VA, Clark MS, Berland S, Marie A. Shell matrix proteins of the clam, *Mya truncata*: Roles beyond shell formation through proteomic study. Marine Genomics. 2016;27:69-74.
52. Liu C, Li S, Kong J, Liu Y, Wang T, Xie L, Zhang R. In-depth proteomic analysis of shell

matrix proteins of *Pinctada fucata*. Sci Rep. 2015;5:17269.
53. Liao Z, Bao L-f, Fan M-h, Gao P, Wang X-x, Qin C-l, Li X-m. In-depth proteomic analysis of nacre, prism, and myostracum of Mytilus shell. Journal of Proteomics. 2015;122:26-40.
54. Jackson DJ, Mann K, Häussermann V, Schilhabel MB, Lüter C, Griesshaber E, Schmahl W, Wörheide G. The *Magellania venosa* biomineralizing proteome: a window into brachiopod shell evolution. Gen Biol Evol. 2015;7:1349-1362.
55. Mann K, Poustka AJ, Mann M. In-depth, high-accuracy proteomics of sea urchin tooth organic matrix. Prot Sci. 2008;6:33.
56. Ramos-Silva P, Kaandorp J, Herbst F, Plasseraud L, Alcaraz G, Stern C, Corneillat M, Guichard N, Durlet C, Luquet G, Marin F. The Skeleton of the Staghorn Coral *Acropora millepora*: Molecular and Structural Characterization. PLoS ONE. 2014;9:e97454.
57. Hohagen J, Jackson DJ. An ancient process in a modern mollusc: early development of the shell in *Lymnaea stagnalis*. BMC Dev Biol. 2013;13:27.
58. Kniprath E. Zur Ontogenese des Schalenfeldes von *Lymnaea stagnalis*. Wilhelm Roux's Archives of Developmental Biology. 1977;181:11-30.
59. Timmermans LPM. Studies on shell formation in molluscs. Neth J Zool. 1969;19:413-523.
60. Saleuddin ASM. An electron microscopic study on the formation of the periostracum in *Helisoma* (Mollusca). Calcified Tissue International. 1975;18:297-310.
61. Kniprath E. Formation and structure of the periostracum in *Lymnaea stagnalis*. Cal Tiss Res. 1972;9:260-271.
62. Bubel A. An electron-microscope study of periostracum formation in some marine bivalves. I. The origin of the periostracum. Mar Biol. 1973;20:213-221.
63. Bevelander G, Nakahara H. An electron microscope study of the formation of the periostracum of *Macrocallista maculata*. Cal Tiss Res. 1967;1:55-67.
64. Bubel A. An electron-microscope study of periostracum formation in some marine bivalves. II. The cells lining the periostracal groove. Mar Biol. 1973;20:222-234.
65. Ast G. How did alternative splicing evolve. Nat Rev Gen. 2004;5:773-782.
66. Weiner S, Traub W, Parker SB. Macromolecules in mollusc shells and their functions in biomineralization [and Discussion]. Philosophical Transactions of the Royal Society B: Biological Sciences. 1984;304:425-434.
67. Addadi L, Weiner S. Interactions between acidic proteins and crystals: stereochemical requirements in biomineralization. Proceedings of the National Academy of Sciences. 1985;82:4110-4114.
68. Kingsley EP, Chan XY, Duan Y, Lambert JD. Widespread RNA segregation in a spiralian embryo. Evol Dev. 2007;9:527-539.
69. Lambert JD, Nagy LM. Asymmetric inheritance of centrosomally localized mRNAs during embryonic cleavages. Nature. 2002;420:682-686.
70. Bartel DP, Chen C-Z. Micromanagers of gene expression: the potentially widespread influence of metazoan microRNAs. Nat Rev Gen. 2004;5:396-400.
71. Zheng Z, Jiao Y, Du X, Tian Q, Wang Q, Huang R, Deng Y. Computational prediction of candidate miRNAs and their potential functions in biomineralization in pearl oyster *Pinctada martensii*. Saudi Journal of Biological Sciences. 2016;23:372-378.
72. Jiao Y, Zheng Z, Tian R, Du X, Wang Q, Huang R. MicroRNA, pm-miR-2305, participates in nacre formation by targeting pearlin in pearl oyster *Pinctada martensii*. Int J Molec Sci. 2015;16:21442-21453.

73. Brusca RC, Brusca GJ: Invertebrates. 2nd edition. Sinauer Associates Sunderland, Massachusetts; 2002.
74. Rosenberg G. A new critical estimate of named species-level diversity of the recent Mollusca. Am Malacol Bull. 2014;32:308-322.
75. Hochner B, Glanzman DL. Evolution of highly diverse forms of behavior in molluscs. Curr Biol. 2016;26:R965-R971.
76. Williams TA, Nagy LM. Developmental modularity and the evolutionary diversification of arthropod limbs. Journal of Experimental Zoology Part A: Ecological Genetics and Physiology. 2001;291:241-257.
77. Peel AD, Chipman AD, Akam M. Arthropod segmentation: beyond the *Drosophila* paradigm. Nat Rev Gen. 2005;6:905-916.
78. Tautz D. Segmentation. Developmental Cell. 2004;7:301-312.
79. Raff RA: The shape of life: genes, development, and the evolution of animal form. University of Chicago Press; 1996.
80. Esteve-Altava B. In search of morphological modules: a systematic review. Biol Rev. 2017;92:1332-1347.
81. Eble GJ. Morphological modularity and macroevolution. In: Callebaut W, Rasskin-Gutman D, editors. Modularity: understanding the development and evolution of natural complex systems. MIT Press, Cambridge; 2005. p. 221-238.
82. McDougall C, Green K, Jackson DJ, Degnan BM. Ultrastructure of the mantle of the gastropod *Haliotis asinina* and mechanisms of shell regionalization. Cells Tissues Organs. 2011;194:103-107.
83. Jolly C, Berland S, Milet C, Borzeix S, Lopez E, Doumenc D. Zonal localization of shell matrix proteins in mantle of *Haliotis tuberculata* (Mollusca, Gastropoda). Mar Biotech. 2004;6:541-551.
84. Sleight VA, Marie B, Jackson DJ, Dyrynda EA, Marie A, Clark MS. An Antarctic molluscan biomineralisation tool-kit. Sci Rep. 2016;6:36978.
85. Waite JH. The phylogeny and chemical diversity of quinone-tanned glues and varnishes. Comp Biochem Physiol B. 1990;97:19-29.
86. Waite JH. Evidence for the mode of sclerotization in a molluscan periostracum. Comp Biochem Physiol B. 1977;58:157-162.
87. Waite JH, Wilbur KM. Phenoloxidase in the periostracum of the marine bivalve *Modiolus demissus* Dillwyn. J Exp Zool. 1976;195:359-367.
88. Waite JH. Quinone-tanned scleroproteins. In: Saleuddin ASM, Wilbur KM, editors. Physiology. New York: Academic Press; 1983. p. 467-504.
89. Michon T, Chenu M, Kellershon N, Desmadril M, Guéguen J. Horseradish peroxidase oxidation of tyrosine-containing peptides and their subsequent polymerization: a kinetic study. Biochem. 1997;36:8504-8513.
90. Halaby DM, Mornon JPE. The immunoglobulin superfamily: an insight on its tissular, species, and functional diversity. Journal of Molecular Evolution. 1998;46:389-400.
91. Honegger B, Galic M, Köhler K, Wittwer F, Brogiolo W, Hafen E, Stocker H. Imp-L2, a putative homolog of vertebrate IGF-binding protein 7, counteracts insulin signaling in *Drosophila* and is essential for starvation resistance. Journal of biology. 2008;7:1.
92. Dogterom AA, Doderer A. A hormone dependent calcium-binding protein in the mantle edge of the freshwater snail *Lymnaea stagnalis*. Calcified Tissue International. 1981;33:505-508.
93. Dogterom AA, Jentjens T. The effect of the growth hormone of the pond snail *Lymnaea*

stagnalis on periostracum formation. Comparative Biochemistry and Physiology Part A: Physiology. 1980;66:687-690.
94. Dogterom AA, van Loenhout H, van der Schors RC. The effect of the growth hormone of *Lymnaea stagnalis* on shell calcification. Gen Comp Endocrinol. 1979;39:63-68.
95. Dogterom AA, van der Schors RC. The effect of the growth hormone of *Lymnaea stagnalis* on (bi) carbonate movements, especially with regard to shell formation. Gen Comp Endocrinol. 1980;41:334-339.
96. Weiss IM, Göhring W, Fritz M, Mann K. Perlustrin, a *Haliotis laevigata (*abalone) nacre protein, is homologous to the insulin-like growth factor binding protein N-terminal module of vertebrates. Biochem Biophys Res Commun. 2001;285:244-249.
97. Zhang G, Willemin AS, Brion A, Piet M-H, Moby V, Bianchi A, Mainard D, Galois L, Gillet P, Rousseau M. A new method for the separation and purification of the osteogenic compounds of nacre Ethanol Soluble Matrix. J Struct Biol. 2016;196:127-137.
98. Marin F, Luquet G. Molluscan shell proteins. Comptes Rendus Palevol. 2004;3:469-492.
99. Shen X, Belcher AM, Hansma PK, Stucky GD, Morse DE. Molecular cloning and characterization of lustrin A, a matrix protein from shell and pearl nacre of *Haliotis rufescens*. J Biol Chem. 1997;272:32472-32481.
100. Kocot KM, Aguilera F, McDougall C, Jackson DJ, Degnan BM. Sea shell diversity and rapidly evolving secretomes: insights into the evolution of biomineralization. Front Zool. 2016;13:23.
101. Luo H, Nijveen H. Understanding and identifying amino acid repeats. Briefings in Bioinformatics. 2014;15:582-591.
102. Albà M, Tompa P, Veitia R. Amino acid repeats and the structure and evolution of proteins. In: editors. Gene and Protein Evolution. Karger Publishers; 2007. p. 119-130.
103. Alberts B, Johnson A, Lewis J, Raff M, Roberts K, Walter P: Molecular Biology of the Cell. 5th revised edition. New York: Garland Science; 2007.
104. McDougall C, Woodcroft BJ, Degnan BM. The widespread prevalence and functional significance of silk-like structural proteins in metazoan biological materials. PLoS ONE. 2016;11:e0159128.
105. Le Roy N, Jackson D, Marie B, Ramos-Silva P, Marin F. Carbonic anhydrase and metazoan biocalcification: a focus on molluscs. In: Marin F, Brümmer F, Checa A, Furtos G, Lesci IG, Šiller L, editors. Biomineralization: from fundamentals to biomaterials & environmental issues. Trans Tech Publications Ltd; 2015. p. 151-157.
106. Sun X, Yang A, Wu B, Zhou L, Liu Z. Characterization of the mantle transcriptome of Yesso scallop (*Patinopecten yessoensis*): identification of genes potentially involved in biomineralization and pigmentation. PloS one. 2015;10:e0122967.
107. Jackson DJ, McDougall C, Woodcroft B, Moase P, Rose RA, Kube M, Reinhardt R, Rokhsar DS, Montagnani C, Joubert C. Parallel evolution of nacre building gene sets in molluscs. Mol Biol Evol. 2010;27:591-608.
108. Sleight VA, Thorne MAS, Peck LS, Arivalagan J, Berland S, Marie A, Clark MS. Characterisation of the mantle transcriptome and biomineralisation genes in the blunt-gaper clam, *Mya truncata*. Marine Genomics. 2016;27:47-55.
109. Arivalagan J, Yarra T, Marie B, Sleight VA, Duvernois-Berthet E, Clark MS, Marie A, Berland S. Insights from the shell proteome: biomineralization to adaptation. Mol Biol Evol. 2016msw219.
110. Suzuki M, Kogure T, Weiner S, Addadi L. Formation of aragonite crystals in the crossed lamellar microstructure of limpet shells. Crystal Growth & Design. 2011;11:4850-

4859.
111. Mann K, Edsinger-Gonzales E, Mann M. In-depth proteomic analysis of a mollusc shell: acid-soluble and acid-insoluble matrix of the limpet *Lottia gigantea*. Prot Sci. 2012;10:28.

Table Click here to download Table Table 1.docx

Table 1: Glycosylation analysis of acid-soluble and acid-insoluble matrices extracted from adult *L. stagnalis* shells.

	ASM		AIM	
**Monosaccharide**	ng/µg	%	ng/µg	%
Fucose	ND	-	TR	-
Rhamnose	ND	-	TR	-
Galactosamine	10.60	39.2	0.03	12.5
Arabinose	ND	-	ND	-
Glucosamine	9.41	34.8	0.13	54.2
Galactose	4.34	16.0	0.04	16.7
Glucose	0.32	1.2	0.04	16.7
Mannose	ND	-	TR	-
Xylose	ND	-	ND	-
Galacturonic acid	ND	-	ND	-
Glucuronic acid	2.39	8.8	ND	-
**Total**	**27.06**	**100.0**	**0.24**	**100.0**

ND = not detected, TR = trace.
