# Supplementary material for: Molecular modularity and asymmetry of the molluscan mantle revealed by a gene expression atlas
Source: Gigascience. 2018 May 17;7(6):giy056. doi: 10.1093/gigascience/giy056 (PMC6007483; doi:10.1093/gigascience/giy056)
Supplement: Response-to-Reviewer-Comments_Original-Submission.pdf [file giy056_response-to-reviewer-comments_original-submission.pdf]

Author's Response To Reviewer Comments

We are very grateful for the time and effort that all four reviewers have invested in our manuscript and we are certain that their insightful comments and proof reading have raised the standard of our submission. Please find below our responses to all issues raised. We hope the editor and reviewers find them satisfactory.

Reviewer Deborah Power:

Comment 1:
The MS by Herlitz et al., is exciting and timely as it addresses a fundamental question linked to the role of the mantle in shell plasticity and the evolutionary success of the molluscs. In this comprehensive study the authors use transcriptomics, proteomics and in situ hybridisation to map transcripts and gain insight into the shell secreting tissue and how it links to shell asymmetry. As the authors point out there have now been a number of transcriptomic and in situ studies aimed at identifying the molecules involved in shell construction (frequently in gastropods and also in with some bivalves) but far fewer studies exist of the shell proteome and no studies exist as far as I am aware linking the proteome with the transcriptome. The authors propose that the proteome gives far more compelling evidence of proteins involved in shell formation than transcriptomic data. I agree to some extent with this statement in so far that the proteins are functional. However, I do not think that positional mapping to the mantle is necessarily direct evidence of a function in calcification and so the authors should be a bit more circumspect and avoid categorical statements. The authors clearly and convincingly demonstrate how their study significantly advances the state of the art and the in situ part of the study is of excellent quality. Having said all the above, I think that the MS could be improved. I expected the various subsections in the methods and results to be in the same order but this does not seem to occur; some of the methods are insufficiently described and results pop-up, which are difficult to link with the methods (genomic scaffolds). The results are over descriptive and should be more incisive by going straight to the point. The methods are over brief and cite other articles and so miss out important details needed for quality assessment. In contrast the results go over data previously reported (e.g. section 1), while this is useful, I think it could be shortened. In summary, I appreciated this study but the MS should be better written and organised to do full justice to the data and to make the MS easier to read. I outline below some aspects of the study that could be improved.

Response 1:
Several of the above valuable comments are also raised by Reviewer 2 and we have addressed those below (for example rearranging parts of the results and more information regarding the mass spec work). We have also tempered our discussion regarding the assumption that a distinct spatial expression pattern of a secreted protein in the mantle is not necessarily evidence of that protein playing a direct role in shell-formation. For example:
"While a spatial expression pattern in the mantle is not direct evidence of a functional role in calcification, we were able to assign putative functions to genes involved in shell pigmentation [16] and ecological and mineralogical transitions [20]." (page 3, lines 62-65).

Information regarding the source of the draft genome (and the genomic scaffolds) and the

methods used to map our shell-forming genes to those scaffolds is available on Page 8, line 185-187 as follows:

“Intron-exon boundaries were mapped to a draft genome of L. stagnalis (Dryad accession http://doi.org/10.5061/dryad.r4342) originally reported in [41] using Splign [42].”

Regarding the “overly descriptive” results section we have maintained the discussion as a separate section from the results as requested by the journal. We have also already moved more detailed descriptions of the in situ patterns into the supplementary material. Finally, the results section is currently 3.5 pages (out of 30 pages not including references) and 1,156 words (out of 8,059 words not including references). We would also point out that in reviewer 3’s opinion “It is succinctly written for such an extensive dataset.”

Regarding the comment “…the MS should be better written and organised to do full justice to the data and to make the MS easier to read", we have attempted to make some modifications to improve things including re-ordering the results section to reflect the order of sections in the methods section. We would also point out that in reviewer 3’s opinion “This is a really well written paper describing a comprehensive investigation into biomineralization genes in L. stagnalis.”

Comment 2:
1. Methods
a) It would be helpful to have a better phylogenetic designation of the species in the methods to allow a better appreciation of the significance of the observations across the molluscs.

Response 2:
We have now included the lineage for Lymnaea stagnalis at the beginning of the Methods section (see page 5, lines 104-105).

Comment 3:
b) The proteomics is generally well performed. However, I suggest that the authors update their Mascot searches since this work was conducted in 2012 (31st August, 2012) and since then a lot more data from molluscs has been deposited in NCBI. Furthermore, it is unclear if a species filter was used to search the EST databases used for the proteomics analysis or if all ESTs were used. Taking into consideration the emphasis given in the introduction to the difficulty of making interference about mollusc gene function due to limited sequence conservation with more conventional models I was expecting to see some selection of the EST dataset used for analysis. While I can understand that EST databases may generate longer reads I think that the 454 data deposited in GenBank, even if not always as long as ESTs could also greatly enrich the proteomics analysis and since I would suggest the authors aim to analyse the proteomics data with current more extensive resources, this could also use the 454 data (even without assembly).

Response 3:
We must point out that we did not exclusively use publicly available Lymnaea EST data to perform the Mascot searches (this would have returned far fewer hits than what we were able to achieve). Importantly we generated two Illumina mantle tissue transcriptomes (as stated on page

7 of the methods and reported on in detail later on in the methods on page 9 under the title "NGS sequencing") and used these combined datasets to perform the Mascot searches. This is stated on page 3, lines 64-65 and page 7, line 159 and then described in detail on page 9 under "NGS sequencing". For example:

"Here we have combined an NGS transcriptome analysis of adult mantle tissue with a proteomic survey of the adult shell…"

These L. stagnalis tissue specific databases effectively make any NCBI database search redundant and less informative. To emphasise and validate this point, no MS spectra returned a hit against an NCBI entry that did not also return a hit against our transcriptome data. This is reflected by the BUSCO completeness scores of the mantle transcriptomes (97.9% and 98.4%). Because reviewer 3 also mentions this point we realise that including the NCBI search date is misleading, so we have decided to remove it from the manuscript. The relevant passage now reads as follows:

"Protein identification was performed using the MASCOT search engine (version 2.1; Matrix Science, London, UK) against translations in all 6 frames of our mantle transcriptomes which possessed BUSCO completeness scores of >98% (see below)."

Comment 4:
c) a series of programs are used to assess the putative proteins identified by proteomics. I have several doubts in relation to how this was done. It would be helpful to have an indication of the Mascot parameters/ cut-off for peptide/protein identification. It would have been helpful to have an indication of the proportion of peptides coming from the proteomics that were used for protein identification.

Response 4:
We thank the reviewer for pointing this out. These details have now been included under the heading "Mass spectrometry data analysis" in the methods section on page 7 as follows:

"The peptide hits (protein score >50; FDR <0.05; 1 missed cleavage allowed) were manually confirmed by the observation of the raw LC-MS/MS spectra with ANALYST QS software (Version 1.1)."

Furthermore, for each protein, all scores and peptides used for identification are listed in the new Additional table 43 as now stated here:

"From these 329 matches a total of 40 shell-forming candidate transcripts were identified (see Additional file 43)."

Comment 5:
d) The following phrase could be clearer; (ln 189); Only candidates yielding an mRNA coverage of >98% and an overall identity of >98% are documented…
The coverage and identity are estimated by comparison with what?

Response 5:
We have clarified this statement by specifying that these were BLASTn searches (and therefore are the coverage and overall identity values standardly reported by BLAST searches).

Comment 6:
e) The question in relation to the transcriptomes mentioned in ln 185, totally lost me, I just could not find those transcriptomes in this MS.
Similar transcripts were retrieved from the transcriptomes of the mantle zone 1-5, mantle zone 5, cephalic tentacle, cephalic lobe, CNS, foot, buccal mass and larvae 42 hours post first cleavage (hpfc), 52 hpfc and 67 hpfc.
It is unclear where this information comes from in the methods; is it part of a previous study? Please provide a citation to previous work or explain how this conclusion was reached since in the present study the NGS was performed on total RNA was extracted from the mantle edge and the proximal mantle tissue of a single adult.

Response 6:
This information was accidentally omitted. We have now included the following in the methods section (lines 225-230) to address this issue:

“In addition, transcriptomes from 5 adult tissues (cephalic tentacle, cephalic lobe, CNS, foot and buccal mass) and 3 larval stages (42 hours post first cleavage (hpfc), 52 hpfc and 67 hpfc) were similarly sequenced and assembled. These transcriptomes were used to assess the tissue specific alternative splicing characteristics of all shell-forming genes. All raw NGS data has been deposited in the SRA with BioSample accession numbers SAMN08117214, SAMN08117215, SAMN08709370, SAMN08709371, SAMN08709372, SAMN08709373, SAMN08709374, SAMN08709375, SAMN08709376 and SAMN08709377.”

Comment 7:
f) Please provide a brief description in the methods of the program and parameters used for the NGS assembly and the date to facilitate reading of the MS.

Response 7:
We have now included the following in the methods section (lines 221-224) to address this issue:

“Briefly, raw data was adapter trimmed and quality filtered using Trimmomatic V0.32. Filtered reads were then assembled with Trinity V2.0.3, CLC Genomics Workbench de novo assembler (V8.5), and IDBA-tran. The resulting assemblies were then merged and filtered for redundancy using our pipeline [44]”

Comment 8:
2. Results
g) Remove strikingly, it is predictable that the highest similarity of sequences is between the phylogenetically proximate species. "Strikingly, the highest degree of overall similarity was found with the shell forming proteome of the common groove snail C. nemoralis (Fig. 4), the closest phylogenetic relative"….

Response 8:
In fact this is not a predictable result - we previously observed the exact opposite; a gastropod and a bivalve had more shell-forming sequences in common than the 2 gastropods compared (see Jackson, D.J., et al., 2010. Mol Biol Evol 27, 591–608). However our intention was not to emphasise that the 2 phylogenetically closest representatives shared the most similarity, rather that the degree of similarity between the pair sharing the most similarity and the next pair was so striking (i.e. 28% of C. nemoralis and L. stagnalis and then 15.9% between L. gigantea and L. stagnalis). We have therefore reworded the relevant passage as follows:

"The highest degree of overall similarity was found with the shell forming proteome of the common groove snail C. nemoralis (Fig. 4), the closest phylogenetic relative to L. stagnalis of all species in this comparison. The next highest level of similarity shared with the L. stagnalis shell forming proteome (15.9% of the L. gigantea shell forming proteome) was markedly lower."

Comment 9:
3. Supplementary files
There are a vast number of supplementary files and took me quite some time to download them all. I suggest the authors aim to build plates of the tissue distribution and include several riboprobe per plate, this would make it easier to evaluate and compare the tissue distribution of the various riboprobes.

Response 9:
As recommended by the editor we are working with the editorial staff at GigaScienceDB to submit the supplementary material to a dedicated GigaDB repository. This should make accessing the supplementary material easier. We have also included a file that summarises the content of all of the supplementary data.

Comments/responses 10:
There are a lot of small corrections that could be made, a few are indicated below:
Ln 324; shared significant - corrected.
Ln 326; nine showed similarity - corrected.
Ln 327; them were shared - corrected.
Supplementary file 37 - ribiprobe, presumably this should be riboprobe? - corrected.

We have also corrected other typos and errors found throughout the manuscript. For example:

"insulin like" to "insulin-like" (page 21, lines 482)

"However, without further validation genes …" to "However without further validation, genes identified in this way …" (page 2, line 48)

"This conventional hydrolytic duration allows for the release of most monosaccharides…" to "This treatment allows for the release of most monosaccharides…" (page 9, lines 199-200).

We previously referred to "Additional Tables S1 and S7" (page 19, lines 447-448). This has been corrected to Additional files, 35, 38 and 39).

We previously did not describe or discuss the glycosylation analysis described in the methods and discussion sections. This has now been corrected with the addition of the following:

"Glycosylated proteins (Results section)
The monosaccharides profiles of ASMs and AIMs extracted from adult L. stagnalis shells were peculiar, with less than half of the dozen standard monosaccharides represented. For ASM, these include galactosamine, glucosamine, galactose, glucuronic acid and glucose, while AIM lacked glucuronic acid (Table 1). We also found marked differences in the glycosylation rates of acid-soluble and acid-insoluble matrices. In general the absolute degree of glycosylation of the ASM was 1-3 orders of magnitude greater that of the AIM. The largest difference was in the amount of galactosamine (10.6 ng/µL vs. 0.03 ng/µL). While glucosamine was more abundant absolutely in the ASM (9.41 ng/µL vs. 0.13 ng/µL), the proportional difference was not so extreme (34.8% vs. 54.2%)."

"Glycosylated proteins (Discussion section)
Our preliminary analysis of the sugar moieties attached to shell-forming proteins revealed an interesting dichotomy between the ASM and AIM; the population of ASM proteins appear to be far more glycosylated than AIM proteins (Table 1). Whether this difference is generated by a heavily glycosylated subset of the ASM, or if it reflects a general trend of most ASM proteins being glycosylated remains unknown. We also cannot determine whether there are any spatial biases within the adult mantle tissue with regards to the location of glycosylated proteins. The high percentage of glucosamine identified in AIM and ASM suggests that chitin, or its deacetylated derivative chitosan, is present in both extracts but this hypothesis requires further testing. Despite their likely importance to the functional mechanisms of shell formation, post-translational modifications of molluscan shell-forming proteins remain relatively understudied, and we predict that research efforts in these directions would yield interesting functional insights into the mechanisms of shell fabrication."

Reviewer Linlin Zhang:

Ines Herlitze and co-authors submitted a manuscript titled "Molecular modularity and asymmetry of the molluscan mantle revealed by a gene expression atlas" for review. This manuscript identified 34 candidate genes associated with shell formation by Mass spectrometry based proteomic analysis of L. stagnalis shells. They then demonstrate asymmetry expression pattern during early development and mantle spatial expression pattern of those candidates. Finally, they compared sequence similarity of shell formation genes from metazoan biomineralizing proteomes. This manuscript is well written, and the result of spatial modularity in adult mantle is of great interest.

Major comments:

Comment 11:
1. This manuscript brings up mantle spatial modularity and provides hypothesis that spatial expression of shell-formation genes may lead to shell morphological diversity. This is interesting

and may be the case, but I think the authors need to add some nuance to their argument. Specifically, 41 shell formation candidates (31 showed in situ expression) were identified from shell proteome, and all of those genes are effectors. Comparison analyses in this manuscript indicate the majority of those candidates are lineage specific expansion. I think the result should spend a little more 'ink' on the conservation of shell formation genes. Mantle spatial expression in different phylogeny might also be needed to show the deep evolutionary history behind those lineage specific shell formation genes. This also brings up my next comment.

Response 11:
This is an interesting point and a theme that we are very much interested in (indeed several authors on this paper have spent some time and effort collecting shell-forming proteomes from a diversity of molluscs in order to perform these types of analyses). We therefore appreciate that the reviewer has recognised the value and importance of such an endeavour. However we think that to pursue such a line of investigation in the current manuscript would dilute the main message that we are attempting to bring across; that it is the modular nature of the spatial expression patterns of these genes (lineage specific, deeply conserved or otherwise) in the mantle that supported evolvability of the molluscan shell. Our intent was that Figure 4 would cover the theme of sequence conservation vs. lineage specific. Nonetheless the reviewer is absolutely correct that spatial gene expression analysis of shell-forming genes in more molluscan species would further elucidate the evolutionary history of this structure, and so we have included the following passage:

“Characterisation of the spatial expression patterns of shell-forming proteomes from a selection of other molluscan lineages would also contribute to a more refined understanding of shell evolution.” (page 16, lines 375-377).

Comment 12:
2. The two types of genes involved in shell pattern diversity are patterning genes and effectors. To support the conclusion that mantle spatial modularity and morphology homology explain shell diversity, I suggest the authors to compare mantle spatial gene expression (e.g. their RNAseq data from mantle edge and proximal mantle) and further to look at potential interesting patterning genes (e.g. ligands, transcription factors and so on).

Response 12:
This is also a topic worthy of investigation and indeed we are already making steps in that direction (as we indicate on page 10 with the following passage: “In addition transcriptomes from 5 adult tissues (cephalic tentacle, cephalic lobe, CNS, foot and buccal mass) and 3 larval stages (42 hours post first cleavage (hpfc), 52 hpfc and 67 hpfc) were similarly sequenced and assembled and form the basis of on-going work.”). However we would again suggest that to included such a large analysis in the current manuscript would overly dilute the main message we wish to convey.

Other comments:

Comment 13:
1. The Mass spec and RNAseq data seem out of place. I think the beginning of results should add

summary of the Mass spec and RNAseq data. How many genes they identified from Mass spec and RNAseq data, what are those genes, any overlap between Mass spec and RNAseq? The current supplementary text provided some helpful information.

Response 13:
Good point. We have now rearranged some of the results section and now start with a description of the proteomic (mass spec.) analyses as follows:

“More than 1,230 peptides were analyzed by HPLC-mass spectrometry and subsequently used for protein identification using Mascot against our translated mantle transcriptomes. Of these 1,230 peptides 329 of them returned significant matches. From these 329 matches a total of 41 shell-forming candidate transcripts were identified.”

Comment 14:
2. Line 461-479. Immunoglobulin-like domain does not necessary indicate a role for immunity. e.g. NCAM is the neural cell adhesion molecules.

Response 14:
True, we have no evidence that the proteins we discuss actually have any immunological or cell signalling function and have to rely on the fact that the domains they posses reflect in part their functions. We have tempered this aspect of the discussion with the following passage:

“This interesting hypothesis awaits the development of gene specific knock-down or knock-out assays.” (page 21, lines 496-497).

Comment 15:
3. Figure 2, what is panel B? No legend on this panel.

Response 15:
The legend for this figure was incorrect, thanks for pointing this out, now corrected as follows:

Fig. 2. Overview of the adult L. stagnalis shell-forming mantle tissue and representative shell-forming gene expression patterns that reveal its molecular modularity. A. A semi-thin sagittal section of an adult L. stagnalis stained with Giemsa. The foot (f), mantle (m), digestive gland (dg) and radula (r) are indicated. The mantle tissue is a thin sheet of epithelium that covers the dorsal surface of the adult animal and is responsible for fabricating the shell. B. A magnified view of the red-boxed region in A reveals some of the cellular morphology of the adult mantle tissue. C. A schematic representation of the mantle tissue divided into 6 zones as described by Timmermans [58]. The spatial distribution of enzymatic activities and biochemicals indicated in this schematic are adapted from [58]. We detect a sub-regionalisation of the pallial groove (zone 1) into proximal (light green) and distal (dark green) zones. D. The spatial expression patterns of 8 representative shell-forming genes in the adult mantle tissue. The asterisk indicates that sfc-6 was identified using in silico methods rather than proteomic methods.

Reviewer: Melody Clark:

This is a really well written paper describing a comprehensive investigation into biomineralization genes in L. stagnalis. It is succinctly written for such an extensive dataset. I just have a few comments on the manuscript:

Comment 16:
L24: there is a line missing in the beginning of the abstract of the word document compared with the Gigascience generated abstract.

Response 16:
Corrected.

Comment 17:
L24: The abstract contains quite a few generic sentences, I wonder if it could be re-written slightly to bring out the authors finding more? As at the moment I do not think the abstract does full credit to the work.

Response 17:
We agree and have now included several additional sentences that hopefully convey some of the main findings of our work.

Comment 18:
L107-109: duplication of L97-99. Please delete.

Response 18:
Corrected.

Comment 19:
L157: can the authors justify why the protein identification database generated from ESTs downloaded from NCBI is dated 31st August 2012? This is rather out of date, given we are now in 2018 and the data will have changed? I suspect it is just that the work has taken a long time, but maybe a sentence alongside the data, as a brief explanation?

Response 19:
This point was also raised by another reviewer. Please refer our response to comment 3.

Comment 20:
L185-190: it is unclear to me whether these transcriptomes have been submitted to the databases. They should be submitted and if they occur in a previous paper, then perhaps just refer to either than or add the accession number.

Response 20:
This point was also raised by another reviewer. Please refer to our response to comment 6.

Comment 21:
L225-236: this is a personal opinion, but I think this listing would be clearer as a table rather

than a paragraph of text.

Response 21:
We understand this point and in fact did prepare a table with this information, however the table looked somewhat “anaemic” and we reverted back to including the information in the text. We hope the reviewer concurs.

Comment 22:
L273: detect should read detected

Response 22:
Corrected.

Comment 23:
L282-294: I think this section is slightly confusing in terms of the number of genes used in the in situ studies. I realise, after re-reading that the 34 genes for in situ come from the original 31 (71%) plus 3 in silico candidates. I am presuming that extensive in situ was carried out on 7 of the 41 candidates and although they did not hybridize to any shell forming tissue it would be interesting to know which tissues they produced signals in (supplementary information?).

Response 23:
We have re-worded this section to clarity this point as follows:

“A total of 41 shell-forming candidate transcripts were then identified by our proteomic analyses of the L. stagnalis shell. Of these 41 gene products, 31 (76%) could be cloned and exhibit in situ hybridisation signals compatible with a role in shell formation (either in larval stages and/or in the adult mantle tissue). Seven of these 41 candidates (17%) could be cloned from L. stagnalis cDNA, but did not produce a positive or consistent in situ signal in any tissue. Three of the 41 candidate genes (7%) could not be amplified by gene specific PCR or RACE PCR which we interpret to be most likely the result of bioinformatic errors. In addition to the 31 proteomically identified candidates that generated positive in situ signals, three candidates that were identified via in silico methods (based purely on the presence of a signal sequence and their glycine-rich protein sequences) also generated in situ signals compatible with a role in shell formation and are reported here.”

Comment 24:
L288: any idea why these genes did not clone? Sequencing errors?

Response 24:
We have included the following passage to address this comment:

“Three of the 41 candidate genes (7%) could not be amplified by gene specific PCR or RACE PCR which we interpret to be most likely the result of bioinformatic errors.”

It is a really nice piece of work: I enjoyed reading it and I think it makes a very valuable contribution to the field.

Great - glad you like it!

Reviewer #4, Gaspar Jekely:

This is a very nice and comprehensive paper on putative shell forming genes in the gastropod Lymnaea stagnalis. The authors combined proteomics, transcriptomics, bioinformatic analyses and in situ hybridisation to identify and characterise shell forming genes. The data are of very high quality and this resource will undoubtedly stimulate further work in shell forming genes in conchiferan molluscs.

The quality of the in situ hybridisations is superb and the authors did an impressive job in analysing a large number of genes with this technique.

I only have a few minor comments to improve the manuscript.

Minor comments:

Comment 25:
The species name Lymnaea stagnalis should be added either to the title or the abstract or both. It is not clear from reading the abstract which species was studied.

Response 25:
Good point! We have corrected this by including the name in the abstact.

Comment 26:
duplicated sentences: "L. stagnalis does not fall under the German animal protection act §8 and is listed as 'least concern' under the IUCN's list of threatened species. This work was therefore exempt from the University of Goettingen Ethics Committee."

Response 26:
Corrected.

Comment 27:
"Strikingly, the highest degree of overall similarity was found with the shell forming proteome of the common groove snail C. nemoralis (Fig. 4), the closest phylogenetic relative to L. stagnalis of all species in this comparison"
Why is this striking? This is expected, since this species is the closest relative.

Response 27:
This point was also raised by another reviewer. Please refer to our response to comment 8.

Comment 28:
The section in the Discussion "Alternative splicing increases the diversity of shell-forming

proteins" or at least a large part of it would be more suitable as part of the "Results" section

Response 28:
Agreed, we have moved this section into the Results.

Comment 29:
The 879 biomineralising proteins should be provided as a separate FASTA file.

Response 29:
Now supplied as Additional file 39.

Comment 30:
Additional files 1-34: it is not clear which file belongs to which gene. The gene/protein IDs should be added to the figures, before the sequence.

Response 30:
Gene names have been added into the relevant Additional File figures.
