# Supplementary material for: Molecular modularity and asymmetry of the molluscan mantle revealed by a gene expression atlas
Source: Gigascience. 2018 May 17;7(6):giy056. doi: 10.1093/gigascience/giy056 (PMC6007483; doi:10.1093/gigascience/giy056)
Supplement: Response-to-Reviewer-Comments_Revision-1.pdf [file giy056_response-to-reviewer-comments_revision-1.pdf]

Author's Response To Reviewer Comments

N/A (proxy upload by HZ)
