# Supplementary material for: Molecular modularity and asymmetry of the molluscan mantle revealed by a gene expression atlas
Source: Gigascience. 2018 May 17;7(6):giy056. doi: 10.1093/gigascience/giy056 (PMC6007483; doi:10.1093/gigascience/giy056)
Supplement: Reviewer-1-Report_Original-Submission -- Linlin Zhang, Ph.D. — 16 Feb 2018 Reviewed [file giy056_reviewer-1-report_original-submission.pdf]

Reviewer Report

Title: Molecular modularity and asymmetry of the molluscan mantle revealed by a gene expression atlas

Version: Original Submission **Date:** 16 Feb 2018

Reviewer name: Linlin Zhang, Ph.D.

Reviewer Comments to Author:

Ines Herlitze and coauthors submitted a manuscript titled "Molecular modularity and asymmetry of the molluscan mantle revealed by a gene expression atlas" for review. This manuscript identified 34 candidate genes associated with shell formation by Mass spectrometry based proteomic analysis of L. stagnialis shells. They then demonstrate asymmetry expression pattern during early development and mantle spatial expression pattern of those candidates. Finally, they compared sequence similarity of shell formation genes from metazoan biomineralizing proteomes. This manuscript is well written, and the result of spatial modularity in adult mantle is of great interest.

Major comments:

1. This manuscript brings up mantle spatial modularity and provides hypothesis that spatial expression of shell-formation genes may lead to shell morphological diversity. This is interesting and may be the case, but I think the authors need to add some nuance to their argument. Specifically, 41 shell formation candidates (31 showed in situ expression) were identified from shell proteome, and all of those genes are effectors. Comparison analyses in this manuscript indicate the majority of those candidates are lineage specific expansion. I think the result should spend a little more 'ink' on the conservation of shell formation genes. Mantle spatial expression in different phylogeny might also be needed to show the deep evolutionary history behind those lineage specific shell formation genes. This also brings up my next comment.

2. The two types of genes involved in shell pattern diversity are patterning genes and effectors. To support the conclusion that mantle spatial modularity and morphology homology explain shell diversity, I suggest the authors to compare mantle spatial gene expression (eg. their RNAseq data from mantle edge and proximal mantle) and further to look at potential interesting patterning genes (eg. ligands, transcription factors and so on).

Other comments:

1. The Mass spec and RNAseq data seem out of place. I think the beginning of results should add summary of the Mass spec and RNAseq data. How many genes they identified from Mass spec and RNAseq data, what are those genes, any overlap between Mass spec and RNAseq? The current supplementary text provided some helpful information.

2. Line 461-479. Immunoglobulin-like domain does not necessary indicate a role for immunity. eg. NCAM is the neural cell adhesion molecules.

3. Figure 2, what is panel B? No legend on this panel.

Methods

Are the methods appropriate to the aims of the study, are they well described, and are necessary controls included? Yes

Conclusions

Are the conclusions adequately supported by the data shown? No

Reporting Standards

Does the manuscript adhere to the journal's guidelines on minimum standards of reporting? Yes

Statistics

Are you able to assess all statistics in the manuscript, including the appropriateness of statistical tests used? Yes, and I have assessed the statistics in my report.

Quality of Written English

Please indicate the quality of language in the manuscript: Acceptable

Declaration of Competing Interests

Please complete a declaration of competing interests, considering the following questions:

- Have you in the past five years received reimbursements, fees, funding, or salary from an organisation that may in any way gain or lose financially from the publication of this manuscript, either now or in the future?
- Do you hold any stocks or shares in an organisation that may in any way gain or lose financially from the publication of this manuscript, either now or in the future?
- Do you hold or are you currently applying for any patents relating to the content of the manuscript?
- Have you received reimbursements, fees, funding, or salary from an organization that holds or has applied for patents relating to the content of the manuscript?
- Do you have any other financial competing interests?

- Do you have any non-financial competing interests in relation to this paper?

If you can answer no to all of the above, write 'I declare that I have no competing interests' below. If your reply is yes to any, please give details below.

I declare that I have no competing interests

I agree to the open peer review policy of the journal. I understand that my name will be included on my report to the authors and, if the manuscript is accepted for publication, my named report including any attachments I upload will be posted on the website along with the authors' responses. I agree for my report to be made available under an Open Access Creative Commons CC-BY license (http://creativecommons.org/licenses/by/4.0/). I understand that any comments which I do not wish to be included in my named report can be included as confidential comments to the editors, which will not be published.

I agree to the open peer review policy of the journal

To further support our reviewers, we have joined with Publons, where you can gain additional credit to further highlight your hard work (see: https://publons.com/journal/530/gigascience). On publication of this paper, your review will be automatically added to Publons, you can then choose whether or not to claim your Publons credit. I understand this statement.

Yes
