# Supplementary material for: Molecular modularity and asymmetry of the molluscan mantle revealed by a gene expression atlas
Source: Gigascience. 2018 May 17;7(6):giy056. doi: 10.1093/gigascience/giy056 (PMC6007483; doi:10.1093/gigascience/giy056)
Supplement: Reviewer-2-Report_Original-Submission -- Melody Clark — 03 Mar 2018 Reviewed [file giy056_reviewer-2-report_original-submission.pdf]

**Reviewer Report**

**Title:** Molecular modularity and asymmetry of the molluscan mantle revealed by a gene expression atlas

**Version:** Original Submission **Date:** 03 Mar 2018

**Reviewer name:** Melody Clark

**Reviewer Comments to Author:**

This is a really well written paper describing a comprehensive investigation into biomineralization genes in L. stagnalis. It is succinctly written for such an extensive dataset. I just have a few comments on the manuscript:

L24: there is a line missing in the beginning of the abstract of the word document compared with the Gigascience generated abstract.
L24: The abstract contains quite a few generic sentences, I wonder if it could be re-written slightly to bring out the authors finding more? As at the moment I do not think the abstract does full credit to the work.
L107-109: duplication of L97-99. Please delete.
L157: can the authors justify why the protein identification database generated from ESTs downloaded from NCBI is dated 31st August 2012? This is rather out of date, given we are now in 2018 and the data will have changed? I suspect it is just that the work has taken a long time, but maybe a sentence alongside the data, as a brief explanation?
L185-190: it is unclear to me whether these transcriptomes have been submitted to the databases. They should be submitted and if they occur in a previous paper, then perhaps just refer to either than or add the accession number.
L225-236: this is a personal opinion, but I think this listing would be clearer as a table rather than a paragraph of text.
L273: detect should read detected
L282-294: I think this section is slightly confusing in terms of the number of genes used in the in situ studies. I realise, after re-reading that the 34 genes for in situ come from the original 31 (71%) plus 3 in silico candidates. I am presuming that extensive in situ was carried out on 7 of the 41 candidates and although they did not hybridize to any shell forming tissue it would be interesting to know which tissues they produced signals in (supplementary information?).
L288: any idea why these genes did not clone? Sequencing errors?

It is a really nice piece of work: I enjoyed reading it and I think it makes a very valuable contribution to the field.

**Methods**

Are the methods appropriate to the aims of the study, are they well described, and are necessary controls included? Yes

**Conclusions**

Are the conclusions adequately supported by the data shown? Yes

**Reporting Standards**

Does the manuscript adhere to the journal's guidelines on minimum standards of reporting? Yes

**Statistics**

Are you able to assess all statistics in the manuscript, including the appropriateness of statistical tests used? There are no statistics in the manuscript.

**Quality of Written English**

Please indicate the quality of language in the manuscript: Acceptable

**Declaration of Competing Interests**

Please complete a declaration of competing interests, considering the following questions:

- Have you in the past five years received reimbursements, fees, funding, or salary from an organisation that may in any way gain or lose financially from the publication of this manuscript, either now or in the future?
- Do you hold any stocks or shares in an organisation that may in any way gain or lose financially from the publication of this manuscript, either now or in the future?
- Do you hold or are you currently applying for any patents relating to the content of the manuscript?
- Have you received reimbursements, fees, funding, or salary from an organization that holds or has applied for patents relating to the content of the manuscript?
- Do you have any other financial competing interests?
- Do you have any non-financial competing interests in relation to this paper?

If you can answer no to all of the above, write 'I declare that I have no competing interests' below. If your reply is yes to any, please give details below.

I declare that I have no competeing interests

I agree to the open peer review policy of the journal. I understand that my name will be included on my report to the authors and, if the manuscript is accepted for publication, my named report including any

attachments I upload will be posted on the website along with the authors' responses. I agree for my report to be made available under an Open Access Creative Commons CC-BY license (http://creativecommons.org/licenses/by/4.0/). I understand that any comments which I do not wish to be included in my named report can be included as confidential comments to the editors, which will not be published.

I agree to the open peer review policy of the journal

To further support our reviewers, we have joined with Publons, where you can gain additional credit to further highlight your hard work (see: https://publons.com/journal/530/gigascience). On publication of this paper, your review will be automatically added to Publons, you can then choose whether or not to claim your Publons credit. I understand this statement.

Yes
