# Supplementary material for: Molecular modularity and asymmetry of the molluscan mantle revealed by a gene expression atlas
Source: Gigascience. 2018 May 17;7(6):giy056. doi: 10.1093/gigascience/giy056 (PMC6007483; doi:10.1093/gigascience/giy056)
Supplement: Reviewer-3-Report_Original-Submission -- Deborah Power, PhD — 07 Mar 2018 Reviewed [file giy056_reviewer-3-report_original-submission.pdf]

Reviewer Report

Title: Molecular modularity and asymmetry of the molluscan mantle revealed by a gene expression atlas

Version: Original Submission **Date:** 07 Mar 2018

Reviewer name: Deborah Power, PhD

Reviewer Comments to Author:

The MS by Herlitz et al., is exciting and timely as it addresses a fundamental question linked to the role of the mantle in shell plasticity and the evolutionary success of the molluscs. In this comprehensive study the authors use transcriptomics, proteomics and in situ hybridisation to map transcripts and gain insight into the shell secreting tissue and how it links to shell asymmetry. As the authors point out there have now been a number of transcriptomic and in situ studies aimed at identifying the molecules involved in shell construction (frequently in gastropods and also in with some bivalves) but far fewer studies exist of the shell proteome and no studies exist as far as I am aware linking the proteome with the transcriptome. The authors propose that the proteome gives far more compelling evidence of proteins involved in shell formation than transcriptomic data. I agree to some extent with this statement in so far that the proteins are functional. However, I do not think that positional mapping to the mantle is necessarily direct evidence of a function in calcification and so the authors should be a bit more circumspect and avoid categorical statements. The authors clearly and convincingly demonstrate how their study significantly advances the state of the art and the in situ part of the study is of excellent quality. Having said all the above, I think that the MS could be improved. I expected the various subsections in the methods and results to be in the same order but this does not seem to occur; some of the methods are insufficiently described and results pop-up, which are difficult to link with the methods (genomic scaffolds). The results are over descriptive and should be more incisive by going straight to the point. The methods are over brief and cite other articles and so miss out important details needed for quality assessment. In contrast the results go over data previously reported (eg. section 1), while this is useful, I think it could be shortened. In summary, I appreciated this study but the MS should be better written and organised to do full justice to the data and to make the MS easier to read. I outline below some aspects of the study that could be improved.

1. Methods

a) It would be helpful to have a better phylogenetic designation of the species in the methods to allow a better appreciation of the significance of the observations across the molluscs.

b) The proteomics is generally well performed. However, I suggest that the authors update their Mascot searches since this work was conducted in 2012 (31st August, 2012) and since then a lot more data from molluscs has been deposited in NCBI. Furthermore, it is unclear if a species filter was used to search the EST databases used for the proteomics analysis or if all ESTs were used. Taking into consideration the emphasis given in the introduction to the difficulty of making interference about mollusc gene function due to limited sequence conservation with more conventional models I was expecting to see some selection of the EST dataset used for analysis. While I can understand that EST databases may generate

longer reads I think that the 454 data deposited in genebank, even if not always as long as ESTs could also greatly enrich the proteomics analysis and since I would suggest the authors aim to analyse the proteomics data with current more extensive resources, this could also use the 454 data (even without assembly).

c) a series of programs are used to assess the putative proteins identified by proteomics. I have several doubts in relation to how this was done. It would be helpful to have an indication of the Mascot parameters/ cut-off for peptide/protein identification. It would have been helpful to have an indication of the proportion of peptides coming from the proteomics that were used for protein identification.

d) The following phrase could be clearer; (ln 189); Only candidates yielding an mRNA coverage of >98% and an overall identity of >98% are documented.....

The coverage and identity are estimated by comparison with what?

e) The question in relation to the transcriptomes mentioned in ln 185, totally lost me, I just could not find those transcriptomes in this MS.

Similar transcripts were retrieved from the transcriptomes of the mantle zone 1-5, mantle zone 5, cephalic tentacle, cephalic lobe, CNS, foot, buccal mass and larvae 42 hours post first cleavage (hpfc), 52 hpfc and 67 hpfc.

It is unclear where this information comes from in the methods; is it part of a previous study? Please provide a citation to previous work or explain how this conclusion was reached since in the present study the NGS was performed on total RNA was extracted from the mantle edge and the proximal mantle tissue of a single adult.

f) Please provide a brief description in the methods of the program and parameters used for the NGS assembly and the date to facilitate reading of the MS.

2. Results

g) Remove strikingly, it is predictable that the highest similarity of sequences is between the phylogenetically proximate species. "Strikingly, the highest degree of overall similarity was found with the shell forming proteome of the common groove snail C. nemoralis (Fig. 4), the closest phylogenetic relative"....

3. Supplementary files

There are a vast number of supplementary files and took me quite some time to download them all. I suggest the authors aim to build plates of the tissue distribution and include several riboprobe per plate, this would make it easier to evaluate and compare the tissue distribution of the various riboprobes.

There are a lot of small corrections that could be made, a few are indicated below:
Ln 324; shared significant
Ln 326; nine showed similarity
Ln 327; them were shared
Supllementary file 37 - ribiprobe, presumably this should be riboprobe?

Methods

Are the methods appropriate to the aims of the study, are they well described, and are necessary controls included? Yes

Conclusions

Are the conclusions adequately supported by the data shown? Yes

Reporting Standards

Does the manuscript adhere to the journal's guidelines on minimum standards of reporting? Yes

Statistics

Are you able to assess all statistics in the manuscript, including the appropriateness of statistical tests used? There are no statistics in the manuscript.

Quality of Written English

Please indicate the quality of language in the manuscript: Acceptable

Declaration of Competing Interests

Please complete a declaration of competing interests, considering the following questions:

- Have you in the past five years received reimbursements, fees, funding, or salary from an organisation that may in any way gain or lose financially from the publication of this manuscript, either now or in the future?
- Do you hold any stocks or shares in an organisation that may in any way gain or lose financially from the publication of this manuscript, either now or in the future?
- Do you hold or are you currently applying for any patents relating to the content of the manuscript?
- Have you received reimbursements, fees, funding, or salary from an organization that holds or has applied for patents relating to the content of the manuscript?
- Do you have any other financial competing interests?
- Do you have any non-financial competing interests in relation to this paper?

If you can answer no to all of the above, write 'I declare that I have no competing interests' below. If your reply is yes to any, please give details below.

I am working on the same general subject the molluscan mantle and its role in shell production. Other than this I have no competing interests.

I agree to the open peer review policy of the journal. I understand that my name will be included on my report to the authors and, if the manuscript is accepted for publication, my named report including any attachments I upload will be posted on the website along with the authors' responses. I agree for my report to be made available under an Open Access Creative Commons CC-BY license (http://creativecommons.org/licenses/by/4.0/). I understand that any comments which I do not wish to be included in my named report can be included as confidential comments to the editors, which will not be published.

I agree to the open peer review policy of the journal

To further support our reviewers, we have joined with Publons, where you can gain additional credit to further highlight your hard work (see: https://publons.com/journal/530/gigascience). On publication of this paper, your review will be automatically added to Publons, you can then choose whether or not to claim your Publons credit. I understand this statement.

Yes
