# Supplementary material for: Molecular modularity and asymmetry of the molluscan mantle revealed by a gene expression atlas
Source: Gigascience. 2018 May 17;7(6):giy056. doi: 10.1093/gigascience/giy056 (PMC6007483; doi:10.1093/gigascience/giy056)
Supplement: Reviewer-4-Report_Original-Submission -- Gaspar Jekely — 13 Mar 2018 Reviewed [file giy056_reviewer-4-report_original-submission.pdf]

Reviewer Report

Title: Molecular modularity and asymmetry of the molluscan mantle revealed by a gene expression atlas

Version: Original Submission **Date:** 13 Mar 2018

Reviewer name: Gaspar Jekely

Reviewer Comments to Author:

This is a very nice and comprehensive paper on putative shell forming genes in the gastropod Lymnaea stagnalis. The authors combined proteomics, transcriptomics, bioinformatic analyses and in situ hybridisation to identify and characterise shell forming genes. The data are of very high quality and this resource will undoubtedly stimulate further work in shell forming genes in conchiferan molluscs.
The quality of the in situ hybridisations is superb and the authors did an impressive job in analysing a large number of genes with this technique.

I only have a few minor comments to improve the manuscript.

Minor comments:

The species name Lymnaea stagnalis should be added either to the title or the abstract or both. It is not clear from reading the abstract which species was studied.

duplicated sentences: "L. stagnalis does not fall under the German animal protection act §8 and is listed as 'least concern' under the IUCN's list of threatened species. This work was therefore exempt from the University of Goettingen Ethics Committee."

"Strikingly, the highest degree of overall similarity was found with the shell forming proteome of the common groove snail C. nemoralis (Fig. 4), the closest phylogenetic relative to L. stagnalis of all species in this comparison"
Why is this striking? This is expected, since this species is the closest relative.

The section in the Discussion "Alternative splicing increases the diversity of shell-forming proteins" or at least a large part of it would be more suitable as part of the "Results" section

The 879 biomineralising proteins should be provided as a separate FASTA file.

Additional files 1-34: it is not clear which file belongs to which gene. The gene/protein IDs should be added to the figures, before the sequence

Methods

Are the methods appropriate to the aims of the study, are they well described, and are necessary controls included? NO

Conclusions

Are the conclusions adequately supported by the data shown? YES

Reporting Standards

Does the manuscript adhere to the journal's guidelines on minimum standards of reporting? YES

Statistics

Are you able to assess all statistics in the manuscript, including the appropriateness of statistical tests used? Yes, and I have assessed the statistics in my report.

Quality of Written English

Please indicate the quality of language in the manuscript: Acceptable

Declaration of Competing Interests

Please complete a declaration of competing interests, considering the following questions:

- Have you in the past five years received reimbursements, fees, funding, or salary from an organisation that may in any way gain or lose financially from the publication of this manuscript, either now or in the future?
- Do you hold any stocks or shares in an organisation that may in any way gain or lose financially from the publication of this manuscript, either now or in the future?
- Do you hold or are you currently applying for any patents relating to the content of the manuscript?
- Have you received reimbursements, fees, funding, or salary from an organization that holds or has applied for patents relating to the content of the manuscript?
- Do you have any other financial competing interests?
- Do you have any non-financial competing interests in relation to this paper?

If you can answer no to all of the above, write 'I declare that I have no competing interests' below. If your reply is yes to any, please give details below.

I declare that I have no competing interests

I agree to the open peer review policy of the journal. I understand that my name will be included on my report to the authors and, if the manuscript is accepted for publication, my named report including any attachments I upload will be posted on the website along with the authors' responses. I agree for my report to be made available under an Open Access Creative Commons CC-BY license (http://creativecommons.org/licenses/by/4.0/). I understand that any comments which I do not wish to be included in my named report can be included as confidential comments to the editors, which will not be published.

I agree to the open peer review policy of the journal

To further support our reviewers, we have joined with Publons, where you can gain additional credit to further highlight your hard work (see: https://publons.com/journal/530/gigascience). On publication of this paper, your review will be automatically added to Publons, you can then choose whether or not to claim your Publons credit. I understand this statement.

Yes
